# Mentalizing impairments across 11 psychiatric conditions: A transdiagnostic systematic review and network meta-analysis of tasks with static illustrations

**DOI:** 10.1192/j.eurpsy.2025.10146

**Published:** 2025-12-23

**Authors:** Harry Kam Hung Tsui, Jace Chi Ching Lo, Sherry Kit Wa Chan

**Affiliations:** 1 Department of Psychiatry, Li Ka Shing Faculty of Medicine, The University of Hong Kong, Hong Kong SAR; 2 Department of Psychiatry, Queen Mary Hospital, Hong Kong SAR

**Keywords:** comic strips, mentalizing, network meta-analysis, static illustration, theory of mind, transdiagnostic

## Abstract

**Background:**

Impairments in mentalizing, or theory of mind, occur across psychiatric disorders. Static illustrations are widely used to assess mentalizing due to their simplicity, and they allow assessment of specific cognitive processes. However, systematic comparisons of impairments between psychiatric disorders, neurodevelopmental disorders, and at-risk groups in mentalizing tasks with static illustrations are currently lacking.

**Methods:**

A systematic review with pairwise and network meta-analyses (NMA) was conducted to evaluate mentalizing impairments using tasks with static illustrations across psychiatric disorders compared to healthy controls (HCs) and between groups. Subgroup analyses examined specific mentalizing domains (false belief, humor, and intentionality), and meta-regression analyses explored potential moderators. The ceiling effects of specific tasks were also examined.

**Results:**

Eighty-nine studies were included, involving 9,038 participants and 11 psychiatric conditions. Significant mentalizing deficits were observed across all conditions versus HCs, except for the familial risk for bipolar disorder group. NMA demonstrated that schizophrenia (*g* = −0.960) and early schizophrenia (*g* = −0.785) exhibited the most pronounced impairments, followed by borderline personality disorder (*g* = −0.612) and obsessive-compulsive disorder (*g* = −0.613). Particularly, schizophrenia showed significantly greater deficits than autism, bipolar disorder, clinical and familial high risk for schizophrenia, and depression. Domain-specific analyses highlighted differential impairment patterns. The presence of prominent ceiling effects suggests major limitations of tasks with static illustrations.

**Conclusions:**

This review provides detailed insights into transdiagnostic and disorder-specific patterns of mentalizing impairments with tasks using static illustrations. Findings highlight the importance of domain-specific approaches, examining interindividual variability, refining assessment tools, and implementing targeted interventions.

## Introduction

Mentalizing, or theory of mind (ToM), refers to the ability to attribute others’ mental states, such as thoughts, desires, and emotions [1]. It is fundamental to social interaction and linked to psychiatric symptoms, social functioning, and social skills [2, 3]. Recent reviews indicated widespread mentalizing impairments across psychiatric conditions [2, 4, 5]. However, it remains unclear whether these deficits reflect shared vulnerabilities or disorder-specific mechanisms. Adopting a transdiagnostic approach in studying mentalizing impairments aligns with contemporary frameworks, such as the Research Domain Criteria initiative [6] and the Hierarchical Taxonomy of Psychopathology model [7], emphasizing the importance of identifying nuanced processes and underlying mechanisms in psychopathology across psychiatric conditions [8, 9]. Exploring shared and unique mentalizing impairments will improve our understanding of the distinct presentations and mechanistic features of social cognitive deficits, thereby informing the development of targeted treatments.

Previous meta-analyses have compared social cognitive impairments between disorders, such as schizophrenia versus autism spectrum disorder (ASD) [10], or stages along the psychosis continuum, including individuals with clinical high-risk for psychosis (CHR), familial high risk for psychosis (FHR), and early schizophrenia [11, 12]. However, traditional pairwise meta-analysis is limited to examining only two groups at a time and cannot simultaneously compare multiple conditions. Network meta-analysis (NMA) emerged as a promising tool derived from graph theory, which has primarily been used to compare multiple treatments [13]. Recently, NMA has been applied to cognitive biases [9] and brain morphology [14] across psychiatric disorders, with distinct conditions represented as nodes. This framework allows simultaneous, statistically coherent comparisons among multiple conditions, yielding a more comprehensive map of mentalizing impairment.

Conceptually, mentalizing is an umbrella term encompassing diverse domains, such as false belief, humor comprehension, and intentionality. Its assessment has relied on varied modalities, including verbal and nonverbal tasks, text-based readings, comic strips, and videos, leading to considerable heterogeneity [15–17]. Recent reviews emphasize the need to analyze these methods separately. For example, Gao et al. [16] showed that ASD adults displayed distinct impairment patterns depending on task type, performing poorly on text-based reading comprehension and video-based ecological scene comprehension tasks, but showing only moderate deficits on perceptual scene comprehension tasks based on static illustrations. Despite such variability in experimental designs and psychometric properties, most meta-analyses have aggregated mentalizing tasks without accounting for these distinctions [17, 18]. Among assessment approaches, tasks using static illustrations or comic strips, such as the commonly used Brüne’s Picture Sequencing Task (PST) [19] and Brunet’s Comic Strip Task (CST) [20], combine visual and narrative elements, making them engaging, accessible, and relatively low in cognitive demand. They can mimic real-life social interactions, minimize language demands, and provide standardized formats for cross-population comparisons. Yet, no systematic review has comprehensively examined mentalizing impairment across psychiatric conditions, focusing specifically on such tasks.

This review aimed to examine static illustration-based mentalizing tasks across conditions to identify shared and disorder-specific patterns of impairments. Pairwise meta-analysis and NMA were conducted to compare psychiatric groups with healthy controls (HCs) and between groups. Subgroup analyses examined impairments across specific task domains, including false belief, humor, and intentionality tasks. Additionally, meta-regression analyses were employed to explore potential moderators related to participant and study characteristics, such as demographics and estimated intelligence. We also assessed the ceiling effects of specific tasks to evaluate the psychometric properties across different samples. By investigating mentalizing deficits across psychiatric groups, this review aims to uncover patterns of impairment that can inform theories of social cognition and contribute to the development of targeted treatment strategies.

## Methods

### Search strategy and eligibility

This systematic review and meta-analysis adhered to Preferred Reporting Items for Systematic Reviews and Meta-Analyses guidelines and was preregistered in PROSPERO (CRD42024629394) (Supplementary Methods). Four electronic databases – Embase, MEDLINE, PsycINFO, and Web of Science – were systematically searched up to February 2, 2025. The search strategy consisted of terms related to nonverbal mentalizing or ToM tasks using static illustrations: (“Attribution of Intention Task” OR “Cartoon Stories ToM Paradigm” OR “Cartoon Theory of Mind” OR “Cartoon Vignette” OR “Comic Strip Task” OR “Comic Theory of Mind” OR “Joke-Appreciation Task” OR “Nonverbal Theory of Mind” OR “Picture Sequencing Task” OR “Picture Stories Task” OR “Theory of Mind Stories Task” OR “Visual Jokes Test” OR “Yoni Task”). Two researchers (H.K.H.T and J.L.) independently conducted the screening, data extraction, and quality assessment procedures between February 2, 2025, and February 17, 2025, with discrepancies resolved through team discussion. Inter-rater reliability was high (Cohen’s *κ* = 0.72–0.81). Detailed inclusion and exclusion criteria are provided in the Supplementary Methods.

### Data extraction

Demographics and clinical details of all groups were recorded, including age, gender, years of education, diagnosis or condition, estimated intelligence quotient (IQ), validated diagnostic or assessment tools used, comorbidity, and medication information. Information about the mentalizing tasks with static illustration was also documented, including behavioral performance metrics, scoring methods, experimental designs, and stimulus characteristics. Tasks were classified into three domains: false belief (understanding beliefs differing from one’s own or reality), intentionality (inferring goal-directed actions or intentions), and humor (detecting or resolving incongruities). This categorization, informed by prior theoretical reviews [16, 21, 22], was verified through an independent review of task protocols and scoring methods. Additional study characteristics (year, author, and country) were recorded, and authors were contacted for missing information.

Participant or diagnostic groups were categorized according to established definitions. Early schizophrenia was defined as first-episode schizophrenia or psychosis, or illness onset within 5 years [23, 24]. This distinction was made to capture studies explicitly targeting the early illness stage, rather than general schizophrenia samples that often include a mix of illness durations. As prior evidence suggests distinct profiles of social cognitive [25], cognitive [26], and neurostructural alterations [27] between early and chronic stages, this distinction aligned with the abundance of studies focusing on first-episode or early-stage schizophrenia, enabling an adequate sample size and examination of potential stage-related differences in mentalizing ability. CHR refers to the high-risk group of developing psychosis identified by validated tools such as the Comprehensive Assessment of At-Risk Mental States and the Structured Interview for Psychosis-Risk Syndromes. FHR-S and FHR-B referred to first-degree relatives of patients with schizophrenia or bipolar disorder, respectively.

### Quality assessment

Study quality was assessed with a modified Newcastle-Ottawa Scale (Supplementary Methods), evaluating diagnostic methods, sample representativeness, group comparability, task validity, and outcome reporting. Composite scores summarized methodological quality and risk of bias.

### Statistical analysis

Effect sizes were calculated as Hedges-adjusted standardized mean difference, where negative values indicated poorer mentalizing (Supplementary Methods), and interpreted as small (0.20), moderate (0.50), and large (0.80) following established guidelines [28]. Pairwise meta-analyses adopted random-effects models with restricted maximum likelihood and inverse-variance weighting to compare each group with HCs, and with one another. Subgroup analyses were conducted by task domains (Intentionality, False Belief, and Humor). Heterogeneity was assessed using *I* ^2^, *Q*, and *τ* ^2^ statistics. Egger’s test was performed to evaluate the potential publication biases. Meta-regression was restricted to pairwise analyses because covariate adjustment is not supported in frequentist NMA, exploring potential moderators, such as demographics, estimated IQ, sample sizes, or quality scores. Only variables with at least four studies were included in meta-regression.

A frequentist random-effects NMA was conducted to compare all groups simultaneously, overall, and stratified by task domains. Nodes represented distinct groups, with only those having at least three studies included in the overall NMA model to ensure statistical power for reliable comparisons. Results were illustrated with network graphs, forest plots, and league tables. Global heterogeneity (*τ* ^2^ and *I* ^2^) and network inconsistency (separating indirect from direct evidence/SIDE test) were assessed (Supplementary Methods). Publication bias was evaluated using comparison-adjusted funnel plots and Egger’s test. Sensitivity analyses were conducted to assess the robustness of the results by excluding studies with a high risk of bias. The confidence in the evidence was evaluated using the Confidence in Network Meta-Analysis (CINeMA) framework. NMA results were prioritized, given greater statistical power from combining direct and indirect evidence than pairwise results.

Ceiling effects were defined as mean scores ≥80% of the maximum [29] and were assessed only for tasks with at least five studies. For each task, the number and percentage of samples exhibiting ceiling effects were calculated, along with mean percentage scores. Statistical analysis was performed in R version 4.4.1 (*metafor* and *netmeta)* with two-tailed *α* = 0.05.

## Results

**Figure 1.**
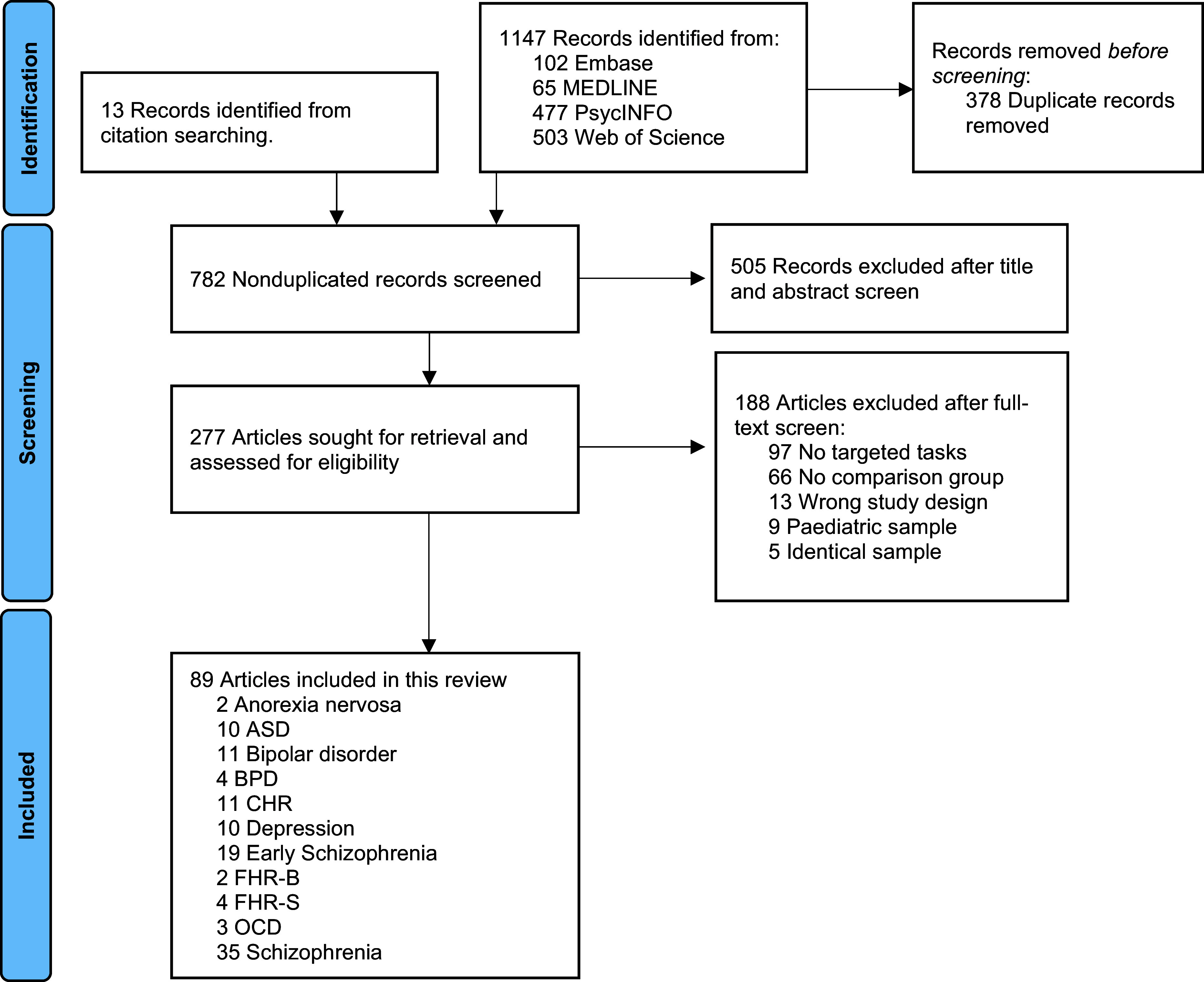
Preferred Reporting Items for Systematic Reviews and Meta-Analyses (PRISMA) flow diagram showing study selection. *Note*: Literature searches were conducted by two independent researchers from database inception until February 2, 2025. ASD, autism spectrum disorders; BPD, borderline personality disorder; CHR, individuals at clinical high risk for psychosis; FHR-B, familial high risk for bipolar disorder; FHR-S, familial high risk for schizophrenia; OCD, obsessive-compulsive disorder.

From 1,488 records screened, 89 studies met the inclusion criteria, comprising 9,038 participants across 11 psychiatric and neurodevelopmental conditions or high-risk groups (Figure 1). The overall sample had a mean age of 32.2 years, with 48.4% female participants (Supplementary Table S1). Schizophrenia (*k* = 35; *N* = 1,850) and early schizophrenia (*k* = 19; *N* = 718) were the most studied conditions, followed by bipolar disorder (*k* = 11, *N* = 366) and CHR (*k* = 11, *N* = 446), ASD (*k* = 10, *N* = 468) and depression (*k* = 10; *N* = 286), borderline personality disorder (BPD) (*k* = 4; *N* = 200) and FHR for schizophrenia (FHR-S (*k* = 4; *N* = 710), obsessive-compulsive disorder (OCD) (*k* = 3; *N* = 150), and anorexia nervosa (*k* = 2; *N* = 42) and FHR for bipolar disorder (FHR-B) (*k* = 2; *N* = 41). No eligible studies were identified for ADHD and general anxiety disorder. While most studies matched age and sex between groups, mean values varied across diagnostic categories (Supplementary Table S2). Nine distinct mentalizing tasks were identified, and details were provided in Supplementary Table S3. Intentionality (*k* = 43) and false belief (*k* = 35) tasks were most common, while humor comprehension was examined in only 11 studies, precluding subgroup NMA. The included studies demonstrated good methodological quality, with a mean modified Newcastle-Ottawa Scale score of 6 (median = 6; range = 4–8).

### Pairwise meta-analysis

Thirty-seven pairwise meta-analyses were conducted comparing psychiatric conditions with HCs and with each other across overall mentalizing performance and three task-type subgroups. All psychiatric conditions demonstrated significant mentalizing impairment compared to HCs, except for the FHR-B group, with varying patterns across task types (Table 1 and Supplementary Figure S1). Particularly, only ASD, anorexia nervosa, BPD, and schizophrenia were impaired in humor tasks, although very few studies were available. Between-condition comparisons only revealed that schizophrenia showed greater impairment than depression (*g* = 0.565 [0.327–0.804], *p* < 0.001), and early schizophrenia showed greater impairment than CHR (*g* = 0.430 [0.047–0.814], *p* = 0.028). The main analyses showed moderate heterogeneity with a mean *I* ^2^ of 38.7% and a mean *τ* ^2^ of 0.080. High heterogeneity (*I* ^2^ > 75%, *τ* ^2^ > 0.160) was observed in comparisons between bipolar disorder, FHR-S, and schizophrenia with HCs.

**Table 1.** Stratified meta-analyses (or single effect size) of mentalizing ability with static illustrations across psychiatric conditions compared to healthy controls, and between conditions

			95% CI							
Variable	*k*	Hedge’s *g*	Lower	Upper	*p*	*Q*-value	*p*Q	*τ* ^2^	*I* ^2^ (%)	*p*Egger
**ASD vs. HC**										
Overall	10	−0.402	−0.668	−0.135	**0.003**	37.152	**<0.001**	0.127	74.49	0.130
Intentionality	6	−0.348	−0.751	0.055	0.090	21.723	**<0.001**	0.184	78.9	0.080
False belief	2	−0.283	−0.847	0.281	0.325	3.051	0.081	0.112	67.23	/
Humor	2	−0.672	−0.918	−0.426	**<0.001**	0.074	0.786	0.000	0.00	**/**
**Bipolar disorder vs. HC**										
Overall	10	−0.355	−0.700	−0.011	**0.043**	38.930	**<0.001**	0.240	79.42	0.223
Intentionality	3	−0.370	−1.355	0.614	0.461	23.603	**<0.001**	0.693	91.67	**0.039**
False belief	6	−0.405	−0.724	−0.085	**0.013**	13.048	**0.023**	0.099	63.41	**0.016**
Humor	1	0.195	−0.549	0.940	0.607	/				
**BPD vs. HC**										
Overall	4	−0.543	−0.800	−0.287	**<0.001**	3.991	0.262	0.012	17.79	0.815
Intentionality	2	−0.408	−0.954	0.139	0.144	2.209	0.137	0.085	54.73	/
False belief	1	−0.517	−0.899	−0.135	**0.008**	/				
Humor	1	−0.809	−1.277	−0.341	**<0.001**	/				
**CHR vs. HC**										
Overall	11	−0.422	−0.587	−0.257	**<0.001**	11.769	0.301	0.011	14.51	0.269
Intentionality	7	−0.527	−0.730	−0.342	**<0.001**	6.522	0.367	0.011	14.14	0.298
False belief	2	−0.317	−0.628	−0.007	**0.045**	0.943	0.332	0.000	0.00	**/**
Humor	2	−0.077	−0.482	0.328	0.710	0.001	0.977	0.000		
**Depression vs. HC**										
Overall	10	−0.426	−0.634	−0.218	**<0.001**	14.836	0.096	0.045	40.08	0.852
Intentionality	3	−0.751	−1.042	−0.461	**<0.001**	0.520	0.771	0.000	0.00	0.971
False belief	6	−0.321	−0.569	−0.072	**<0.001**	6.988	0.222	0.028	29.05	0.840
Humor	1	−0.129	−0.598	0.340	0.589	/				
**Early schizophrenia vs. HC**										
Overall	20	−0.786	−0.962	−0.611	**<0.001**	43.770	**0.001**	0.088	56.41	**0.008**
Intentionality	9	−0.920	−1.153	−0.688	**<0.001**	13.105	**<0.001**	0.050	40.03	0.306
False belief	9	−0.626	−0.866	−0.386	**<0.001**	17.344	**0.027**	0.071	53.83	0.284
Humor	2	−1.011	−2.102	0.079	0.069	6.657	**0.010**	0.528	84.98	**/**
**FHR-S vs. HC**										
Overall	5	−0.641	−0.883	−0.039	**0.032**	29.455	**<0.001**	0.187	85.34	**0.046**
Intentionality	3	−0.785	−1.050	−0.520	**<0.001**	1.424	0.491	0.000	0.00	0.280
False belief	2	0.013	−0.118	0.145	0.843	0.014	0.905	0.000	0.00	/
Humor	0	/								
**OCD vs. HC**										
Overall	3	−0.621	−0.854	−0.389	**<0.001**	0.083	0.959	0.000	0.00	0.821
Intentionality	1	−0.610	−1.128	−0.093	**0.021**	/				
False belief	2	−0.624	−0.885	−0.364	**<0.001**	0.081	0.777	0.000	0.00	/
Humor	0	/								
**Schizophrenia vs. HC**										
Overall	37	−0.982	−1.182	−0.782	**<0.001**	212.269	**<0.001**	0.301	84.72	**<0.001**
Intentionality	17	−1.046	−1.355	−0.737	**<0.001**	91.131	**<0.001**	0.335	83.06	**0.008**
False beliefs	16	−0.802	−1.051	−0.553	**<0.001**	68.373	**<0.001**	0.184	79.91	**<0.001**
Humor	4	−1.422	−2.268	−0.576	**<0.001**	21.893	**<0.001**	0.645	88.15	**0.001**
**Anorexia nervosa vs. HC** (All humor)	2	−1.656	−2.247	−1.064	**<0.001**	1.413	0.235	0.053	29.21	/
**FHR-B vs. HC** (All false beliefs)	2	0.171	−0.453	0.795	0.592	2.538	0.111	0.124	60.60	/
**Between disorders^a^**										
ASD vs. Early schizophrenia	2	−0.030	−0.332	0.273	0.848	0.254	0.614	0.000	0.00	**/**
ASD vs. Schizophrenia	1	0.175	−0.458	0.108	0.227	/				
Bipolar disorder vs. Depression	2	0.186	−0.207	0.580	0.353	0.017	0.896	0.000	0.00	**/**
Bipolar disorder vs. Early schizophrenia	1	0.449	−0.026	0.924	0.064	/				
Bipolar disorder vs. FHR-B	1	−0.753	−1.359	−0.147	**0.015**	/				
Bipolar disorder vs. Schizophrenia	1	0.674	−0.062	1.409	0.073	/				
BPD vs. Depression	1	−0.724	−1.195	−0.252	**0.003**	/				
CHR vs. Early schizophrenia	5	0.430	0.047	0.814	**0.028**	10.564	**0.032**	0.113	60.35	0.462
Depression vs. Early schizophrenia	2	0.295	−0.053	0.642	0.096	0.032	0.859	0.000	0.00	/
Depression vs. Schizophrenia	5	0.565	0.327	0.804	**<0.001**	1.411	0.842	0.000	0.00	0.953
Early schizophrenia vs. FHR-S	1	−0.346	−0.783	0.090	0.120	/				
FHR-S vs. Schizophrenia	2	0.168	−0.236	0.571	0.415	2.226	0.136	0.056	55.07	/

*Note*: ASD, autism spectrum disorder; HC, healthy controls; BPD, borderline personality disorder; CHR, clinical high risk for psychosis; FHR-B, familial high risk for bipolar disorder; FHR-S, familial high risk for schizophrenia; OCD, obsessive-compulsive disorder. Bold values indicate statistical significance (*p* < 0.05).

a Between disorders comparisons were conducted only when direct comparisons were available in the included studies. Positive values indicate better performance in the former condition compared to the latter condition.

Meta-regression analyses revealed that years of education (*Q*_m_ = 18.796, *p* = .049) and IQ (*Q*_m_ = 6.742, *p* = .048) positively moderated performance in ASD (Supplementary Table S4). Age negatively moderated performance in the FHR-S group (*Q*_m_ = 12.212, *p* = .04), while education (*Q*_m_ = 7.125, *p* = .015) and sample size (*Q*_m_ = 4.461, *p* = .042) showed positive associations in schizophrenia. Study quality score negatively moderated the effect size in CHR versus early schizophrenia comparisons (*Q*_m_ = 10.755, *p* = .046). Residual heterogeneity was reduced to below 75% in these groups, except for schizophrenia. Egger’s tests and funnel plots indicated potential publication bias in comparisons between schizophrenia, FHR-S, and early schizophrenia with HCs (Table 1 and Supplementary Figure S2).

### Network meta-analysis

**Figure 2.**
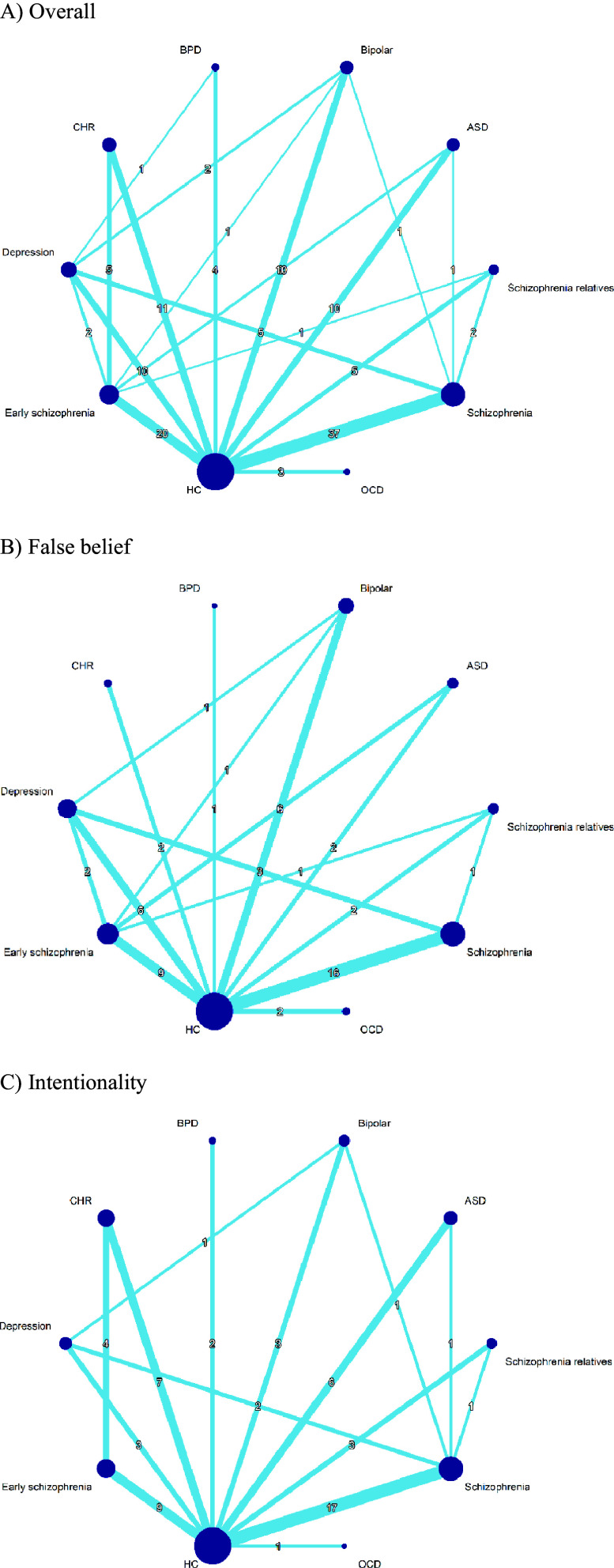
Network graph of mentalizing ability with static illustrations across conditions.

**Figure 3.**
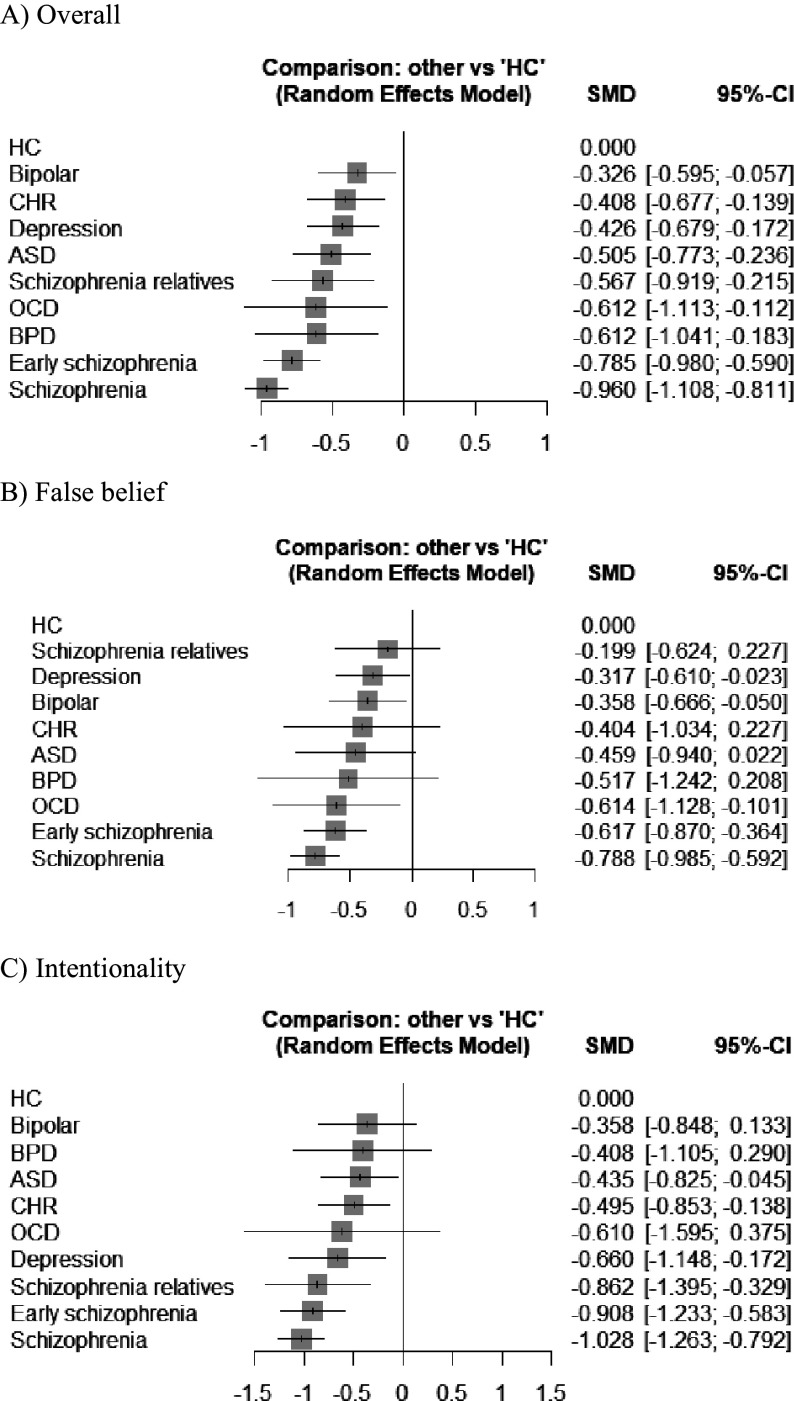
Forest plots of mentalizing ability with static illustrations across conditions.

Network graphs of overall mentalizing ability, false belief subgroup, and intentionality subgroup were illustrated in Figure 2. NMA of overall mentalizing ability with static illustrations revealed deficits across all psychiatric conditions compared to HCs (Figure 3). Effect sizes ranged from small-moderate in bipolar disorder (*g* = −0.326 [−0.596 to −0.057], *p* = 0.018), CHR (*g* = −0.408 [−0.677 to −0.139], *p* = 0.003), and depression (*g* = −0.426 [−0.679 to −0.172], *p* = 0.001); to moderate in ASD (*g* = −0.505 [−0.773 to −0.236], *p* < 0.001) and FHR-S (*g* = −0.567 [−0.919 to −0.215], *p* = 0.002); to moderate-large in OCD (*g* = −0.613 [−1.113 to −0.112], *p* = 0.017) and BPD (*g* = −0.612 [−1.041 to −0.183], *p* = 0.005); to large in early schizophrenia (*g* = −0.785 [−0.980 to −0.590], *p* < 0.001) and schizophrenia (*g* = −0.960 [−1.108 to −0.811], *p* < 0.001). Between-condition comparison revealed that both schizophrenia and early schizophrenia displayed greater impairments than most other conditions (Table 2). Notably, schizophrenia showed more pronounced impairments compared to ASD (*g* = −0.455 [−0.756 to −0.154]) and FHR-S (*g* = −0.393 [−0.761 to −0.025]), while both schizophrenia and early schizophrenia were more impaired than bipolar disorder, CHR, and depression.

**Table 2.** League table of theory of mind with static illustrations between conditions

(A) Overall									
ASD	.	.	.	.	−0.012 [−0.630; 0.605]	**−0.406 [−0.687; −0.124]**	.	0.175 [−0.630; 0.979]	.
−0.179 [−0.558; 0.200]	Bipolar	.	.	0.193 [−0.482; 0.869]	0.449 [−0.442; 1.339]	**−0.347 [−0.635; −0.059]**	.	0.674 [−0.379; 1.727]	.
0.107 [−0.399; 0.613]	0.286 [−0.219; 0.790]	BPD	.	−0.724 [−1.612; 0.165]	.	**−0.539 [−0.983; −0.094]**	.	.	.
−0.097 [−0.473; 0.280]	0.082 [−0.297; 0.461]	−0.204 [−0.710; 0.302]	CHR	.	**0.434 [0.016; 0.852]**	**−0.448 [−0.732; −0.165]**	.	.	.
−0.079 [−0.446; 0.288]	0.100 [−0.253; 0.452]	−0.186 [−0.664; 0.292]	0.018 [−0.349; 0.384]	Depression	0.297 [−0.340; 0.933]	**−0.427 [−0.716; −0.138]**	.	**0.567 [0.146; 0.988]**	.
0.280 [−0.037; 0.597]	**0.459 [0.133; 0.785]**	0.173 [−0.297; 0.643]	**0.377 [0.077; 0.677]**	**0.359 [0.052; 0.667]**	Early schizophrenia	**−0.795 [−1.001; −0.589]**	.	.	−0.346 [−1.217; 0.524]
**−0.505 [−0.773; −0.236]**	**−0.326 [−0.595; −0.057]**	**−0.612 [−1.041; −0.183]**	**−0.408 [−0.677; −0.139]**	**−0.426 [−0.679; −0.172]**	**−0.785 [−0.980; −0.590]**	HC	**0.612 [0.112; 1.113]**	**0.948 [0.794; 1.101]**	**0.453 [0.069; 0.836]**
0.108 [−0.461; 0.676]	0.286 [−0.282; 0.855]	0.001 [−0.659; 0.660]	0.204 [−0.364; 0.773]	0.187 [−0.374; 0.748]	−0.173 [−0.710; 0.365]	**0.612 [0.112; 1.113]**	OCD	.	.
**0.455 [0.154; 0.756]**	**0.634 [0.331; 0.937]**	0.348 [−0.104; 0.800]	**0.552 [0.245; 0.859]**	**0.534 [0.259; 0.809]**	0.175 [−0.068; 0.417]	**0.960 [0.811; 1.108]**	0.347 [−0.175; 0.870]	Schizophrenia	−0.124 [−0.719; 0.471]
0.062 [−0.379; 0.502]	0.241 [−0.201; 0.683]	−0.045 [−0.599; 0.510]	0.159 [−0.282; 0.599]	0.141 [−0.289; 0.571]	−0.218 [−0.610; 0.173]	**0.567 [0.215; 0.919]**	−0.046 [−0.658; 0.567]	**−0.393 [−0.761; −0.025]**	FHR-S

(B) False belief									
ASD	.	.	.	.	−0.014 [−0.550; 0.521]	−0.281 [−0.821; 0.259]	.	.	.
−0.100 [−0.666; 0.465]	Bipolar	.	.	0.169 [−0.606; 0.944]	0.449 [−0.329; 1.227]	**−0.405 [−0.724; −0.085]**	.	.	.
0.058 [−0.812; 0.928]	0.159 [−0.629; 0.946]	BPD	.	.	.	−0.517 [−1.242; 0.208]	.	.	.
−0.055 [−0.848; 0.738]	0.045 [−0.656; 0.747]	−0.113 [−1.074; 0.848]	CHR	.	.	−0.404 [−1.034; 0.227]	.	.	.
−0.142 [−0.696; 0.411]	−0.042 [−0.449; 0.366]	−0.200 [−0.982; 0.582]	−0.087 [−0.783; 0.608]	Depression	0.296 [−0.261; 0.854]	−0.321 [−0.649; 0.006]	.	**0.554 [0.107; 1.001]**	.
0.158 [−0.321; 0.638]	0.259 [−0.125; 0.643]	0.100 [−0.667; 0.868]	0.214 [−0.466; 0.893]	0.301 [−0.058; 0.659]	Early schizophrenia	**−0.630 [−0.895; −0.365]**	.	.	−0.346 [−1.101; 0.409]
−0.459 [−0.940; 0.022]	**−0.358 [−0.666; −0.050]**	−0.517 [−1.242; 0.208]	**−0.404 [−1.034; 0.227]**	**−0.317 [−0.610; −0.023]**	**−0.617 [−0.870; −0.364]**	HC	**0.614 [0.101; 1.128]**	**0.769 [0.568; 0.970]**	−0.004 [−0.489; 0.480]
0.156 [−0.548; 0.860]	0.256 [−0.343; 0.855]	0.098 [−0.791; 0.986]	0.211 [−0.602; 1.024]	0.298 [−0.294; 0.890]	−0.003 [−0.575; 0.570]	**0.614 [0.101; 1.128]**	OCD	.	.
0.330 [−0.188; 0.847]	**0.430 [0.068; 0.793]**	0.272 [−0.479; 1.023]	0.385 [−0.276; 1.045]	**0.472 [0.151; 0.792]**	0.171 [−0.142; 0.485]	**0.788 [0.592; 0.985]**	0.174 [−0.376; 0.724]	Schizophrenia	−0.301 [−0.929; 0.327]
−0.260 [−0.891; 0.371]	−0.160 [−0.682; 0.363]	−0.318 [−1.159; 0.522]	−0.205 [−0.966; 0.556]	−0.118 [−0.624; 0.388]	−0.419 [−0.885; 0.048]	**0.199 [−0.227; 0.624]**	−0.416 [−1.083; 0.251]	**−0.590 [−1.033; −0.147]**	FHR-S

(C) Intentionality.									
ASD	.	.	.	.	.	−0.348 [−0.750; 0.054]	.	0.175 [−0.710; 1.059]	.
−0.078 [−0.703; 0.547]	Bipolar	.	.	0.226 [−0.877; 1.330]	.	−0.345 [−0.907; 0.217]	.	0.674 [−0.441; 1.789]	.
−0.028 [−0.827; 0.771]	0.050 [−0.802; 0.903]	BPD	.	.	.	−0.408 [−1.105; 0.290]	.	.	.
0.060 [−0.469; 0.589]	0.138 [−0.469; 0.745]	0.087 [−0.696; 0.871]	CHR	.	0.470 [−0.038; 0.978]	**−0.552 [−0.925; −0.178]**	.	.	.
0.225 [−0.397; 0.846]	0.303 [−0.340; 0.945]	0.252 [−0.599; 1.103]	0.165 [−0.440; 0.770]	Depression	.	**−0.754 [−1.330; −0.177]**	.	0.595 [−0.186; 1.376]	.
0.473 [−0.035; 0.980]	0.550 [−0.038; 1.139]	0.500 [−0.269; 1.269]	**0.413 [0.001; 0.824]**	0.248 [−0.338; 0.834]	Early schizophrenia	**−0.928 [−1.262; −0.595]**	.	.	.
**−0.435 [−0.825; −0.045]**	−0.358 [−0.848; 0.133]	−0.408 [−1.105; 0.290]	**−0.495 [−0.853; −0.138]**	**−0.660 [−1.148; −0.172]**	**−0.908 [−1.233; −0.583]**	HC	0.610 [−0.375; 1.595]	**1.025 [0.779; 1.271]**	**0.821 [0.264; 1.378]**
0.175 [−0.885; 1.234]	0.253 [−0.848; 1.353]	0.202 [−1.004; 1.409]	0.115 [−0.933; 1.163]	−0.050 [−1.149; 1.049]	−0.298 [−1.335; 0.739]	0.610 [−0.375; 1.595]	OCD	.	.
**0.592 [0.153; 1.032]**	**0.670 [0.143; 1.198]**	0.620 [−0.116; 1.356]	**0.532 [0.104; 0.961]**	0.368 [−0.146; 0.881]	0.120 [−0.281; 0.521]	**1.028 [0.792; 1.263]**	0.417 [−0.595; 1.430]	Schizophrenia	0.151 [−0.869; 1.171]
0.427 [−0.232; 1.085]	0.504 [−0.218; 1.227]	0.454 [−0.424; 1.332]	0.367 [−0.276; 1.009]	0.202 [−0.517; 0.921]	−0.046 [−0.670; 0.579]	**0.862 [0.329; 1.395]**	0.252 [−0.868; 1.372]	−0.166 [−0.731; 0.400]	FHR-S

*Note:* Bold values indicate statistical significance (*p* < 0.05).

NMA subgroup analyses showed that early schizophrenia, schizophrenia, and depression demonstrated impairments in both false belief and intentionality tasks compared to HCs (Figure 3 and Table 2). However, bipolar disorder and OCD were significantly impaired only in false belief task performance, while ASD, CHR, and FHR-S were impaired only in intentionality tasks compared to HCs. In comparison between conditions, schizophrenia was more impaired than bipolar disorder, depression, and FHR-S in false belief tasks. For intentionality tasks, both early schizophrenia and schizophrenia displayed greater impairments than CHR. Furthermore, schizophrenia, but not early schizophrenia, demonstrated significant impairments compared to ASD and bipolar disorder. No significant differences were found between schizophrenia and early schizophrenia in overall mentalizing impairments or specific tasks.

NMA analyses demonstrated low-moderate heterogeneity for overall mentalizing ability and false belief tasks, and moderate-high heterogeneity for intentionality tasks, with minimal evidence of global and local inconsistency (Supplementary Tables S5 and S6). Potential publication biases were suggested by the comparison-adjusted funnel plot and Egger’s tests in overall mentalizing ability (*p* < 0.001) and false belief tasks (*p* = 0.042) (Supplementary Figure S3). The confidence of the evidence evaluated by CINeMA was moderate to low (Supplementary Table S7).

### Ceiling effect

Ceiling effects were observed across mentalizing tasks (Table 3 & Supplementary Table S8). The Brüne PST showed the highest rate of ceiling effects (68.3%), followed by the Langdon PST (65.7%), Yoni Task (65.0%), and Brunet/Sarfati CST (58.8%). The Happé Cartoon Task demonstrated the lowest rate of ceiling effects (33.3%). Notably, HCs consistently showed higher rates of ceiling effects (86.7–94.7%) across the first four tasks, while clinical groups showed lower rates (36.8–50.0%). Among clinical groups, CHR and ASD frequently demonstrated ceiling effects, while patients with schizophrenia rarely showed ceiling performance across all tasks. Mean performance scores of maximum possible scores followed a similar pattern, with HCs generally achieving higher scores (85.2–89.0%) compared to clinical groups (70.6–81.0%). However, it should be noted that the number of studies in many psychiatric conditions was very low (often *k* ≤ 3).

**Table 3.** Ceiling effects across mentalizing tasks and conditions

Task	Condition	No. of samples reported raw scores	No. of samples being ceiling effect	Percentage of studies being ceiling effect (%)	Mean scores (%)
Brune PST	Overall	41	28	68.3	84.7
	HC	19	18	94.7	89.0
	Clinical groups	22	10	45.5	81.0
	ASD	2	2	100	82.4
	Bipolar disorder	3	1	33.3	80.9
	BPD	1	1	100	84.4
	CHR	4	4	100	92.9
	Depression	1	0	0	78.0
	Early schizophrenia	3	2	66.6	88.0
	FHR-S	2	0	0	76.8
	Schizophrenia	6	0	0	70.5
Brunet/Sarfati CST	Overall	34	20	58.8	82.1
	HC	15	13	86.7	88.5
	Clinical groups	19	7	36.8	77.1
	ASD	3	0	0	68.7
	CHR	3	2	66.7	84.2
	Depression	1	1	100	81.1
	Early schizophrenia	4	2	50	79.9
	FHR-S	1	0	0	61.7
	Schizophrenia	7	2	28.6	77.8
Langdon PST	Overall	35	23	65.7	80.2
	HC	16	15	93.8	87.4
	Clinical groups	19	8	42.1	74.1
	ASD	1	1	100	81.9
	Bipolar disorder	1	0	0	70.6
	CHR	1	1	100	83.8
	Depression	1	1	100	83.3
	Early schizophrenia	4	2	50	77.8
	FHR-B	1	1	100	81.5
	FHR-S	1	1	100	84.0
	Schizophrenia	9	1	11.1	68.0
Yoni task	Overall	20	13	65.0	80.2
	HC	8	7	87.5	85.8
	Clinical groups	12	6	50.0	76.5
	ASD	1	1	100	83.3
	Bipolar	3	2	66.7	82.5
	Depression	1	0	0	70.2
	Early schizophrenia	3	2	66.7	75.2
	FHR-S	1	1	100	87.2
	OCD	1	0	0	73.9
	Schizophrenia	2	0	0	64.9
Happé Cartoon task	Overall	12	4	33.3	77.9
	HC	6	3	50.0	85.2
	Clinical groups	6	1	16.7	70.6
	Anorexia nervosa	2	0	0	61.1
	ASD	1	1	100	82.4
	CHR	1	0	0	77.3
	Schizophrenia	2	0	0	70.9

Abbreviations: ASD, autism spectrum disorder; BPD, borderline personality disorder; CHR, clinical high risk for psychosis; FHR-B, familial high risk for bipolar disorder; FHR-S, familial high risk for schizophrenia; OCD, obsessive-compulsive disorder; CST, comic strip task; PST, picture sequencing task.

*Note*: Ceiling effects were defined as mean performance ≥80% of maximum possible score. Mean scores are presented as percentage of maximum possible score.

## Discussion

This review represents the first comprehensive examination of mentalizing impairments assessed with static-illustration tasks across psychiatric disorders, neurodevelopmental disorders, and high-risk groups, encompassing 89 studies with 9,038 participants. Significant impairments emerged in anorexia nervosa, ASD, bipolar disorder, BPD, CHR, early schizophrenia, FHR-S, OCD, and schizophrenia, with the exception of FHR-B. Schizophrenia and early schizophrenia exhibited the largest impairments overall and in both false belief and intentionality tasks, compared to HCs and most other groups, including bipolar disorder, CHR, and depression. Domain-specific patterns were evident across conditions. NMA revealed that bipolar disorder and OCD were impaired only in false belief tasks, whereas ASD, CHR, and FHR-S were impaired only in intentionality tasks. Pairwise comparison indicated that humor comprehension deficits only appeared in ASD, anorexia nervosa, BPD, and schizophrenia. However, findings should be interpreted cautiously, given the limited studies for certain comparisons, the presence of heterogeneity, and potential publication bias. Prominent ceiling effects were observed across the commonly used tasks. This review offers valuable insights into transdiagnostic and domain-specific mentalizing impairments, guiding future research directions and the development of potential targeted interventions.

### Main discussion

Our findings revealed mentalizing impairments of varying severity across psychiatric groups, from profound deficits in schizophrenia and early schizophrenia, to moderate impairments in ASD, BPD, OCD, and FHR-S, and milder deficits in bipolar disorder, CHR, and depression. This aligns with evidence that mentalizing deficits represent a transdiagnostic feature of psychopathology [2–4]. The gradient and domain-specific profiles suggest both shared vulnerabilities and disorder-specific manifestations. These patterns may arise from bidirectional links with neural–cognitive mechanisms or symptoms across conditions [8, 30, 31]. Schizophrenia showed the most pronounced impairments compared to CHR and FHR-S, while early schizophrenia was only more impaired than CHR. This supports a progressive social cognitive decline along the schizophrenia spectrum [11, 12]. Similar impairment magnitudes in early and chronic schizophrenia suggest chronicity does not further worsen deficits, and moderate deficits may serve as endophenotypic markers [3, 32].

Although mentalizing difficulties are well-documented in ASD, our analysis found them milder than schizophrenia, contradicting previous meta-analyses [10]. One explanation may be that previous reviews pooled diverse tasks, obscuring task-specific patterns. In contrast, our review showed no false belief deficits in adults with ASD, challenging assumptions of pervasive difficulties [33]. Developmental compensation in adulthood (the mean age of our ASD samples is 23.6 years) may also account for the preserved performance [34]. This is consistent with a recent meta-analysis by Gao et al. [16], which reported only moderate deficits in perceptual scene-based abilities but greater impairments in more cognitively demanding ecological or conversational tasks in ASD compared to HCs. Furthermore, relationships between executive dysfunction and mentalizing difficulties have been shown in both ASD [35] and schizophrenia [36]. Our meta-regression analysis likewise indicated that higher education, as a proxy for cognitive reserve, attenuated impairments in both ASD and schizophrenia, highlighting the interplay between cognition and task type.

Our findings indicated small to moderate deficits on static-illustration tasks in both bipolar disorder and depression. Whether these are state- or trait-linked remains unresolved [2, 32]. Associations with mood symptoms were inconsistent [37–39], while evidence for a psychosis contribution was limited [40, 41]. In contrast, poorer neurocognition or lower intelligence consistently predicts weaker mentalizing [37, 42, 43]. This is indeed consistent with findings that many individuals with bipolar disorders (about half to two-thirds) retain intact social cognition when neurocognitive functions are preserved [44, 45].

Beyond cognition and mood symptoms, demographic and clinical factors also contributed to impairment severity. Longer illness duration, poorer insight, and male sex were linked to poorer social cognition [44, 46, 47]. Neuroimaging studies further indicate that age, biological sex, and individual social cognitive performances, rather than diagnostic categories per se, explain much of the variance in mentalizing-related networks [31, 48, 49]. Such interindividual variability likely underlies the heterogeneity observed in our meta-analyses and underscores the need for transdiagnostic, multidimensional frameworks that account for personal and clinical factors alongside traditional diagnoses.

Our subgroup analyses further revealed distinct domain-specific patterns of mentalizing impairments across conditions, suggesting differential profiles of social cognitive dysfunction. Schizophrenia, early schizophrenia, and depression exhibited deficits in both false belief and intentionality tasks, indicating a generalized impairment. In contrast, bipolar disorder and OCD displayed selective impairments in false belief tasks, a relatively straightforward construct involving basic perspective taking and object permanence, while maintaining intact intentionality comprehension. This suggested that individuals with bipolar disorder and OCD may preserve the ability to infer intentions but struggle with self-other differentiation, potentially relying on their own mental states to infer others’ beliefs [15, 22]. However, only two OCD studies assessed false beliefs, and heterogeneity and potential publication bias affected bipolar disorder analyses, warranting cautious interpretations. Moreover, false belief tasks vary in operationalization, targeting different aspects of belief reasoning, which likely contributes to inconsistent findings (Supplementary Table S3). Notably, OCD patients demonstrated impairments in affective but not cognitive second-order beliefs [50], whereas bipolar disorder patients were impaired only in cognitive second-order beliefs, with intact affective second-order and first-order performance [38].

Intentionality tasks, the most widely studied domain, revealed further divergence. ASD, CHR, and FHR-S showed specific difficulty in inferring intentions despite intact self-other differentiation. Our analyses addressed impairment magnitude but not direction. Theoretical models distinguish “hypomentalizing,” reduced attribution of mental states, from “hypermentalizing,” excessive, overly elaborate attributions, along the proposed “autism-psychosis continuum” [51, 52]. Indeed, evidence indicates that schizophrenia, early schizophrenia, bipolar disorder, and CHR groups often exhibit hypermentalizing biases during gaze perception [53–56]. A meta-analysis by McLaren et al. [5] further suggests that hypermentalizing is a transdiagnostic feature across multiple disorders, not limited to BPD or schizophrenia, particularly on complex video-based tasks such as the Movie for the Assessment of Social Cognition. In BPD, attachment hyperactivation may underlie hypermentalizing in emotionally charged contexts. However, little is known about how these biases shift across contexts, such as low emotional arousal or third-person perspectives. Current static-illustration tasks cannot capture bias direction and may obscure subtle distinctions in how individuals infer others’ minds by heavily relying on standardized stimuli with correct/incorrect scoring. Future research could employ tasks capable of differentiating over- versus under-ascription of mental states [57–59], offering deeper insights into hyper- and hypomentalizing across disorders.

For humor comprehension, significant impairments were found only in ASD, anorexia nervosa, BPD, and schizophrenia. These deficits likely reflect difficulties in processing non-literal meaning, interpreting subtle social cues, and managing complex social perceptions [60, 61], signaling broader challenges in understanding social nuances essential for communication and social functioning. Given the limited number of studies, however, these findings should be interpreted cautiously.

Overall, our results reveal domain-specific patterns across false belief, intentionality, and humor tasks, reinforcing that mentalizing is not a unitary construct but a constellation of partially independent processes. Future work should disentangle specific components, such as affective and order components, to clarify distinct neural and clinical mechanisms. Beyond theory, the current synthesis offers empirical insights and directions for developing targeted interventions across diagnostic groups that can leverage preserved abilities while addressing areas of impairment. Therapeutic approaches, from traditional social cognition remediation [62], noninvasive brain stimulation [63], to emerging virtual-reality-based interventions [64], may differentially target mentalizing domains and enhance functional outcomes across psychiatric disorders.

### Methodological considerations

This review differs from prior work by focusing on mentalizing tasks that employ static illustrations or comic strips, thereby reducing heterogeneity and allowing more consistent cross-study comparisons, a key prerequisite for meaningful meta-analysis [65]. Despite these advantages, several methodological limitations warrant attention. Prominent ceiling effects were observed, especially in HC, ASD, CHR, and bipolar disorder groups, which may obscure milder impairments by compressing scores near the upper limit [17, 18, 66]. Consequently, subtle deficits may appear absent unless more sensitive measures are used.

Ecological validity presents another significant concern. Recent reviews have questioned whether these tasks truly measure mentalizing ability rather than specific problem-solving skills [22, 66]. The laboratory–life gap risks misrepresenting social cognition in both clinical and general populations. Moreover, the relationship between mentalizing performance and real-world functioning remains underexplored, with only a few studies reporting mixed findings [67]. In response, more ecologically valid approaches have been developed, such as video-based paradigms [68, 69] and interactive virtual reality assessments [70, 71], which can better simulate dynamic social contexts. With advances in natural language processing and large language models (LLMs), dynamic and adaptive mentalizing assessments and interventions have become increasingly feasible. These approaches enable modeling of mentalizing processes at both computational and semantic levels [72, 73] and allow personalized, real-time adaptations based on participants’ inferred mental states and interactive behaviors [74, 75]. Future research should explore the integration of dynamic, context-rich, and potentially LLM-enhanced paradigms to advance the modeling and understanding of mentalizing ability, complement existing tools, and inform the development of more effective and ecologically valid interventions.

## Limitations

First, despite the large overall sample size, certain groups (e.g., anorexia nervosa and FHR-B) were underrepresented, limiting generalizability and precluding inclusion in the NMA. Similarly, some pairwise meta-analyses were based on only two or three studies per condition, which may yield imprecise or biased estimates. We retained these analyses to provide a comprehensive overview of all available literature, but the corresponding results should be interpreted with caution due to limited statistical power and potential bias. Second, substantial heterogeneity was observed, particularly in HC comparisons with ASD, bipolar disorder, FHR-S, and schizophrenia, as well as in the NMA for intentionality tasks. This likely reflected methodological, sample, and task differences. Publication bias was evident in several pairwise contrasts, and effect sizes were moderated by sample size and study quality. Third, comorbidity was inadequately reported despite its high prevalence [76, 77], and evolving diagnostic criteria may also have influenced results [78]. Fourth, our focus on static illustration tasks reduced task heterogeneity and enabled meaningful synthesis but may limit generalization to other modalities. Future reviews should extend to other measures of mentalizing and social cognition, such as dynamic video-based tasks and naturalistic social scenarios. Fifth, this review focused exclusively on psychiatric populations, and findings may not generalize to other groups with social cognitive deficits (e.g., brain injury, Huntington’s disease, and Parkinson’s disease). Finally, restricting inclusion to English, peer-reviewed articles may have introduced language and publication bias.

## Conclusion

This meta-analysis revealed significant mentalizing impairments, assessed through static illustration tasks, across psychiatric disorders, neurodevelopmental disorders, and at-risk groups. Notably, schizophrenia and early schizophrenia demonstrated the most pronounced deficits overall and across domains. While impairments were transdiagnostic, distinct domain-specific patterns (false belief, intentionality, and humor) suggested both shared vulnerabilities and disorder-specific mechanisms. Moreover, the substantial heterogeneity points to the importance of interindividual variability, which may complement diagnostic categories in identifying mechanistic pathways. At the same time, methodological challenges, such as ceiling effects and limited ecological validity, highlight the need for more refined and ecologically relevant measures. Future studies should prioritize domain-specific mechanisms within a transdiagnostic framework to advance both theory and intervention design.

## Supporting information

10.1192/j.eurpsy.2025.10146.sm001 Tsui et al. supplementary material Tsui et al. supplementary material

## Data Availability

The search strategy is detailed in the Supplementary Material. Full search results and data entry forms are available from the authors upon request.

## Supplementary material

The supplementary material for this article can be found at http://doi.org/10.1192/j.eurpsy.2025.10146.
