# Supplementary material for: Mentalizing impairments across 11 psychiatric conditions: A transdiagnostic systematic review and network meta-analysis of tasks with static illustrations
Source: Eur Psychiatry. 2025 Dec 23;69(1):e2. doi: 10.1192/j.eurpsy.2025.10146 (PMC12816936; doi:10.1192/j.eurpsy.2025.10146)
Supplement: Tsui et al. supplementary material [file S0924933825101466sup001.docx]

Supplementary material for

**Mentalizing Impairments Across Psychiatric Disorders: A Transdiagnostic Systematic Review and Network Meta-Analysis of Tasks with Static Illustrations**

[Supplementary Methods. PRISMA Checklists 2](#_Toc206505005)

[Supplementary Methods. Inclusion and exclusion criteria, and key words. 4](#_Toc206505006)

[Supplementary Methods. Modified Newcastle-Ottawa Scale for Quality Assessment 4](#_Toc206505007)

[Supplementary Methods. Effect size calculation 6](#_Toc206505008)

[Supplementary Methods. Assessment of network heterogeneity and inconsistency 7](#_Toc206505009)

[Supplementary Discussion. Domain-Specific Patterns and Mentalizing Direction 8](#_Toc206505010)

[Supplementary Table 1. Characteristics of included studies 9](#_Toc206505011)

[Supplementary Table 2. Characteristics of included studies by conditions 25](#_Toc206505012)

[Supplementary Table 3. Characteristics of included mentalizing tasks 26](#_Toc206505013)

[Supplementary Table 4. Meta-regression of theory of mind with static illustrations across psychiatric conditions compared to healthy controls, and between conditions. 29](#_Toc206505014)

[Supplementary Table 5. Network heterogeneity and inconsistency 31](#_Toc206505015)

[Supplementary Table 6. Comparison of direct and indirect evidence using side (separating indirect from direct evidence) analysis 32](#_Toc206505016)

[Supplementary Table 7. Confidence in the evidence evaluated using the Confidence in Network Meta-Analysis (CINeMA) framework. 34](#_Toc206505017)

[Supplementary Table 8. Ceiling effects across mentalizing tasks and conditions 37](#_Toc206505018)

[Supplementary Figure 1. Forest plots of theory of mind with static illustrations across psychiatric conditions compared to healthy controls, and between conditions. 38](#_Toc206505019)

[Supplementary Figure 2. Funnel plots of theory of mind with static illustrations across psychiatric conditions compared to healthy controls, and between conditions. 45](#_Toc206505020)

[Supplementary Figure 3. Comparison-adjusted funnel plots and publication bias assessment for network meta-analysis 49](#_Toc206505021)

# Supplementary Methods. PRISMA Checklists

| **Section and Topic** | **Item #** | **Checklist item** | **Pages where item is reported** |
| --- | --- | --- | --- |
| **TITLE** | | |  |
| Title | 1 | Identify the report as a systematic review. | 1 |
| **ABSTRACT** | | |  |
| Abstract | 2 | See the PRISMA 2020 for Abstracts checklist. | 2 |
| **INTRODUCTION** | | |  |
| Rationale | 3 | Describe the rationale for the review in the context of existing knowledge. | 3-4 |
| Objectives | 4 | Provide an explicit statement of the objective(s) or question(s) the review addresses. | 4 |
| **METHODS** | | |  |
| Eligibility criteria | 5 | Specify the inclusion and exclusion criteria for the review and how studies were grouped for the syntheses. | 5 |
| Information sources | 6 | Specify all databases, registers, websites, organisations, reference lists and other sources searched or consulted to identify studies. Specify the date when each source was last searched or consulted. | 5 |
| Search strategy | 7 | Present the full search strategies for all databases, registers and websites, including any filters and limits used. | 5 |
| Selection process | 8 | Specify the methods used to decide whether a study met the inclusion criteria of the review, including how many reviewers screened each record and each report retrieved, whether they worked independently, and if applicable, details of automation tools used in the process. | 5-6 |
| Data collection process | 9 | Specify the methods used to collect data from reports, including how many reviewers collected data from each report, whether they worked independently, any processes for obtaining or confirming data from study investigators, and if applicable, details of automation tools used in the process. | 5-6 |
| Data items | 10a | List and define all outcomes for which data were sought. Specify whether all results that were compatible with each outcome domain in each study were sought (e.g. for all measures, time points, analyses), and if not, the methods used to decide which results to collect. | 5-6 |
|  | 10b | List and define all other variables for which data were sought (e.g. participant and intervention characteristics, funding sources). Describe any assumptions made about any missing or unclear information. | 5-6 |
| Study risk of bias assessment | 11 | Specify the methods used to assess risk of bias in the included studies, including details of the tool(s) used, how many reviewers assessed each study and whether they worked independently, and if applicable, details of automation tools used in the process. | 6, Supplementary Methods |
| Effect measures | 12 | Specify for each outcome the effect measure(s) (e.g. risk ratio, mean difference) used in the synthesis or presentation of results. | 6-7, Supplementary Methods |
| Synthesis methods | 13a | Describe the processes used to decide which studies were eligible for each synthesis (e.g. tabulating the study intervention characteristics and comparing against the planned groups for each synthesis (item #5)). | 6-7 |
|  | 13b | Describe any methods required to prepare the data for presentation or synthesis, such as handling of missing summary statistics, or data conversions. | 6-7 |
|  | 13c | Describe any methods used to tabulate or visually display results of individual studies and syntheses. | 6-7 |
|  | 13d | Describe any methods used to synthesize results and provide a rationale for the choice(s). If meta-analysis was performed, describe the model(s), method(s) to identify the presence and extent of statistical heterogeneity, and software package(s) used. | 6-7 |
|  | 13e | Describe any methods used to explore possible causes of heterogeneity among study results (e.g. subgroup analysis, meta-regression). | 6-7 |
|  | 13f | Describe any sensitivity analyses conducted to assess robustness of the synthesized results. | 6-7 |
| Reporting bias assessment | 14 | Describe any methods used to assess risk of bias due to missing results in a synthesis (arising from reporting biases). | 6-7 |
| Certainty assessment | 15 | Describe any methods used to assess certainty (or confidence) in the body of evidence for an outcome. | 6-7 |
| **RESULTS** | | |  |
| Study selection | 16a | Describe the results of the search and selection process, from the number of records identified in the search to the number of studies included in the review, ideally using a flow diagram. | 7, Figure 1 |
|  | 16b | Cite studies that might appear to meet the inclusion criteria, but which were excluded, and explain why they were excluded. | N/A |
| Study characteristics | 17 | Cite each included study and present its characteristics. | Supplementary Table 1, Supplementary Table 2 |
| Risk of bias in studies | 18 | Present assessments of risk of bias for each included study. | 8, Supplementary Table 1 |
| Results of individual studies | 19 | For all outcomes, present, for each study: (a) summary statistics for each group (where appropriate) and (b) an effect estimate and its precision (e.g. confidence/credible interval), ideally using structured tables or plots. | Table 1, Supplementary Figure 1 |
| Results of syntheses | 20a | For each synthesis, briefly summarise the characteristics and risk of bias among contributing studies. | 7-9, Table 1, Table 2, Figure 2, Figure 3, Supplementary Table 5 |
|  | 20b | Present results of all statistical syntheses conducted. If meta-analysis was done, present for each the summary estimate and its precision (e.g. confidence/credible interval) and measures of statistical heterogeneity. If comparing groups, describe the direction of the effect. | 7-9, Table 1, Table 2, Figure 2, Figure 3, Supplementary Table 5, Supplementary Table 6 |
|  | 20c | Present results of all investigations of possible causes of heterogeneity among study results. | 7-10, Table 3, Supplementary Table 4 |
|  | 20d | Present results of all sensitivity analyses conducted to assess the robustness of the synthesized results. | Supplementary Table 4 |
| Reporting biases | 21 | Present assessments of risk of bias due to missing results (arising from reporting biases) for each synthesis assessed. | Table 1, Supplementary Figure 2, Supplementary Figure 3 |
| Certainty of evidence | 22 | Present assessments of certainty (or confidence) in the body of evidence for each outcome assessed. | 9, Supplementary Table 7 |
| **DISCUSSION** | | |  |
| Discussion | 23a | Provide a general interpretation of the results in the context of other evidence. | 10-16 |
|  | 23b | Discuss any limitations of the evidence included in the review. | 15-16 |
|  | 23c | Discuss any limitations of the review processes used. | 16 |
|  | 23d | Discuss implications of the results for practice, policy, and future research. | 10-17 |
| **OTHER INFORMATION** | | |  |
| Registration and protocol | 24a | Provide registration information for the review, including register name and registration number, or state that the review was not registered. | 5 |
|  | 24b | Indicate where the review protocol can be accessed, or state that a protocol was not prepared. | N/A |
|  | 24c | Describe and explain any amendments to information provided at registration or in the protocol. | N/A |
| Support | 25 | Describe sources of financial or non-financial support for the review, and the role of the funders or sponsors in the review. | 18 |
| Competing interests | 26 | Declare any competing interests of review authors. | 18 |
| Availability of data, code and other materials | 27 | Report which of the following are publicly available and where they can be found: template data collection forms; data extracted from included studies; data used for all analyses; analytic code; any other materials used in the review. | 18 |

# Supplementary Methods. Inclusion and exclusion criteria, and search strategy details.

| **Inclusion Criteria** | **Rationale / Notes** |
| --- | --- |
| 1. Examined psychiatric disorders, neurodevelopmental disorders, or high-risk populations. | Ensures focus on target clinical groups relevant to the review objectives. |
| 2. Employed at least one mentalizing task using static illustrations. | Maintains methodological homogeneity to enable valid cross-study comparisons. |
| 3. Included comparison groups of healthy controls or other targeted populations, assessed with validated diagnostic tools (e.g., DSM, ICD). | Ensures diagnostic validity and comparability between groups. |
| 4. Reported sufficient statistics for effect size calculations (e.g., means, standard deviations, t-statistics, F-statistics, p-values). | Required for quantitative synthesis and meta-analysis. |
| 5. Published in peer-reviewed English-language journals. | Ensures methodological quality and accessibility of full methodological details. |
| **Exclusion Criteria** | **Rationale / Notes** |
| 1. Involved paediatric or early adolescent populations (<15 years). | Excluded due to developmental differences in mentalizing abilities, as performance in younger populations may reflect ongoing maturation rather than disorder-specific impairments, introducing confounds in cross-condition comparisons. |
| 2. Included participants with intellectual disability. | ID is associated with global cognitive impairments that may confound interpretation of disorder-specific mentalizing deficits. |
| 3. Included only mixed or comorbid clinical groups without separate reporting for the target disorder. | Prevents contamination of results from comorbid conditions, allowing clearer attribution of impairments to the primary disorder of interest. |
| 4. Included duplicate samples. | Avoids inflating sample size and biasing effect estimates. |
| 5. Publication type: abstracts, conference posters, qualitative studies, reviews, or case reports. | These formats typically lack sufficient methodological detail or statistical data for meta-analysis. |

# In addition to database searches, we manually screened the reference lists of all included studies and relevant systematic reviews to identify additional eligible publications. Forward citation tracking was also conducted in Google Scholar to capture more recent studies citing key articles. All records were managed using Covidence, which automatically removed duplicates and facilitated title/abstract and full-text screening.

# Supplementary Methods. Modified Newcastle-Ottawa Scale for Quality Assessment

The quality of the included studies was assessed using a modified version of the Newcastle-Ottawa Scale (NOS). The scale evaluates studies based on several key criteria related to study design, participant selection, and outcome reporting. Each criterion was awarded points, with a maximum possible score indicating the study’s overall methodological quality.

1. **Case Assessment**
   - **1 point** if a structured clinical interview was used to confirm the diagnosis.
2. **Case Definition**
   - **1 point** if patients were diagnosed according to DSM/ICD criteria.
3. **Control Definition**
   - **1 point** if controls were free of psychiatric disease.
   - If no controls were used, **1 point** was awarded if all diagnostic groups were diagnosed according to DSM/ICD criteria.
4. **Representativeness of Cases**
   - **1 point** if a consecutive or clearly representative series of cases was used.
   - **0 points** if there was no description of the recruitment strategy or if participants were recruited from a single specialist service.
5. **Comparability of Cases and Controls**
   - **1 point** if cases and controls were matched for age.
   - **1 point** if cases and controls were matched for sex.
6. **Representativeness of Controls**
   - **1 point** if controls were taken from the same local community as the patients.
   - If no controls were used, this criterion was assessed based on whether various patient groups were recruited from the same community.
7. **Ascertainment of Exposure**
   - **1 point** if validated theory of mind tasks were used to assess mentalizing abilities.
8. **Outcome Reporting**
   - **1 point** if raw descriptive statistics were provided for the outcomes of interest.

# Supplementary Methods. Effect size calculation

Effect sizes were calculated to summarize the magnitude of mentalizing impairments across studies. The following steps were taken to ensure consistency and accuracy in effect size estimation:

1. **Multiple Tasks and Effect Sizes**
   When a study reported multiple tasks measuring mentalizing, each task was treated as a separate effect size. This approach allowed us to capture the full range of tasks used in mentalizing assessments across studies and to provide a more granular comparison between conditions. By treating each task individually, we were able to better account for domain-specific variations in performance, which is important given the heterogeneity in task designs and cognitive domains involved.
2. **Multiple Scoring Conditions Within Tasks**
   In cases where a task had multiple scoring conditions (e.g., cognitive, affective, first-order, and second-order components), we used a combined average score for the task. While these different components may represent distinct cognitive or emotional constructs, there was insufficient data to separately examine these nuanced constructs across studies. Averaging across conditions was done to maintain consistency with other tasks and to reduce complexity in the meta-analysis. This approach allowed us to provide a more generalized estimate of overall mentalizing performance across different conditions within the same task, which was important for ensuring homogeneity in the analysis.
3. **Affective versus Cognitive Components**
   We attempted to examine the affective versus cognitive components of mentalizing, as these components are often distinguished in theoretical models of mentalizing and social cognition. However, only four tasks—Brüne novel CST, Gallagher cartoon, Schnell cartoon, and Yoni Task—contained affective components in 13 studies across 10 conditions. Due to limited studies and the lack of sufficient variation in these components across conditions, these tasks were excluded from the meta-analysis.
4. **First-Order versus Second-Order Mentalizing**
   Similarly, we attempted to examine the first-order versus second-order mentalizing components, given that these represent different levels of social cognitive processing. Specifically, second-order false belief tasks (FBT) require participants to infer others’ beliefs about a third party, which is considered a more complex cognitive task than first-order tasks. Four tasks—Cartoon FBT, Moore FBT, Yoni Task, and FB—examined second-order false beliefs across 12 studies in 8 conditions. However, due to the limited number of studies that exclusively examined second-order false beliefs, these tasks were not included in the meta-analysis, as they did not meet the criteria for a sufficiently large sample size.

By carefully considering the task characteristics and component distinctions, we aimed to create a balance between capturing key constructs of mentalizing and maintaining sufficient homogeneity for reliable meta-analytic comparisons. Excluding tasks with insufficient data for meaningful analysis ensures the robustness of the findings while enhancing the overall consistency of the meta-analysis. Future research should separately examine the sub-components of mentalizing, as they may reflect distinct processes. Greater focus is needed on differentiating between these components, with careful consideration of how the constructs are operationalized, what they represent, and how they are measured. Expanding the inclusion of these tasks in meta-analyses, with larger and more diverse samples, would strengthen findings and inform clinical applications.

# Supplementary Methods. Assessment of network heterogeneity and inconsistency

For the assessment of heterogeneity in Theory of Mind performance across psychiatric disorders, we evaluated network statistical heterogeneity by comparing the estimated tau (τ) for between-study heterogeneity to empirical distributions provided by Rhodes et al. (2015) for mental health outcomes 0.15 (0.07, 0.29).

Heterogeneity was classified as: Low: below the 25% quantile; Low-moderate: between 25% and 50% quantile; Moderate-high: between 50% and 75% quantile; High: above the 75% quantile.

For the entire network, we based our assessment on the magnitude of the heterogeneity variance parameter (τ²) estimated from the NMA models. We also calculated a total I² value to quantify heterogeneity in the network.

For assessment of inconsistency, we evaluated inconsistency using two approaches:
a) Global consistency: Assessed under the assumption of a full design-by-treatment interaction model, using the 'decompose.design' function in the R package 'netmeta'.
b) Local consistency: Evaluated using the SIDE (Separating Indirect from Direct Evidence) test.

These methods allowed us to identify potential sources of heterogeneity across different psychiatric conditions and task types (Intentionality and False Belief), which could indicate systematic differences in Theory of Mind impairments.

# Supplementary Discussion. Domain-Specific Patterns and Mentalizing Direction

**False-Belief Deficits in Bipolar Disorder and OCD**

This suggested that individuals with bipolar disorder and OCD may have a preserved ability to understand others' intentions and motivations but struggle with self-other differentiation, potentially relying on their own mental states to infer others' beliefs, leading to difficulties in distinguishing between self and other perspectives (Eddy, 2019; Quesque & Rossetti, 2020). However, it is important to note that only two studies on OCD examining false beliefs were available, and significant heterogeneity and potential publication bias were observed in the analyses for bipolar disorder, warranting cautious interpretation of these findings. Although the tasks in the domain of false belief were all aim to measure the ability to understand others' beliefs or perspectives that are different from one's own, they differ in their operationalizations and the specific aspects they target, which likely contributes to variations in experimental designs and the associated cognitive challenges (Supplementary Table 3). Notably, individuals with OCD demonstrated impairments in affective second-order beliefs but not in cognitive second-order beliefs (Liu et al., 2017), whereas bipolar disorder was significantly impaired only in cognitive second-order beliefs, with intact performance in affective second-order and first-order false belief tasks (Wang et al., 2018).

**Hyper- vs. Hypomentalizing in Intentionality Tasks**

A meta-analysis by McLaren et al. (2022) suggests that hypermentalizing may be a transdiagnostic feature presence across multiple psychiatric disorders rather than disorder-specific for BPD or schizophrenia, based on the complex video-based task, Movie for the Assessment of Social Cognition. In BPD, attachment hyperactivation may contribute to hypermentalizing in emotionally charged contexts (McLaren et al., 2022). However, less is known about how these biases might shift under different social scenarios, such as low emotional arousal or third-person perspectives. The existing tasks with static illustrations failed to capture the direction of potential biases and thus unable to discern the hypermentalizing and hypomentalizing tendencies among psychiatric disorders. The reliance on standardized stimuli and correct versus incorrect response formats may obscure subtler distinctions in how individuals form or overextend inferences about others’ minds. Future research could incorporate tasks designed to detect whether errors stem from over- or under-ascription of mental states (Chan et al., 2021; Fretland et al., 2015; Peyroux et al., 2019; Wastler et al., 2019), thereby offering deeper insights into how hyper- and hypomentalizing manifest in different clinical groups.

# Supplementary Table 1. Characteristics of included studies

| **no.** | **Author, publication year** | **Country** | **Population** | **N** | **Sex (no. of female)** | **Age Mean** | **Years of education** | **IQ Mean** | **Diagnostic tools** | **Comorbidity details (exclusion)** | **Medication details** | **ToM tasks** |
| --- | --- | --- | --- | --- | --- | --- | --- | --- | --- | --- | --- | --- |
| 1 | Achim et al., 2011 | Canada | Healthy Controls | 31 | 5 | 25.2 ± 4.2 | NR | 101.8 | DSM-IV | / | / | Sarfati CST |
| 1 | Achim et al., 2011 | Canada | FEP | 31 | 5 | 24.9 ± 4.5 | NR | 100.4 | DSM-IV | Did not exclude alcohol/ substance abuse, comorbid Axis I symptoms or diagnoses | Quetiapine 54%/ 770.6mg Olanzapine 12%/ 15mg Risperidone 6%/ 2.3mg Risperidone Consta 9%/ 25mg Combination 12% | Sarfati CST |
| 2 | Achim et al., 2023 | Canada | Healthy Controls | 22 | 6 | 25.6 ± 5.9 | NR | 105.8 | NR | / | / | Sarfati CST |
| 2 | Achim et al., 2023 | Canada | FEP | 25 | 3 | 27.1 ± 4.8 | NR | 98 | NR | NR | Aripiprazole (40%) Olanzapine (40%) Quetiapine XR (20%) Clozapine (12%) Risperidone consta (8%) Risperidone (8%) Paliperidone palmitate (4%) Aripiprazole and quetiapine (4%) | Sarfati CST |
| 3 | Anselmetti et al., 2009 | Italy | Healthy Controls | 47 | 22 | 29.8 ± 5.2 | 15.1 ± 2.9 | NR | DSM-IV | / | / | Brüne PST |
| 3 | Anselmetti et al., 2009 | Italy | Healthy Controls (father) | 47 | 0 | 54.6 ± 11.6 | 12.3 ± 4.1 | NR | NR | / | / | Brüne PST |
| 3 | Anselmetti et al., 2009 | Italy | Healthy Controls (mother) | 47 | 47 | 55.1 ± 8.2 | 11.6 ± 4 | NR | NR | / | / | Brüne PST |
| 3 | Anselmetti et al., 2009 | Italy | Schizophrenia | 47 | 16 | 32.1 ± 7.8 | 11.4 ± 2.9 | 103.7 | DSM-IV | NR | Clozapine (53%): 250 mg Risperidone (27%): 4 mg Aripiprazole (10%): 15 mg Haloperidol (8.5%): 4 mg | Brüne PST |
| 3 | Anselmetti et al., 2009 | Italy | First-degree relatives (father) | 47 | 0 | 62.4 ± 8.3 | 10.1 ± 4.2 | 109.2 | NR | NR | NR | Brüne PST |
| 3 | Anselmetti et al., 2009 | Italy | First-degree relatives (mother) | 47 | 47 | 60.2 ± 8.6 | 9.1 ± 3.7 | 108.7 | NR | Excluded alcohol/substance abuse, comorbid diagnosis on Axis II, epilepsy or any other major neurological illness or perinatal trauma | NR | Brüne PST |
| 4 | Atkinson et al., 2017 | Australia | Healthy Controls | 61 | 29 | 19.1 ± 3.19 | 11.9 ± 2.62 | 118 | DSM-IV | / | / | Langdon PST |
| 4 | Atkinson et al., 2017 | Australia | CHR | 102 | 55 | 18.6 ± 2.71 | 10 ± 2.54 | 104 (WASI 2 Subset) | DSM-IV | Excluded pre-existing psychosis with symptoms exceeding the CAARMS psychosis threshold, antipsychotic pharmacotherapy, diagnosis of drug abuse or dependence | NR | Langdon PST |
| 5 | Bartholomeusz et al., 2018 | Australia | Healthy Controls | 22 | 13 | 20.41 ± 2.59 | 13.59 ± 1.92 | NR | DSM-IV | / | / | Langdon PST |
| 5 | Bartholomeusz et al., 2018 | Australia | FEP | 14 | 6 | 20.43 ± 3.35 | 12.29 ± 1.49 | NR | DSM-IV | Did not exclude comorbidities | Atypical Antipsychotic Medication (85%) Antidepressant & Anxiolytic Medication (42%) Only Antidepressant (14%) | Langdon PST |
| 6 | Bazin et al., 2009 | France | Healthy Controls | 15 | 10 | 36.1 ± 17 | NR | NR | DSM-IV | / | / | Sarfati CST |
| 6 | Bazin et al., 2009 | France | Bipolar disorder | 15 | 2 | 45.49 ± 14.63 | 13.9 ± 3 | NR | DSM-IV | NR | NR | Sarfati CST |
| 6 | Bazin et al., 2009 | France | Depression | 15 | 6 | 46.7 ± 18.1 | 13.4 ± 3 | NR | DSM-IV | NR | NR | Sarfati CST |
| 6 | Bazin et al., 2009 | France | Schizophrenia | 15 | 3 | 35.4 ± 9.6 | 11.3 ± 2.6 | NR | DSM-IV | NR | NR | Sarfati CST |
| 7 | Bechi et al., 2018 | Italy | Healthy Controls | 67 | 31 | 20.32 ± 3.06 | 13.29 ± 4.13 | NR | DSM IV-R | / | / | Brüne PST |
| 7 | Bechi et al., 2018 | Italy | Schizophrenia | 122 | 44 | 44.93 ± 11.05 | 11.637 ± 2.738 | 86.52 | DSM IV-R | NR | NR | Brüne PST |
| 8 | Bednarz et al., 2021 | US | Healthy Controls | 18 | 7 | 39.03 ± 13.96 | NR | 103.28 | AQ | / | / | Brüne PST |
| 8 | Bednarz et al., 2021 | US | ASD | 13 | 6 | 19.77 ± 2.22 | NR | 109.08 | AQ | anxiety, asthma, depression, GERD, irritable bowel syndrome, raynaud's disease sleep apnea | SSRI (7%), SNRI (7%), NDRI (7%), anxiolytic (7%) | Brüne PST |
| 9 | Bentall et al., 2009 | UK | Healthy Controls | 33 | 19 | 31.88 ± 11.54 | NR | 108.64 | NR | / | / | Moore FBT |
| 9 | Bentall et al., 2009 | UK | Depression | 27 | 18 | 48.37 ± 10.97 | NR | 108.2 | NR | n=20 (SZ & Depression) | NR | Moore FBT |
| 9 | Bentall et al., 2009 | UK | Schizophrenia | 39 | 13 | 33.95 ± 8.38 | NR | 92.18 | NR | n=20 (SZ & Depression) | NR | Moore FBT |
| 10 | Berry et al., 2015 | UK | Healthy Controls | 25 | 8 | 37.4 ± 8.32 | NR | 103.91 | NR | / | / | Frith FBT |
| 10 | Berry et al., 2015 | UK | Schizophrenia | 25 | 8 | 32.32 ± 9.25 | NR | 101.77 | NR | NR | Typical (68%), atypical (24%), typical & atypical (8%) | Frith FBT |
| 11 | Bozikas et al., 2011 | Greece | Healthy Controls | 30 | 6 | 28.8 ± 4.1 | 12.33 ± 2.28 | NR | DSM-IV | / | / | FB cartoon |
| 11 | Bozikas et al., 2011 | Greece | Schizophrenia | 36 | 6 | 36.72 ± 7.8 | 12.19 ± 2.58 | NR | DSM-IV | Excluded a history of neurological or developmental disorders, recent substance abuse (in the last six months), as well as a co-morbid psychiatric disorder, or a medical disorder which might compromise cognitive performance | atypical antipsychotics (75%),  combination of two atypical antipsychotics (16%),  typical antipsychotics (5%) and combination of atypical and typical antipsychotics (2%). | FB cartoon |
| 12 | Brüne et al., 2011 | Germany | Healthy Controls | 26 | 9 | 25.3 ± 7.7 | NR | NR | DSM-IV | / | / | Brüne PST |
| 12 | Brüne et al., 2011 | Germany | CHR | 10 | 3 | 25.5 ± 5.3 | NR | NR | DSM-IV | Excluded substance dependence, traumatic brain injury or mental retardation | NR | Brüne PST |
| 12 | Brüne et al., 2011 | Germany | FEP | 22 | 7 | 26.8 ± 5.5 | NR | NR | DSM-IV | Excluded substance dependence, traumatic brain injury or mental retardation | NR | Brüne PST |
| 13 | Brüne et al., 2016 | Germany | Healthy Controls | 30 | 30 | 35.5 ± 12.8 | NR | NR | DSM-V | / | / | Brüne novel CST |
| 13 | Brüne et al., 2016 | Germany | BPD | 30 | 30 | 25.7 ± 6.3 | NR | NR | DSM-V | Depressive episode | Antidepressants including agomelatine, citalopram, and bupropion (63.3%) | Brüne novel CST |
| 14 | Brune, 2005 | Germany | Healthy Controls | 18 | 10 | 34.2 ± 10.3 | NR | 105 | DSM-IV | / | / | Brüne PST |
| 14 | Brune, 2005 | Germany | Schizophrenia | 23 | 5 | 38.8 ± 12.2 | NR | 100 (MWT=27) | DSM-IV | NR | NR | Brüne PST |
| 15 | Brunet et al., 2003 | France | Healthy Controls | 25 | 8 | 42.6 ± 10.6 | 12.9 ± 2.5 | NR | DSM-IV | / | / | Brunet CST |
| 15 | Brunet et al., 2003 | France | Schizophrenia | 25 | 6 | 31.2 ± 8.7 | 11.8 ± 2.2 | NR | DSM-IV | NR | NR | Brunet CST |
| 16 | Champagne-Lavau et al., 2009 | Canada | Healthy Controls | 29 | 13 | 37.07 ± 14.86 | 13.7 ± 2.9 | NR | DSM-IV | / | / | Sarfati CST |
| 16 | Champagne-Lavau et al., 2009 | Canada | Schizophrenia | 31 | 11 | 42 ± 9.4 | 12.8 ± 2.2 | NR | DSM-IV | Excluded neurological diseases, Axis I or Axis II disorders | Olanzapine 25% Risperidone 12% Quetiapine 9% Clozapine 32% Risperidone and quetiapine 9%; Risperidone and clozapine 9% | Sarfati CST |
| 17 | Chan et al., 2022 | Hong Kong | Healthy Controls | 30 | 17 | 30.91 ± 10.68 | 14.07 ± 2.99 | NR | DSM-V | / | / | Sarfati CST |
| 17 | Chan et al., 2022 | Hong Kong | FEP | 30 | 17 | 36.33 ± 14.56 | 13.08 ± 3.35 | NR | DSM-V | Excluded organic brain syndrome, comorbid substance misuse or moderate learning disabilities | NR | Sarfati CST |
| 18 | Compère et al., 2016 | France | Healthy Controls | 21 | 11 | 11.1 ± | 15.48 ± 2.11 | NR | NR | / | / | Brunet CST |
| 18 | Compère et al., 2016 | France | Schizophrenia | 15 | 6 | 24.73 ± 10.8 | 13.2 ± 3.1 | NR | NR | Excluded any comorbidities | NR | Brunet CST |
| 19 | Corcoran et al., 1997 | UK | Healthy Controls | 40 | 23 | 48.37 ± 10.97 | NR | 108.1 | DSM-IV | / | / | Corcoran Joke |
| 19 | Corcoran et al., 1997 | UK | Schizophrenia | 44 | 13 | 30.71 ± 7.78 | NR | 78 | DSM-IV | NR | Antipsychotic (100%) | Corcoran Joke |
| 20 | Corcoran et al., 2008 | UK | Healthy Controls | 33 | 19 | 20.6274 ± 2.6111 | NR | 108.6 | DSM-IV | / | / | Langdon PST, Moore FBT |
| 20 | Corcoran et al., 2008 | UK | Depression | 27 | 18 | 39.03 ± 13.96 | NR | 108.2 | DSM-IV | Excluded presence of clear negative signs of psychosis or overt formal thought disorder | Antipsychotic (mean = 227.78mg, sd=261.14) | Langdon PST, Moore FBT |
| 20 | Corcoran et al., 2008 | UK | Depression | 20 | 9 | 36 ± 10.11 | NR | 98.5 | DSM-IV | Excluded presence of clear negative signs of psychosis or overt formal thought disorder | NR | Langdon PST, Moore FBT |
| 20 | Corcoran et al., 2008 | UK | Schizophrenia | 68 | 24 | 33.95 ± 8.38 | NR | 94.5 | DSM-IV | Excluded presence of clear negative signs of psychosis or overt formal thought disorder | NR | Langdon PST, Moore FBT |
| 21 | Danaher et al., 2018 | Australia | Healthy Controls | 46 | 18 | 23.21 ± 5.84 | NR | 108.7 | DSM-IV-TR | / | / | Langdon PST |
| 21 | Danaher et al., 2018 | Australia | FEP | 134 | 44 | 20.4025 ± 2.4362 | NR | NR | DSM-IV-TR | Substance use idsorder (n=31) | NR | Langdon PST |
| 22 | Demetriou et al., 2020 | Australia | Healthy Controls | 43 | 21 | 22.63 ± 5.55 | 12.86 ± 2 | NR | DSM-IV | / | / | Langdon PST |
| 22 | Demetriou et al., 2020 | Australia | ASD | 62 | 21 | 38 ± 9 | 13.37 ± 2.45 | NR | DSM-IV | NR | NR | Langdon PST |
| 22 | Demetriou et al., 2020 | Australia | FEP | 48 | 26 | 23.08 ± 5.76 | 13.07 ± 1.81 | NR | DSM-IV | NR | NR | Langdon PST |
| 23 | Docherty et al., 2013 | US | Healthy Controls | 21 | 11 | 41.95 ± 10.78 | 15 ± 2 | NR | DSM-IV | / | / | Sarfati CST |
| 23 | Docherty et al., 2013 | US | Schizophrenia/Schizoaffective | 63 | 21 | 40 ± 8 | 12 ± 2 | NR | DSM-IV | NR | NR | Sarfati CST |
| 24 | Fernandes et al., 2022 | Belgium | Healthy Controls | 30 | NR | NR | NR | NR | ADOS-2, ASDS, AQ | / | / | Brüne novel CST |
| 24 | Fernandes et al., 2022 | Belgium | ASD | 21 | NR | NR | NR | 101.6 | ADOS-2, ASDS, AQ | NR | Antidepressants (escitalopram, sertraline, venlafaxine, and mirtazapine) 22%, antipsychotics (risperidone, quetiapine, aripiprazole, and tiapride) 22%, anticonvulsants/ moodstabilizers (valproate, lamotrigine 19%), methylphenidate 9%, low-dose benzodiazepines (clobazam and ethylloflazepate) 9%, and clonidine 4% | Brüne novel CST |
| 25 | Gavilán et al., 2011 | Spain | Healthy Controls | 22 | 4 | 26 ± 4.155 | 10.05 ± 2.44 | 107.1 | ICD-10 | / | / | Happé cartoon, Langdon PST |
| 25 | Gavilán et al., 2011 | Spain | Schizophrenia | 22 | 4 | 42.82 ± 10.84 | 10.18 ± 2.38 | Premorbid: 106.4; WAIS-III = 87 | ICD-10 | Excluded history of central nervous system (CNS) disease or history of head injury, acute exacerbation stage of the illness, and physical disability (visual or auditory) | Antipsychotic (Chlorpromazine) | Happé cartoon, Langdon PST |
| 26 | Ghiassi et al., 2010 | Germany | Healthy Controls | 20 | 13 | 40.8 ± 12.7 | NR | NR | DSM-IV | / | / | Brüne PST |
| 26 | Ghiassi et al., 2010 | Germany | BPD | 50 | 46 | 26.18 ± 6.63 | NR | NR | DSM-IV | Excluded traumatic brain injury, intellectual disability, addiction disorders, or severe somatic disorders | Selective serotonin reuptake inhibitors 66%; noradrenaline and serotonin reuptake inhibitors 36%; tricyclic antidepressants, n4; second-generation antipsychotics 42%; benzodiazepines 12%; mood stabilizers 6% | Brüne PST |
| 27 | Haag et al., 2016 | Germany | Healthy Controls | 29 | 19 | 28.61 ± 8.1 | 14.8 ± 2.8 | NR | ICD-10 | / | / | Brunet CST |
| 27 | Haag et al., 2016 | Germany | Bipolar disorder | 29 | 13 | 47.8 ± 13.6 | 15 ± 2.4 | NR | ICD-10 | Excluded a history of substance  abuse or electroconvulsive therapy in the last 6 months,  and a previous diagnosis of schizoaffective disorder,  schizophrenia, antisocial personality disorder, dementia, mild cognitive impairment or mild intellectual disability | Mood stabilizers 100%, antipsychotics 10%, antidepressants 44%, anxiolytics 10%, hypnotics 3% | Brunet CST |
| 28 | Hillmann et al., 2020 | Germany | Healthy Controls | 41 | 41 | 25.4 ± 6.89 | NR | 114.76 | DSM-IV | / | / | Schnell cartoon |
| 28 | Hillmann et al., 2020 | Germany | BPD | 80 | 80 | 29.46 ± 8.07 | NR | 110.05 | DSM-IV | Affective disorder M = 28 (62), anxiety disorder M = 38 (42), substance abuse disorder M = 0 (26), eating disorder M = 20 (36), somatization disorder M = 3 (3), PTSD M = 21 (30), antisocial personality disorder M = 2 (4), avoidant personality disorder M = 17 (19) | NR | Schnell cartoon |
| 29 | Ho et al., 2015 | Hong Kong | Healthy Controls | 42 | 20 | 23.1 ± 2.96 | 13.83 ± 2.84 | 116.07 | DSM-IV | / | / | Yoni task |
| 29 | Ho et al., 2015 | Hong Kong | FEP | 41 | 18 | 27.66 ± 6.45 | 12.93 ± 3.33 | 110.32 | DSM-IV | Excluded (1) co-morbid DSM-IV Axis I disorder; (2) life-time history of any DSM-IV disorder apart from schizophrenia; (2) mental retardation; (3) severe hearing or visual impairment; (4) history of head injury or neurological disorders; (5) history of receiving electroconvulsive therapy in the past six months; and (6) lifetime history of alcohol and substance abuse | NR | Yoni task |
| 29 | Ho et al., 2015 | Hong Kong | Schizophrenia relatives | 43 | 25 | 27.33 ± 6.51 | 13.58 ± 2.43 | 113.95 | SCID-I | Excluded (1) co-morbid DSM-IV Axis I disorder; (2) life-time history of any DSM-IV disorder apart from schizophrenia; (2) mental retardation; (3) severe hearing or visual impairment; (4) history of head injury or neurological disorders; (5) history of receiving electroconvulsive therapy in the past six months; and (6) lifetime history of alcohol and substance abuse | NR | Yoni task |
| 30 | Hur et al., 2013 | Korea | Healthy Controls | 58 | 29 | 44.34 ± 10.65 | NR | 110.74 | DSM-IV | / | / | Sarfati CST (Korean) |
| 30 | Hur et al., 2013 | Korea | CHR | 55 | 18 | 21.96 ± 3.29 | NR | 109.76 | DSM-IV | Excluded head injury or medical or neurological disorders or alcohol or drug abuse | NR | Sarfati CST (Korean) |
| 31 | Inoue et al., 2004 | Japan | Healthy Controls | 50 | 22 | 27.93 ± 6.65 | NR | 107.34 | DSM-IV | / | / | Brüne PST (variation) |
| 31 | Inoue et al., 2004 | Japan | Depression (remitted) | 50 | 22 | 44.48 ± 8.46 | NR | 106.62 | DSM-IV | Excluded serious physical disease, psychiatric disorder, neurological disease, or substance abuse | SSRI 42%, tricyclics 22%, antidepressants 34%, Lithium12%, sodium valproate 20%, neuroleptics 12%, levomepromazine at a daily dose of 10 mg or lower 4%, and quetiapine at a daily dose of 50 mg or lower 8%. | Brüne PST (variation) |
| 32 | Inoue et al., 2006 | Japan | Healthy Controls | 30 | 25 | 22.28 ± 1.08 | 13.07 ± 2.08 | 102.03 | DSM-IV | / | / | Brüne PST (variation) |
| 32 | Inoue et al., 2006 | Japan | FEP | 30 | 25 | 27.03 ± 6.07 | 13.77 ± 1.94 | 103.07 | DSM-IV | Excluded serious physical disease, psychiatric disorder, neurological disease, or substance abuse | NR | Brüne PST (variation) |
| 33 | Kana et al., 2014 | US | Healthy Controls | 15 | NR | 36 ± 10.8 | NR | 112 | ADOS | / | / | Brunet CST |
| 33 | Kana et al., 2014 | US | ASD | 15 | NR | 21.14 ± 0.99 | NR | 106.93 | ADOS | NR | NR | Brunet CST |
| 34 | Kerr et al., 2003 | UK | Healthy Controls | 15 | 8 | 22.7 ± 3.5 | NR | 110.66 | DSM-IV | / | / | Frith FBT |
| 34 | Kerr et al., 2003 | UK | Bipolar disorder | 20 | 9 | 41.25 ± 12.2 | NR | 103.05 (NART) | DSM-IV | Excluded alcohol abuse, drug abuse or head injury | Antipsychotic (100%) | Frith FBT |
| 35 | Kim et al., 2011 | Korea | Healthy Controls | 45 | 17 | 37.76 ± 9.33 | 14.3 ± 1.6 | 111.6 | DSM-IV | / | / | Sarfati CST |
| 35 | Kim et al., 2011 | Korea | CHR | 36 | 15 | 21.1 ± 3.6 | 12.6 ± 2 | 112.2 | DSM-IV | NR | NR | Sarfati CST |
| 35 | Kim et al., 2011 | Korea | FEP | 13 | 4 | 21 ± 4.8 | 12.7 ± 1.9 | 96.9 | DSM-IV | NR | NR | Sarfati CST |
| 36 | Koelkebeck et al., 2018 | Germany | Healthy Controls | 25 | 9 | 20.4 ± 2.9 | 12.08 ± 1.5 | 116.56 | DSM-IV | / | / | Sarfati CST |
| 36 | Koelkebeck et al., 2018 | Germany | Schizophrenia | 25 | 10 | 40.6 ± 10.15 | 12.28 ± 1.81 | 112.04 (MWT-B) | DSM-IV | Organic impairments affecting brain function, alcohol or drug dependence or abuse, severe internal medical or neurological disorders | NR | Sarfati CST |
| 37 | Kong et al., 2021 | Korea | Healthy Controls | 28 | 9 | 34.54 ± 11.46 | 13.6 ± 1.9 | NR | SIPS/ SOPS | / | / | Brüne PST |
| 37 | Kong et al., 2021 | Korea | CHR | 28 | 9 | 20.3 ± 3.4 | 13.1 ± 1.7 | NR | SIPS/ SOPS | depressive disorder (n=14), anxiety disorder (n=6; social phobia, n=3; obsessive-compulsive disorder, n=1; panic disorder, n=1; post-traumatic stress disorder, n=1), somatoform disorder (n=1), and depersonalization disorder (n=1) | Antipsychotic 25% | Brüne PST |
| 38 | Langdon et al., 2001 | Australia | Healthy Controls | 24 | 12 | 35.5 ± 14.56 | 12.96 ± 2.03 | NR | DSM-IV | / | / | Langdon PST |
| 38 | Langdon et al., 2001 | Australia | Schizophrenia | 32 | 14 | 37.31 ± 10.74 | 11.91 ± 2.87 | NR | DSM-IV | Excluded current or past central nervous system disease or history of head injury, current substance abuse (as per DSM-IV), previous persistent substance abuse | Typical neuroleptics (haloperidol, flupenthixol, chlorpromazine): 18% atypicals (clozapine, olanzapine, risperidone): 78% | Langdon PST |
| 39 | Langdon et al., 2002 | Australia | Healthy Controls | 20 | NR | NR | NR | NR | DSM-IV | / | / | Langdon PST |
| 39 | Langdon et al., 2002 | Australia | Schizophrenia | 25 | NR | 23.2 | NR | NR | DSM-IV | Excluded current or past central nervous system disease or history of head injury, current substance abuse, previous persistent substance abuse | Typical neuroleptics (haloperidol, flupenthixol, and chlorpromazine): 28%,  atypicals (clozapine, olanzapine, and risperidone): 72% | Langdon PST |
| 40 | Langdon et al., 2005 | Australia | Healthy Controls | 38 | NR | 35.6 ± 14.6 | 12 ± 1.95 | 106.1 | DSM-IV | / | / | Langdon PST |
| 40 | Langdon et al., 2005 | Australia | Schizophrenia | 25 | NR | 33.5 ± 9.36 | 12.1 ± 2.218 | 106.1 | DSM-IV | Excluded current or past central nervous system disease or history of head injury, current substance abuse, a history of substance dependence or impaired general cognitive functioning | NR | Langdon PST |
| 41 | Langdon et al., 2006 | Australia | Healthy Controls | 18 | 9 | 32 ± 12.9 | NR | 109.9 | DSM-IV | / | / | Langdon PST |
| 41 | Langdon et al., 2006 | Australia | Schizophrenia | 22 | 10 | 40.5 ± 10.3 | NR | 103.9 (NART) | DSM-IV | Excluded current or past central nervous system disease, history of head trauma, current substance abuse , previous persistent substance abuse | Typical neuroleptics (haloperidol, flupenthixol, and chlorpromazine): 22%, second-generation atypicals (clozapine, olanzapine, and risperidone): 72% | Langdon PST |
| 42 | Langdon et al., 2010 | Australia | Healthy Controls | 34 | 8 | 20.79 ± 1.81 | NR | 105 | DSM-IV | / | / | Happé Cartoon, Langdon PST |
| 42 | Langdon et al., 2010 | Australia | Schizophrenia | 35 | 12 | 35.9 ± 10.4 | NR | 100 (NART) | DSM-IV | Excluded past history of central nervous system disease or head injury, current substance abuse | NR | Happé Cartoon, Langdon PST |
| 43 | Langdon et al., 2014 | Australia | Healthy Controls | 19 | 2 | 19.19 ± 3.01 | 12.82 ± 1.94 | 103.42 | ICD-10 | / | / | Happé Cartoon, Langdon PST |
| 43 | Langdon et al., 2014 | Australia | FEP | 23 | 1 | 20.91 ± 1.83 | 11.43 ± 2.02 | 96.65 (NART) | ICD-10 | NR | NR | Happé Cartoon, Langdon PST |
| 44 | Le Donne et al., 2023 | Italy | Healthy Controls | 32 | 7 | 30.21 ± 11.17 | 12.63 ± 2.83 | 95.7 | ADOS-2 | / | / | IA-CST |
| 44 | Le Donne et al., 2023 | Italy | ASD | 32 | 4 | 18.53 ± 2.53 | 10.84 ± 1.61 | 94.07 | ADOS-2 | Excluded concurrent psychiatric or medical conditions and cognitive impairment | NR | IA-CST |
| 45 | Lee et al., 2014 | Korea | Schizophrenia Relatives | 21 | 16 | 52.86 ± 13.16 | 13.05 ± 3.99 | NR | DSM-IV | NR | NR | Sarfati CST (modified) |
| 45 | Lee et al., 2014 | Korea | Schizophrenia | 25 | 13 | 35.72 ± 11.33 | 12.2 ± 3.35 | NR | DSM-IV | NR | NR | Sarfati CST (modified) |
| 46 | Lemvigh et al., 2022 | Australia | Healthy Controls | 34 | 19 | 35.82 ± 11.85 | 14.48 ± 4.31 | 114.91 | DSM-IV-TR | / | / | Yoni task |
| 46 | Lemvigh et al., 2022 | Australia | Bipolar disorder | 64 | 31 | 37.11 ± 11.21 | NR | 107.69 (WTAR) | DSM-IV-TR | Excluded neurological/neurodegenerative disease, a history of severe head injury (loss of consciousness), hearing or visual impairments, pregnancy, language difficulties, a history of habitual drug use or dependence | Mood stabilizer (61%), antidepressants (31%), typical antipsychotics (3%), atypical antipsychotics (47%) | Yoni task |
| 47 | Li et al., 2017 | China | Healthy Controls | 35 | 15 | 16.31 ± 1.21 | 10.17 ± 1.27 | NR | NR | / | / | Yoni task |
| 47 | Li et al., 2017 | China | FEP | 35 | 15 | 16.48 ± 1.42 | 9.68 ± 1.18 | NR | NR | Excluded a history of drug and alcohol abuse, head trauma, seizure disorder, other major neurological disorder, mental retardation | NR | Yoni task |
| 48 | Liang et al., 2022 | China | Healthy Controls | 42 | 28 | 23.29 ± 4.19 | 15.83 ± 1.96 | 128.1 | ICD-10 | / | / | Yoni task |
| 48 | Liang et al., 2022 | China | Bipolar disorder | 37 | 22 | 24.65 ± 6.07 | 15.32 ± 3.25 | 128.1 | ICD-10 | Excluded (a) a history of head trauma or neurological disorders; (b) a history of substance or alcohol dependence; (c) mental retardation; (d) presence of metal in the body; (e) claustrophobia; (f) structural abnormalities of the brain | NR | Yoni task |
| 49 | Liu et al, 2017 | China | Healthy Controls | 38 | 22 | 23.32 ± 2.68 | 16.29 ± 1.56 | 117.42 | DSM-IV | / | / | Yoni task |
| 49 | Liu et al, 2017 | China | OCD | 40 | 22 | 24.6 ± 4.12 | 15.85 ± 2.13 | 113.73 | DSM-IV | Excluded (1) comorbid psychiatric disorders (Axis I or Axis II); (2) history of specific medical or neurological problems, such as hyperactivity, organic mental disease, mental retardation, history of psychosurgery, or history of epilepsy; (3) history of taking psychoactive medication in the past 3 months; and (4) severe alcohol or substance abuse. | NR | Yoni task |
| 50 | Livingston et al., 2024 | UK | Healthy Controls | 165 | 80 | 28.8 ± 12.07 | NR | NR | NR | / | / | Happé cartoon (modified) |
| 50 | Livingston et al., 2024 | UK | ASD | 72 | 36 | 31.76 ± 11.67 | NR | NR | NR | NR | NR | Happé cartoon (modified) |
| 51 | Marjoram et al., 2005 | UK | Healthy Controls | 20 | 9 | 39.8 ± 13.2 | 13.5 ± 2.5 | 100 | DSM-IV | / | / | Happé cartoon |
| 51 | Marjoram et al., 2005 | UK | schizophrenia | 20 | 8 | 39.8 ± 11.6 | 13.3 ± 2.9 | 97 | DSM-IV | NR | NR | Happé cartoon |
| 52 | Marjoram et al., 2006 | UK | Healthy Controls | 13 | 5 | 29.6 ± 1.6 | NR | 106.8 | ICD-10 | / | / | Happé cartoon |
| 52 | Marjoram et al., 2006 | UK | CHR | 24 | 11 | 29.85 ± 3.098 | NR | 101.3 (NART) | ICD-10 | NR | Antispsychotic (12.5%) | Happé cartoon |
| 53 | Mattern et al., 2014 | Germany | Healthy Controls | 26 | 15 | 40.1 ± 12.4 | NR | NR | DSM-IV | / | / | Schnell cartoon |
| 53 | Mattern et al., 2014 | Germany | Depression | 26 | 15 | 40.8 ± 10.7 | NR | NR | DSM-IV | Excluded Acute risk for suicide; history of psychotic symptoms, bipolar disorder or dementia; severe substance-related abuse or dependence disorder; schizotypal, antisocial or borderline personality disorder; serious medical condition; severe cognitive impairment | NR | Schnell cartoon |
| 54 | Mehl et al., 2020 | Germany | Healthy Controls | 48 | 20 | 35.69 ± 9.44 | NR | 114.52 | DSM-IV | / | / | Schnell cartoon (14) |
| 54 | Mehl et al., 2020 | Germany | Schizophrenia | 185 | 107 | 38.62 ± 9.78 | NR | 176 (MWT-B) | DSM-IV | NR | NR | Schnell cartoon (14) |
| 55 | Moore et al., 2006 | UK | Healthy Controls | 30 | 22 | 75.73 ± 5.59 | 10.73 ± 2.42 | NR | DSM-IV | / | / | Moore FBT |
| 55 | Moore et al., 2006 | UK | Depression | 30 | 16 | 77.07 ± 8.52 | 9.63 ± 1.67 | 93.87 | DSM-IV | NR | NR | Moore FBT |
| 55 | Moore et al., 2006 | UK | FEP | 29 | 19 | 76.9 ± 5.99 | 9.83 ± 2.19 | 93.1 | DSM-IV | NR | NR | Moore FBT |
| 56 | Morrison et al., 2019 (Pinkham et al., 2019) | US | Healthy Controls | 95 | 11 | 24.17 ± 6.21 | NR | 116.28 | ADOS-2 | / | / | Brüne PST (intention subscale) |
| 56 | Morrison et al., 2019 (Pinkham et al., 2019) | US | ASD | 103 | 11 | 24.28 ± 6.17 | NR | 108.9 | ADOS-2 | NR | NR | Brüne PST (intention subscale) |
| 57 | Ohmuro et al., 2016 | Japan | Healthy Controls | 25 | 14 | 21.3 ± 1 | 14.4 ± 0.9 | 112 | DSM-IV | / | / | Brüne PST |
| 57 | Ohmuro et al., 2016 | Japan | CHR | 36 | 22 | 20.9 ± 4.7 | 12.5 ± 2.4 | 101.1 | DSM-IV | Excluded (i) serious suicide or violence risk, due to a personality disorder; (ii) current substance dependence; (iii) intellectual disability (IQo70); (iv) comorbid diagnosis of autistic spectrum disorders, or (v) neurological disorder, head injury, or any other significant medical condition associated with psychiatric symptoms. | Antipsychotic (22%) | Brüne PST |
| 57 | Ohmuro et al., 2016 | Japan | FEP | 40 | 29 | 22.9 ± 6.3 | 12.5 ± 2 | 99.1 | DSM-IV | Excluded (i) serious suicide or violence risk, due to a personality disorder; (ii) current substance dependence; (iii) intellectual disability (IQo70); (iv) comorbid diagnosis of autistic spectrum disorders, or (v) neurological disorder, head injury, or any other significant medical condition associated with psychiatric symptoms. | Antipsychotic (95%) | Brüne PST |
| 58 | Olley et al., 2005 | Australia | Healthy Controls | 13 | 7 | 40.77 ± 13.42 | NR | 108.15 | DSM-IV | / | / | Happé cartoon (modified) |
| 58 | Olley et al., 2005 | Australia | Bipolar disorder | 15 | 8 | 39.2 ± 11.83 | NR | 106.4 (NART) | DSM-IV | Excluded Those with neurological conditions, a history of significant drug and/or alcohol use, a history of electroconvulsive therapy (ECT) in the previous 6 months, or those who had sustained a traumatic brain injury | NR | Happé cartoon (modified) |
| 59 | Park et al., 2021 | Korea | Healthy Controls | 24 | 8 | 21.8 ± 3.3 | 13.3 ± 1.2 | NR | DSM-IV | / | / | Brüne PST |
| 59 | Park et al., 2021 | Korea | CHR | 24 | 8 | 21.9 ± 3.8 | 13.1 ± 2.2 | NR | DSM-IV | NR | Antipsychotic (20%) | Brüne PST |
| 60 | Pinkham et al., 2019 | US | Healthy Controls | 101 | 16 | 24.62 ± 5.82 | 14.55 ± 1.74 | 106.62 | ADOS | / | / | Brüne PST (intention subscale 14) |
| 60 | Pinkham et al., 2019 | US | ASD | 101 | 11 | 24.23 ± 6.18 | 13.63 ± 1.72 | 104.23 (WRAT-3) | ADOS | Excluded : (1) presence or history of intellectual disability (ID) (defined as IQ < 70), (2) presence or history of medical or neurological disorders that may affect brain function (e.g. uncontrolled hypertension, history of seizures, head trauma with unconsciousness for more than 15 min), (3) visual or hearing limitation that would interfere with assessment, and (4) current substance use disorder, except for nicotine. | NR | Brüne PST (intention subscale 14) |
| 60 | Pinkham et al., 2019 | US | Schizophrenia | 92 | 27 | 27.77 ± 7.28 | 13.57 ± 1.76 | 106.1 (WRAT-3) | DSM-IV | Excluded : (1) presence or history of intellectual disability (ID) (defined as IQ < 70), (2) presence or history of medical or neurological disorders that may affect brain function (e.g. uncontrolled hypertension, history of seizures, head trauma with unconsciousness for more than 15 min), (3) visual or hearing limitation that would interfere with assessment, and (4) current substance use disorder, except for nicotine. | NR | Brüne PST (intention subscale 14) |
| 61 | Piovan et al., 2016 | Italy | Healthy Controls | 24 | 12 | 46.37 ± 7.92 | 10.7 ± 2.89 | 109.66 | DSM-IV-TR | / | / | Brüne PST |
| 61 | Piovan et al., 2016 | Italy | Schizophrenia | 30 | 11 | 45.4 ± 8.39 | 9.6 ± 2.73 | 106.79 | DSM-IV-TR | NR | NR | Brüne PST |
| 62 | Popolo et al., 2016 | Italy | Healthy Controls | 40 | NR | 35.8 ± 13.6 | NR | NR | DSM-IV | / | / | Brüne PST |
| 62 | Popolo et al., 2016 | Italy | Schizophrenia | 37 | NR | 27.19 ± 6.57 | NR | NR | DSM-IV | Excluded the presence of an intellectual disability, neurological disorder, or substance dependence | NR | Brüne PST |
| 63 | Popolo et al., 2020 | Italy | Healthy Controls | 45 | 27 | 47.27 ± 9.41 | 13.87 ± 3.2 | NR | DSM-IV-TR | / | / | Brüne PST |
| 63 | Popolo et al., 2020 | Italy | Bipolar disorder | 45 | 25 | 47.2 ± 11.64 | 11.62 ± 2.94 | NR | DSM-IV-TR | Excluded an intellectual disability, neurological disorders, substance dependence within the last month or evidence of cognitive impairment precluding informed consent. | NR | Brüne PST |
| 64 | Pousa et al., 2008 | Spain | Healthy Controls | 51 | 13 | 34.6 ± 8.3 | NR | 93.4 | DSM-IV | / | / | Langdon PST |
| 64 | Pousa et al., 2008 | Spain | Schizophrenia | 61 | 13 | 32.5 ± 7.8 | NR | 87.5 | DSM-IV | Excluded current or past CNS disease or history of head injury, physical disability | conventional 16%, atypical 63%, mixed 19% | Langdon PST |
| 65 | Rahamim et al., 2024 | Israel | Healthy Controls | 29 | 15 | 25.9 ± 6.73 | NR | NR | DSM-V | / | / | Brüne CST |
| 65 | Rahamim et al., 2024 | Israel | Schizophrenia | 25 | 4 | 28.28 ± 5.05 | 11 ± 1.71 | NR | DSM-V | Excluded severe depression and general cognitive impairments. | NR | Brüne CST |
| 66 | Reynolds et al., 2014 | Australia | Healthy Controls | 20 | NR | 31.75 ± 11.94 | 13.75 ± 1.97 | 116.65 | DSM-IV-TR | / | / | Langdon PST |
| 66 | Reynolds et al., 2014 | Australia | Bipolar first-degree relatives | 20 | NR | 34.15 ± 13.28 | 15.7 ± 6.4 | 114.45 | DSM-IV-TR | Excluded a history of traumatic brain injury, epilepsy, pregnancy, vision/hearing impairments, alcohol/substance dependence or abuse in the previous six months, neurological or degenerative disease and current or previous psychiatric medication use | NR | Langdon PST |
| 67 | Russell et al., 2009 | UK | Healthy Controls | 22 | 22 | 30.3 ± 6.5 | 12.4 ± 1.5 | 119.6 | DSM-IV | / | / | Happé cartoon |
| 67 | Russell et al., 2009 | UK | Anorexia nervosa | 22 | 22 | 26.7 ± 4.8 | 14.1 ± 2.2 | 118.3 (NART) | DSM-IV | Excluded individuals with brain damage or psychosis | NR | Happé cartoon |
| 68 | Samson & Hegenloh, 2010 | Germany | Healthy Controls | 109 | 67 | 24.99 ± 5.67 | 15.7 ± 1.5 | NR | ICD-10 | / | / | Cartoon Humor (Samson) |
| 68 | Samson & Hegenloh, 2010 | Germany | ASD | 19 | 10 | 27.79 ± 8.28 | NR | NR | ICD-10 | NR | NR | Cartoon Humor (Samson) |
| 69 | Sarfati et al., 1997 | France | Healthy Controls | 24 | 10 | 32.4 ± 13.1 | 12.9 ± 2.2 | NR | DSM-III-R | / | / | Sarfati CST |
| 69 | Sarfati et al., 1997 | France | Schizophrenia | 24 | 5 | 31.9 ± 11.8 | 12.2 ± 2.1 | 24.7 (Binois-Pichot) | DSM-III-R | NR | NR | Sarfati CST |
| 69 | Sarfati et al., 1997 | France | Depression | 12 | 9 | 41.9 ± 8.4 | 12.2 ± 4.4 | 27.9 (Binois-Pichot) | DSM-III-R | NR | NR | Sarfati CST |
| 70 | Sayın et al., 2010 | Turkey | Healthy Controls | 30 | 20 | 33 ± 10.62 | NR | NR | DSM-IV-TR | / | / | Brüne PST |
| 70 | Sayın et al., 2010 | Turkey | OCD | 30 | 20 | 34.3 ± 11.49 | NR | NR | DSM-IV-TR | Excluded comorbid depression and/or substance abuse | NR | Brüne PST |
| 71 | Shamay-Tsoory et al., 2007 | Israel | Healthy Controls | 55 | 29 | 28.62 ± 9.01 | 13.38 ± 1.4 | NR | DSM-IV | / | / | Yoni task |
| 71 | Shamay-Tsoory et al., 2007 | Israel | Schizophrenia | 22 | 9 | 32.56 ± 10.83 | 12.67 ± 3.23 | NR | DSM-IV | Excluded Patients with other neurological problems or a major physical illness, alcohol or substance abuse | NR | Yoni task |
| 72 | Stanford et al., 2011 | US | Healthy Controls | 24 | 9 | 21 ± 3.6 | NR | 107.5 | SIPS/ SOPS | / | / | Cartoon FBT - 1st & 2nd |
| 72 | Stanford et al., 2011 | US | CHR | 63 | 13 | 19.6 ± 3.6 | NR | 104.2 | SIPS/ SOPS | Excluded any major medical or neurological disorder | NR | Cartoon FBT - 1st & 2nd |
| 73 | Tchanturia et al., 2004 | UK | Healthy Controls | 20 | 20 | 28.3 ± 7.4 | NR | NR | DSM-IV | / | / | Happé cartoon |
| 73 | Tchanturia et al., 2004 | UK | Anorexia nervosa | 20 | 20 | 27.4 ± 7.9 | NR | NR | DSM-IV | Excluded developmental disorders, learning disability or substance dependence | NR | Happé cartoon |
| 74 | Thompson et al., 2012 | Australia | Healthy Controls | 30 | 18 | 19.3 ± 2.9 | 13.2 ± 2 | 104.9 | CAARMS | / | / | Corcoran Joke |
| 74 | Thompson et al., 2012 | Australia | CHR | 30 | 16 | 19.1 ± 2.8 | 12 ± 1.9 | 103.3 | CAARMS | Excluded intellectual disability, epilepsy, visual acuity, auditory acuity | Antipsychotic (10%) | Corcoran Joke |
| 74 | Thompson et al., 2012 | Australia | FEP | 30 | 15 | 20.5 ± 2.5 | 12.5 ± 1.6 | 106.4 | CAARMS | Excluded intellectual disability, epilepsy, visual acuity, auditory acuity | Antipsychotic (75%) | Corcoran Joke |
| 75 | Tin et al., 2018 | Hong Kong | Healthy Controls | 30 | 9 | 17.17 ± 1.02 | 11.13 ± 0.97 | 114.83 | DSM-IV | / | / | Yoni task |
| 75 | Tin et al., 2018 | Hong Kong | ASD | 30 | 7 | 17.03 ± 0.93 | 10.9 ± 0.96 | 103.93 | DSM-IV | Excluded (1) presence of any co-morbid DSM-IV disorder; (2) mental retardation; (3) severe hearing or visual impairment; (4) history of head injury; (5) history of neurological disorder; and (6) history of substance abuse | NR | Yoni task |
| 75 | Tin et al., 2018 | Hong Kong | FEP | 30 | 11 | 17.47 ± 1.22 | 11.43 ± 1.41 | 113.03 | DSM-IV | Excluded (1) presence of any co-morbid DSM-IV disorder; (2) mental retardation; (3) severe hearing or visual impairment; (4) history of head injury; (5) history of neurological disorder; and (6) history of substance abuse | NR | Yoni task |
| 76 | Tsoi et al., 2008 | UK | Healthy Controls | 30 | 9 | 38.1 ± 12.4 | NR | 110 | DSM-IV | / | / | Brüne PST |
| 76 | Tsoi et al., 2008 | UK | Schizophrenia | 30 | 9 | 42.1 ± 9.3 | NR | 106 (NART) | DSM-IV | Excluded e presence of a history of neurological disorders (e.g. head injuries, epilepsy) or learning disability, and a current diagnosis of alcohol or drug dependence | Antipsychotic (100%) | Brüne PST |
| 77 | Tsui et al., 2024 | Hong Kong | Healthy Controls | 40 | 21 | 25.25 ± 7.53 | 15.1 ± 2.44 | NR | DSM-V | / | / | Sarfati CST |
| 77 | Tsui et al., 2024 | Hong Kong | CHR | 40 | 24 | 23.78 ± 7.95 | 12.6 ± 2.65 | NR | DSM-V | NR | Antipsychotic (47.5%) | Sarfati CST |
| 77 | Tsui et al., 2024 | Hong Kong | FEP | 40 | 21 | 24.6 ± 6.28 | 13.05 ± 2.98 | NR | DSM-V | Excluded (1) the presence of pervasive developmental disorders; (2) other major psychiatric disorders, such as mood and anxiety spectrum disorders; (3) a history of substance use or neurological disorders; (4) any auditory, speech, or visual impairments; and (5) moderate to severe learning disability. | Antipsychotic (95%) | Sarfati CST |
| 78 | Tulacı et al., 2018 | Turkey | OCD | 80 | NR | NR | NR | NR | DSM-IV | NR | Antipsychotic (25%) | Frith FBT |
| 78 | Tulacı et al., 2018 | Turkey | OCD | 80 | NR | NR | NR | NR | DSM-IV | n=46 with comorbidity | Antipsychotic (25%) | Frith FBT |
| 79 | Van Rheenen et al., 2013 | Australia | Healthy Controls | 49 | 31 | 34.65 ± 14.43 | NR | 111.692 | DSM-IV-TR | / | / | Langdon PST |
| 79 | Van Rheenen et al., 2013 | Australia | Bipolar disorder | 49 | 33 | 38.45 ± 13.2 | NR | 109.2 | DSM-IV-TR | Excluded visual impairments, neurological disorder and/or a history of substance/alcohol abuse or dependence during the past six months | NR | Langdon PST |
| 80 | Vistoli et al., 2011 | France | Healthy Controls | 21 | 7 | 28 ± 5.74 | NR | NR | DSM-IV | / | / | Brunet CST |
| 80 | Vistoli et al., 2011 | France | Schizophrenia | 19 | 6 | 36 ± 10.18 | NR | NR | DSM-IV | NR | NR | Brunet CST |
| 81 | Walter et al., 2009 | Germany | Healthy Controls | 12 | 6 | 24.75 ± 2.6 | 12.3 ± 1.4 | NR | DSM-IV | / | / | Walter CST |
| 81 | Walter et al., 2009 | Germany | schizophrenia | 12 | 6 | 29.5 ± 6 | 12.4 ± 1.1 | NR | DSM-IV | Excluded Patients diagnosed with concurrent axis I disorder according to DSM-IV criteria | NR | Walter CST |
| 82 | Wang et al., 2018 | China | Healthy Controls | 35 | 19 | 27.26 ± 6.7 | 13.26 ± 2.49 | 106.94 | DSM-IV | / | / | Yoni task |
| 82 | Wang et al., 2018 | China | Bipolar disorder | 35 | 19 | 31.11 ± 6.816 | 12.74 ± 3.73 | 112.2 | DSM-IV | Excluded a co-morbid DSM-IV Axis I disorder; a history of head injury or neurological disorder; a lifetime history of alcohol and substance abuse; severe hearing or visual impairment | NR | Yoni task |
| 82 | Wang et al., 2018 | China | Depression | 35 | 20 | 29.4 ± 7.93 | 11.6 ± 2.64 | 109.32 | DSM-IV | Excluded a co-morbid DSM-IV Axis I disorder; a history of head injury or neurological disorder; a lifetime history of alcohol and substance abuse; severe hearing or visual impairment | NR | Yoni task |
| 82 | Wang et al., 2018 | China | FEP | 35 | 21 | 29.14 ± 5.791 | 11.66 ± 2.51 | 107.74 | DSM-IV | Excluded a co-morbid DSM-IV Axis I disorder; a history of head injury or neurological disorder; a lifetime history of alcohol and substance abuse; severe hearing or visual impairment | NR | Yoni task |
| 83 | Willert et al., 2015 | Germany | Healthy Controls | 81 | 41 | 35.57 ± 9.34 | 15.14 ± 2.62 | NR | DSM-IV | / | / | Schnell cartoon |
| 83 | Willert et al., 2015 | Germany | Bipolar disorder | 24 | 12 | 44.75 ± 12.21 | 17.75 ± 2.93 | NR | DSM-IV | Excluded significant general medical illness, including neurological disorders and head trauma | Mood Stabilizers (70%): Lithium (37%) Lamotrigine (20%) Valproate (20%) Carbamazepine (4%) Antipsychotic Medication (70%): Aripiprazole (50%) Quetiapine (37%) Olanzapine (4%) Amisulpride (4%) Antidepressants (25%): Citalopram (8%) Venlafaxine (12%) Trimipramine (4%) | Schnell cartoon |
| 83 | Willert et al., 2015 | Germany | Bipolar first-degree relatives | 21 | 14 | 31 ± 11.17 | 15.64 ± 2.54 | NR | DSM-IV | Excluded significant general medical illness, including neurological disorders and head trauma | NR | Schnell cartoon |
| 84 | Wolf et al., 2010 | Germany | Healthy Controls | 29 | 19 | 37 ± 13.74 | NR | 111.59 | DSM-IV | / | / | Brüne PST |
| 84 | Wolf et al., 2010 | Germany | Bipolar disorder | 33 | 22 | 47.7 ± 14 | NR | 107.85 (MWT-B); 8.52 (HAWIE subset) | DSM-IV | Excluded substance abuse, traumatic brain injuries or learning disabilities | Lithium Carbonate (21%) Other Mood Stabilizers (33%): Mainly Valproic Acid Second-Generation Antipsychotics (84%) Antidepressants (45%) | Brüne PST |
| 85 | Wu et al., 2023 | China | Healthy Controls | 30 | 15 | 34.7 ± 9.033 | 12.1 ± 3.478 | NR | DSM-V | / | / | Yoni task |
| 85 | Wu et al., 2023 | China | Schizophrenia | 30 | 14 | 37.57 ± 10.944 | 11.6 ± 2.621 | NR | DSM-V | Excluded history of head injury or  neurological disorder; lifetime history of alcohol and  substance abuse; severe auditory or visual impairment;  previous serious suicide attempts; and severe agitation | NR | Yoni task |
| 86 | Yang et al., 2017 | China | Healthy Controls | 38 | 17 | 30.32 ± 9.15 | 13.66 ± 2.29 | 105.24 | ICD-10 | / | / | Brüne PST |
| 86 | Yang et al., 2017 | China | FEP | 35 | 20 | 28.46 ± 7.94 | 13.14 ± 2.74 | 92.71 | ICD-10 | Excluded (1) diagnosis of drug or alcohol dependency, (2) reported history of a neurological disorder or severe head injury, and (3) presence of other severe physical diseases | Antipsychotic (94%) | Brüne PST |
| 87 | Yeh et al., 2017 | Taiwan | Healthy Controls | 36 | 33 | 29.69 ± 7.14 | 14.33 ± 1.99 | NR | DSM-IV | / | / | Gallagher cartoon |
| 87 | Yeh et al., 2017 | Taiwan | BPD | 40 | 37 | 30.88 ± 7.26 | 13.4 ± 2.33 | NR | DSM-IV | Excluded schizophrenia, substance use disorder, brain injury or neurological disorders | NR | Gallagher cartoon |
| 87 | Yeh et al., 2017 | Taiwan | Depression | 34 | 32 | 31.38 ± 8.16 | 14.52 ± 2.29 | NR | DSM-IV | Excluded schizophrenia, substance use disorder, brain injury or neurological disorders | NR | Gallagher cartoon |
| 88 | Ziermans et al., 2020 | the Netherlands | Healthy Controls | 337 | 184 | 38.5 ± 10.6 | NR | 115.4 | DSM-IV-TR | / | / | Langdon PST |
| 88 | Ziermans et al., 2020 | the Netherlands | Schizophrenia | 504 | 139 | 33.4 ± 7.2 | NR | 100.7 | DSM-IV-TR | NR | Antipsychotic (68%) | Langdon PST |
| 88 | Ziermans et al., 2020 | the Netherlands | Schizophrenia relatives | 572 | 218 | 34 ± 7.9 | NR | 111.5 | DSM-IV-TR | NR | NR | Langdon PST |
| 89 | Zobel et al., 2010 | Germany | Healthy Controls | 30 | 16 | 46.2 ± 12.1 | NR | 118 | DSM-IV | / | / | Brüne PST |
| 89 | Zobel et al., 2010 | Germany | Depression | 30 | 17 | 46.7 ± 11.9 | NR | 113 | DSM-IV | Axis I (n=7); Axis II (n=3) | Antidepressants (93%), venlafaxine (27%) and sertraline (23%), benzodiazepines (6.67%) | Brüne PST |

Note. AQ indicates Autism Spectrum Quotient; ADI, Autism Diagnostic Interview; ADOS, Autism Diagnostic Observation Schedule; BPD, Borderline Personality Disorder; CAARMS, Comprehensive Assessment of At-Risk Mental States; CHR, Clinical High-Risk; CST, Comic Strip Task; DSM, Diagnostic and Statistical Manual of Mental Disorders; ICD, International Classification of Diseases; IQ, Intelligence Quotient; NR, Not Reported; OCD, Obsessive-Compulsive Disorder; PST, Picture Sequencing Task; SCID, Structured Clinical Interview for DSM; SIPS/SOPS, Structured Interview for Psychosis-Risk Syndromes.

# Supplementary Table 2. Characteristics of included studies by conditions

| Conditions | Number of studies | Sample size | Sex (Female %) | Mean age (Range) | Mean years of education |
| --- | --- | --- | --- | --- | --- |
| Anorexia Nervosa | 2 | 42 | 100% | 27.05 (26.7-27.4) | 14.1 |
| ASD | 10 | 520 | 32.5% | 23.58 (17.03-31.76) | 12.63 |
| Bipolar disorder | 11 | 366 | 50.7% | 39.57 (24.65-47.80) | 14.39 |
| BPD | 4 | 200 | 96.1% | 28.06 (26.18-30.88) | 13.40 |
| CHR | 11 | 448 | 42.2% | 22.05 (19.1-29.85) | 12.27 |
| Depression | 10 | 286 | 61.1% | 45.52 (29.4-77.07) | 12.27 |
| Early Schizophrenia | 19 | 685 | 44.5% | 26.95 (16.48-36.33) | 12.20 |
| FHR-B | 2 | 41 | 66.7% | 32.58 (31.0-34.15) | 15.67 |
| FHR-S | 4 | 730 | 54.5% | 47.36 (27.33-62.4) | 11.46 |
| OCD | 3 | 150 | 60.8% | 29.45 (24.6-34.3) | 15.85 |
| Schizophrenia | 35 | 1855 | 33.6% | 35.11 (23.2-45.4) | 11.97 |
| Total | 89 | 9038 | 48.4% | 32.19 (16.48-77.07) | 12.90 |

# Supplementary Table 3. Characteristics of included mentalizing tasks

| **Task** | **Reference** | **Stimuli** | **Format** | **Types** | **No. of condition** | **Condition(s)** | **Total score** |
| --- | --- | --- | --- | --- | --- | --- | --- |
| **Brüne novel CST** | Brüne, M., Walden, S., Edel, M. A., & Dimaggio, G. (2016). Mentalization of complex emotions in borderline personality disorder: The impact of parenting and exposure to trauma on the performance in a novel cartoon-based task. *Comprehensive psychiatry*, *64*, 29-37. | Coloured cartoon picture story | Participants were instructed to quickly sequence seven cards while prioritizing accuracy over speed. An eighth card presented a potential story ending, from which Participants chose one of four options: one “prosocial” (e.g., a character is comforted), one “antisocial” (e.g., a character is excluded), one “avoidant” (e.g., a character leaves unresolved), and one “disorganized” (e.g., a character displays nonsensical behaviour). | Intentionality | 3 | Sequencing, cognitive mental states, affective mental states | Sequencing = 28 Cognitive = 16 Affective = 16 |
| **Brüne PST** | Brüne, M. (2003). Theory of mind and the role of IQ in chronic disorganized schizophrenia. *Schizophrenia research*, *60*(1), 57-64. | Black and white cartoon picture story | Participants were asked to order the cards in a logical sequence of events and answer to ToM questionnaire comprising 23 questions, including control reality questions. | False beliefs | 3 | First to third false belief, cheating detection and reality questions, included to rule out major attention problems | 59 |
| **Brunet CST** | Brunet, E., Sarfati, Y., & Hardy-Baylé, M. C. (2003). Reasoning about physical causality and other's intentions in schizophrenia. Cognitive neuropsychiatry, 8(2), 129-139. | Black and white comic | Three consecutive comics were given as the story, then choose a logical answers as an end among three options | Intentionality | 3 (version 1):  2 (version 2; Brunet et al., 2021) | Version 1: 1. Attribution of intention 2. Physical causality with characters 3. Physical causality involving objects  Version 2: 1. Attribution of intention 2. Physical causality | 14*3 = 42 (Version 1) 10*3 = 30 (Version 3; Duval et al., 2012) 6*2 = 12 (Version 4; Compere et al., 2016) 29 (IA) + 29 (PCCH) = 58 (Redrawn; Roux et al., 2016) 34 (AI) + 20 (PCCH) + 20 (PCOB) = 74 (Vistoli et al., 2011) 11+11 = 22 (Kana et al., 2014) 20 (Physical causality) + 14 (intention attribution) = 34 (Version 2) |
| **Cartoon Humor Samson** | Samson, A. C., Zysset, S., & Huber, O. (2008). Cognitive humor processing: different logical mechanisms in nonverbal cartoons—an fMRI study. Social neuroscience, 3(2), 125-140. | Black and white cartoon | Participants were instructed to indicate their understanding of the joke in the cartoon by pressing a button, with each cartoon displayed for 6 seconds. They were also asked to rate the funniess based on each cartoons provided. | Humor | 5 | Visual puns (PUN), Semantic cartoons (SEM), Theory of Mind cartoons (TOM), Irresolvable incongruity (INC), Control (BAS) | Comprehensibility = 180 Funniness = 90*6 |
| **Corcoran Joke** | Corcoran, R., Cahill, C., & Frith, C. D. (1997). The appreciation of visual jokes in people with schizophrenia: a study of ‘mentalizing’ability. Schizophrenia research, 24(3), 319-327. | Black and white comic | Two sets of jokes were shown and asked to explain each jokes | Humor | 2 | Theory of Mind, physical/ behavioural | 10*2 = 20 |
| **Frith FBT** | Frith CD, Corcoran R. Exploring ‘theory of mind’ in people with schizophrenia. Psychological Medicine. 1996;26(3):521-530. doi:10.1017/S0033291700035601 | Black and white cartoon | Participants listened to six theory of mind stories while viewing cartoon drawings depicting the action sequences. After each story, two questions were posed: one regarding the characters' mental states and another about the reality of the events. | False beliefs | 2 | First and second false belief and deception | 6*2 = 12 |
| **Gallagher cartoon** | Gallagher, H. L., Happé, F., Brunswick, N., Fletcher, P. C., Frith, U., & Frith, C. D. (2000). Reading the mind in cartoons and stories: an fMRI study of ‘theory of mind’in verbal and nonverbal tasks. Neuropsychologia, 38(1), 11-21. | Black and white cartoon | Participants were instructed to view each cartoon and signal to the experimenter upon understanding its meaning. They then provided a brief explanation of the cartoon and rated its difficulty and humor. | Humor | 3 | Theory of Mind cartoons, Non-Theory of Mind cartoons, Jumbled pictures | 28*3 = 84 |
| **Happé cartoon** | Happé, F., Brownell, H., & Winner, E. (1999). Acquired theory of mind impairments following stroke. *Cognition*, *70*(3), 211-240. | Black and white cartoon | Participants were allowed to spend as long as necessary reading to guarantee their memory for background information in the stories. Next, they answered questions about why story characters acted in certain ways or said something, without referring back to the stories. Time to read each story and answer will be recorded and rated. | Humor | 3 | ToM joke (attribution of false belief, ignorance or deception).  Physical joke (did not require ToM abilities). Jumbled image (control) | 6*3 = 18 Modified (Livingston et al., 2024): 14 |
| **Langdon PST** | Langdon, R., Michie, P. T., Ward, P. B., McConaghy, N., Catts, S. V., & Coltheart, M. (1997). Defective self and/or other mentalising in schizophrenia: a cognitive neuropsychological approach. *Cognitive Neuropsychiatry*, *2*(3), 167-193. | Black and white comic | After two practices, sequences were represented in a random order. Cards were placed face-down and subjects were asked to turn the cards over and to arrange them in the correct order to tell a story. Order of cards and time taken were recorded. | False beliefs | 4 | 1. False-belief 2. Social-script (logical sequences) 3. Mechanical (physical cause-and-effect reasoning) 4. Capture (inhibitory suppression) | 96 |
| **Le Donne Cartoon task** | Le Donne, I., Attanasio, M., Bologna, A., Vagnetti, R., Masedu, F., Valenti, M., & Mazza, M. (2023). Autism and intention attribution test: a non-verbal evaluation with comic strips. *Annals of General Psychiatry*, *22*(1), 29. | Cartoon-like vignettes | Participants are shown three vignettes depicting an action or causal relation, followed by three alternative conclusions to the scenario. They must select the correct ending from the options provided. The endings include: (a) the correct conclusion, which is understandable if the participant grasps the protagonist’s intention; (b) a wrong ending that closely resembles the final vignette; and (c) a wrong ending that describes an unrelated everyday action. | Intentionality | 3 | Control; first order; second order | Only the first and second order: IA-CST = 9 |
| **Moore FBT** | Moore, R., Blackwood, N., Corcoran, R., Rowse, G., Kinderman, P., Bentall, R., & Howard, R. (2006). Misunderstanding the intentions of others: an exploratory study of the cognitive etiology of persecutory delusions in very late-onset schizophrenia-like psychosis. The American journal of geriatric psychiatry, 14(5), 410-418. | Black and white cartoon | Six stories were read aloud to participants, accompanied by cartoon drawings depicting each story's events. Participants were also asked questions to assess their memory of the stories. | False beliefs | 2 | First- and second- order | 6*2 = 12 |
| **Sarfati CST** | Sarfati Y, Hardy-Bayle M, Besche C, Widlocher D. Attribution of intentions to others in people with schizophrenia: a non-verbal exploration with comic strips. Schizophr Res. 1997;25:199–209. | Black and white comic | Three consecutive comics were given as the story, then choose a logical answers as an end among three options. | Intentionality | 1 | Attribution of intention | 28 30 (Kim et al., 2011) |
| **Schnell cartoon** | Schnell, K., Bluschke, S., Konradt, B., & Walter, H. (2011). Functional relations of empathy and mentalizing: an fMRI study on the neural basis of cognitive empathy. Neuroimage, 54(2), 1743-1754. | Black and white cartoon | Participants were instructed to judge picture to picture changes in visuospatial representations or affective states either from the first or from the third person perspectives. | False beliefs | 4 | 1st perspective visuospatial, 1st perspective affective, 3rd visuospatial, 3rd affective | 32*4 |
| **Stanford FBT** | Stanford, A. D., Messinger, J., Malaspina, D., & Corcoran, C. M. (2011). Theory of Mind in patients at clinical high risk for psychosis. Schizophrenia Research, 131(1–3), 11–17. https://doi.org/10.1016/j.schres.2011.06.005 | Black and white cartoon | Stories were first read to the subjects as they viewed the cartoons 1) Apple Task: Participants were asked whether an individual could know that an object had been moved in his absence.  2) Refrigerator Task: Participants were asked for the recognition of deception | False beliefs | 2 | First order, second order | 2 |
| **Walter CST** | Walter, H., Ciaramidaro, A., Adenzato, M., Vasic, N., Ardito, R. B., Erk, S., & Bara, B. G. (2009). Dysfunction of the social brain in schizophrenia is modulated by intention type: an fMRI study. Social cognitive and affective neuroscience, 4(2), 166-176. | Black and white comic | Comic strips consisted of three pictures shown for 3 seconds each, followed by a choice phase displaying three possible endings for 7 seconds. Each trial lasted 16 seconds. Participants selected the logical story ending by pressing one of three buttons as quickly as possible. | Intentionality | 1 | Attribution of intention | 4*11 = 44 |
| **Yoni Task** | Shamay-Tsoory, S. G., & Aharon-Peretz, J. (2007) Dissociable prefrontal networks for cognitive and affective theory of mind: A lesion study. Neuropsychologia, 45(13), 3054–3067 | A cartoon outline of a face (named Yoni) and coloured pictures | The task consists of 64 trials featuring a cartoon face named Yoni in the center, with four colored images in the corners. Participants must select the correct image based on a sentence at the top, which asks them to identify the object that "Yoni loves" (first-order affective Theory of Mind) or "Yoni is thinking about" (first-order cognitive Theory of Mind). | False beliefs | 3 | Cognitive, affective, physical (control) | 64 |

Note. CST indicate comic strip task; FBT, false belief task; PST, picture sequencing task.

# Supplementary Table 4. Meta-regression of theory of mind with static illustrations across psychiatric conditions compared to healthy controls, and between conditions.

| **Variables** | **k** | **Beta (95% CI)** | **SE** | **Qm** | **p value** |
| --- | --- | --- | --- | --- | --- |
| **ASD** |  |  |  |  |  |
| Age | 9 | 0.005 (-0.078 to 0.088) | 0.035 | 0.021 | 0.888 |
| Female proportion | 8 | -0.751 (-3.031 to 1.528) | 0.932 | 0.650 | 0.451 |
| Education | 4 | 0.308 (0.002 to 0.613) | 0.071 | 18.796 | **0.049** |
| IQ | 7 | 0.069 (0.001 to 0.138) | 0.027 | 6.742 | **0.048** |
| Publication year | 10 | 0.007 (-0.074 to 0.089) | 0.035 | 0.045 | 0.837 |
| Sample size | 10 | 0.002 (-0.002 to 0.006) | 0.002 | 1.276 | 0.291 |
| Quality assessment score | 10 | -0.083 (-0.516 to 0.350) | 0.188 | 0.194 | 0.671 |
| **Depression** |  |  |  |  |  |
| Age | 10 | 0.000 (-0.020 to 0.021) | 0.009 | 0.002 | 0.968 |
| Female proportion | 10 | 1.411 (-0.079 to 2.901) | 0.646 | 4.770 | 0.060 |
| Education | 4 | 0.096 (-0.286 to 0.478) | 0.089 | 1.176 | 0.391 |
| IQ | 7 | -0.004 (-0.075 to 0.067) | 0.028 | 0.019 | 0.896 |
| Publication year | 10 | 0.007 (-0.038 to 0.052) | 0.02 | 0.136 | 0.722 |
| Sample size | 10 | -0.003 (-0.019 to 0.013) | 0.007 | 0.231 | 0.643 |
| Quality assessment score | 10 | -0.120 (-0.314 to 0.074) | 0.084 | 2.049 | 0.190 |
| **Bipolar disorder** |  |  |  |  |  |
| Age | 10 | -0.012 (-0.070 to 0.046) | 0.025 | 0.214 | 0.656 |
| Female proportion | 10 | -1.243 (-6.970 to 4.483) | 2.483 | 0.251 | 0.630 |
| Education | 5 | -0.026 (-0.257 to 0.206) | 0.073 | 0.125 | 0.747 |
| IQ | 7 | 0.037 (-0.036 to 0.109) | 0.028 | 1.671 | 0.253 |
| Publication year | 10 | 0.053 (-0.007 to 0.112) | 0.026 | 4.187 | 0.075 |
| Sample size | 10 | 0.007 (-0.011 to 0.024) | 0.008 | 0.797 | 0.398 |
| Quality assessment score | 10 | 0.041 (-0.267 to 0.350) | 0.134 | 0.095 | 0.766 |
| **BPD** |  |  |  |  |  |
| Age | 4 | -0.060 (-0.356 to 0.237) | 0.069 | 0.752 | 0.477 |
| Female proportion | 4 | -1.571 (-20.385 to 17.243) | 4.373 | 0.129 | 0.754 |
| Education | 1 | / | / | / | / |
| IQ | 1 | / | / | / | / |
| Publication year | 4 | -0.043 (-0.199 to 0.114) | 0.036 | 1.366 | 0.363 |
| Sample size | 4 | 0.000 (-0.030 to 0.031) | 0.007 | 0.005 | 0.952 |
| Quality assessment score | 4 | -0.259 (-0.692 to 0.175) | 0.101 | 6.597 | 0.124 |
| **CHR** |  |  |  |  |  |
| Age | 11 | -0.030 (-0.100 to 0.040) | 0.031 | 0.931 | 0.360 |
| Female proportion | 11 | 0.008 (-1.756 to 1.773) | 0.78 | 0.001 | 0.992 |
| Education | 7 | -0.087 (-0.321 to 0.146) | 0.091 | 0.922 | 0.381 |
| IQ | 7 | -0.011 (-0.052 to 0.030) | 0.016 | 0.477 | 0.520 |
| Publication year | 11 | -0.029 (-0.065 to 0.007) | 0.016 | 3.355 | 0.100 |
| Sample size | 11 | 0.001 (-0.004 to 0.006) | 0.002 | 0.367 | 0.559 |
| Quality assessment score | 11 | -0.064 (-0.315 to 0.187) | 0.111 | 0.329 | 0.58 |
| **Early schizophrenia** |  |  |  |  |  |
| Age | 20 | -0.001 (-0.016 to 0.015) | 0.007 | 0.007 | 0.933 |
| Female proportion | 20 | -0.069 (-0.998 to 0.859) | 0.442 | 0.025 | 0.877 |
| Education | 16 | 0.026 (-0.170 to 0.223) | 0.091 | 0.083 | 0.778 |
| IQ | 14 | 0.013 (-0.026 to 0.052) | 0.018 | 0.56 | 0.469 |
| Publication year | 20 | 0.018 (-0.020 to 0.056) | 0.018 | 0.959 | 0.340 |
| Sample size | 20 | 0.003 (-0.003 to 0.009) | 0.003 | 1.247 | 0.279 |
| Quality assessment score | 20 | -0.048 (-0.189 to 0.094) | 0.067 | 0.498 | 0.490 |
| **FHR-S** |  |  |  |  |  |
| Age | 5 | -0.027 (-0.051 to -0.002) | 0.008 | 12.212 | **0.040** |
| Female proportion | 5 | -0.430 (-2.627 to 1.767) | 0.69 | 0.388 | 0.577 |
| Education | 4 | 0.080 (-0.485 to 0.644) | 0.131 | 0.371 | 0.605 |
| IQ | 4 | 0.165 (-0.120 to 0.449) | 0.066 | 6.204 | 0.130 |
| Publication year | 5 | 0.069 (-0.050 to 0.189) | 0.038 | 3.400 | 0.162 |
| Sample size | 5 | 0.001 (-0.001 to 0.003) | 0.001 | 2.156 | 0.238 |
| Quality assessment score | 5 | -0.428 (-1.838 to 0.982) | 0.443 | 0.933 | 0.405 |
| **Schizophrenia** |  |  |  |  |  |
| Age | 37 | -0.028 (-0.067 to 0.010) | 0.019 | 2.215 | 0.146 |
| Female proportion | 34 | 1.029 (-1.231 to 3.289) | 1.109 | 0.86 | 0.361 |
| Education | 21 | 0.364 (0.079 to 0.650) | 0.136 | 7.125 | **0.015** |
| IQ | 22 | 0.011 (-0.025 to 0.048) | 0.017 | 0.434 | 0.518 |
| Publication year | 37 | 0.027 (-0.004 to 0.058) | 0.015 | 3.166 | 0.084 |
| Sample size | 37 | 0.001 (0.000 to 0.003) | 0.001 | 4.461 | **0.042** |
| Quality assessment score | 37 | -0.001 (-0.146 to 0.144) | 0.071 | 0 | 0.993 |
| **CHR vs Early schizophrenia** |  |  |  |  |  |
| Age | 5 | -0.131 (-1.072 to 0.811) | 0.296 | 0.196 | 0.688 |
| Female proportion | 5 | -2.808 (-9.630 to 4.014) | 2.144 | 1.716 | 0.282 |
| Education | 4 | 1.572 (-1.245 to 4.389) | 0.655 | 5.765 | 0.138 |
| IQ | 2 | / |  |  |  |
| Publication year | 5 | -0.050 (-0.146 to 0.047) | 0.030 | 2.702 | 0.199 |
| Sample size | 5 | -0.008 (-0.045 to 0.028) | 0.011 | 0.513 | 0.526 |
| Quality assessment score | 5 | -0.350 (-0.690 to -0.010) | 0.107 | 10.755 | **0.046** |
| **Depression vs Schizophrenia** |  |  |  |  |  |
| Age | 5 | -0.022 (-0.926 to 0.883) | 0.284 | 0.006 | 0.944 |
| Female proportion | 5 | -0.511 (-2.589 to 3.612) | 0.974 | 0.276 | 0.636 |
| Education | 2 | / | / | / | / |
| IQ | 2 | / | / | / | **/** |
| Publication year | 5 | -0.013 (-0.082 to 0.056) | 0.022 | 0.377 | 0.583 |
| Sample size | 5 | 0.001 (-0.010 to 0.011) | 0.003 | 0.057 | 0.827 |
| Quality assessment score | 5 | 0.127 (-0.680 to 0.934) | 0.254 | 0.251 | 0.651 |

Note. ASD indicates autism spectrum disorders; BPD, borderline personality disorder; CHR, clinical high risk for psychosis; FHR-S, familial high risk for schizophrenia; IQ, intelligence quotient.

# Supplementary Table 5. Network heterogeneity and inconsistency

A) Heterogeneity assessment

| Outcome | No. of sample (comparisons) | No. of nodes (disorders) | Between study variance (τ²) | Proportion of total variance due to between studies differences (I^2^) | Judgement on heterogeneity |
| --- | --- | --- | --- | --- | --- |
| Overall | 92 (133) | 10 | 0.148 | 72.3% [66.4%, 77.2%] | Low-moderate |
| False belief | 36 (57) | 10 | 0.099 | 65.4% [51.2%; 75.4%] | Low-moderate |
| Intentionality | 45 (61) | 10 | 0.183 | 73.3% [64.2%; 80.1%] | Moderate-high |

B) Inconsistency assessment

| Outcome | Q statistics of design-by treatment interaction test (df) | P value of design-by treatment interaction test | Inconsistent comparisons of detachable comparisons (SIDE-test p <0.05) | Judgement on inconsistency |
| --- | --- | --- | --- | --- |
| Overall | 17.70 (21) | 0.668 | 1/19 | Little evidence of  inconsistency |
| False belief | 8.24 (11) | 0.692 | 0/13 | No evidence of  inconsistency |
| Intentionality | 6.51 (10) | 0.771 | 0/13 | No evidence of  inconsistency |

# Supplementary Table 6. Comparison of direct and indirect evidence using side (separating indirect from direct evidence) analysis

A) Overall Network Analysis

| comparison | k | prop | nma | direct | indir. | Diff | z | p-value |
| --- | --- | --- | --- | --- | --- | --- | --- | --- |
| ASD:Early schizophrenia | 2 | 0.26 | 0.2801 | -0.0123 | 0.3846 | -0.3969 | -1.08 | 0.2795 |
| ASD:HC | 10 | 0.91 | -0.5049 | -0.4056 | -1.4804 | 1.0748 | 2.27 | **0.0230** |
| ASD:Schizophrenia | 1 | 0.14 | 0.4548 | 0.1745 | 0.5005 | -0.326 | -0.74 | 0.4616 |
| Bipolar:Depression | 2 | 0.27 | 0.0996 | 0.1932 | 0.0645 | 0.1287 | 0.32 | 0.7501 |
| Bipolar:Early schizophrenia | 1 | 0.13 | 0.459 | 0.4488 | 0.4606 | -0.0117 | -0.02 | 0.9808 |
| Bipolar:HC | 10 | 0.87 | -0.326 | -0.347 | -0.1797 | -0.1674 | -0.4 | 0.6868 |
| Bipolar:Schizophrenia | 1 | 0.08 | 0.6337 | 0.6736 | 0.6301 | 0.0435 | 0.08 | 0.9382 |
| BPD:Depression | 1 | 0.29 | -0.1861 | -0.7236 | 0.0327 | -0.7563 | -1.41 | 0.1597 |
| BPD:HC | 4 | 0.93 | -0.6117 | -0.5387 | -1.5978 | 1.0591 | 1.23 | 0.2203 |
| CHR:Early schizophrenia | 5 | 0.52 | 0.377 | 0.4343 | 0.3157 | 0.1186 | 0.39 | 0.6989 |
| CHR:HC | 11 | 0.9 | -0.4081 | -0.4485 | -0.0451 | -0.4034 | -0.88 | 0.3775 |
| Depression:Early schizophrenia | 2 | 0.23 | 0.3594 | 0.2967 | 0.3786 | -0.0819 | -0.22 | 0.8252 |
| Depression:HC | 10 | 0.77 | -0.4256 | -0.4272 | -0.4201 | -0.0071 | -0.02 | 0.9815 |
| Depression:Schizophrenia | 5 | 0.43 | 0.5341 | 0.567 | 0.5095 | 0.0575 | 0.2 | 0.8394 |
| Early schizophrenia:HC | 20 | 0.9 | -0.785 | -0.7948 | -0.7013 | -0.0935 | -0.29 | 0.7733 |
| Early schizophrenia:Schizophrenia relatives | 1 | 0.2 | -0.2182 | -0.3462 | -0.1857 | -0.1605 | -0.32 | 0.7469 |
| Schizophrenia relatives:HC | 5 | 0.84 | -0.5668 | -0.4527 | -1.178 | 0.7253 | 1.47 | 0.1414 |
| Schizophrenia:HC | 37 | 0.94 | -0.9597 | -0.9477 | -1.1393 | 0.1917 | 0.61 | 0.5396 |
| Schizophrenia:Schizophrenia relatives | 2 | 0.38 | -0.3929 | -0.1239 | -0.5598 | 0.4359 | 1.13 | 0.2595 |

B) False Belief

| comparison | k | prop | nma | direct | indir. | Diff | z | p-value |
| --- | --- | --- | --- | --- | --- | --- | --- | --- |
| ASD:Early schizophrenia | 2 | 0.8 | 0.1584 | -0.0143 | 0.8636 | -0.8779 | -1.43 | 0.154 |
| ASD:HC | 2 | 0.79 | -0.4587 | -0.2809 | -1.1377 | 0.8568 | 1.42 | 0.1569 |
| Bipolar:Depression | 1 | 0.28 | -0.0417 | 0.169 | -0.1221 | 0.2911 | 0.63 | 0.531 |
| Bipolar:Early schizophrenia | 1 | 0.24 | 0.2589 | 0.4488 | 0.1977 | 0.2511 | 0.55 | 0.5821 |
| Bipolar:HC | 6 | 0.93 | -0.3582 | -0.4046 | 0.2559 | -0.6605 | -1.07 | 0.2831 |
| Depression:Early schizophrenia | 2 | 0.41 | 0.3006 | 0.2964 | 0.3035 | -0.0071 | -0.02 | 0.9848 |
| Depression:HC | 6 | 0.8 | -0.3165 | -0.3212 | -0.2973 | -0.024 | -0.06 | 0.9493 |
| Depression:Schizophrenia | 3 | 0.51 | 0.4719 | 0.5539 | 0.3852 | 0.1687 | 0.52 | 0.6061 |
| Early schizophrenia:HC | 9 | 0.92 | -0.6171 | -0.6301 | -0.4761 | -0.1539 | -0.33 | 0.7408 |
| Early schizophrenia:Schizophrenia relatives | 1 | 0.38 | -0.4186 | -0.3462 | -0.4633 | 0.1171 | 0.24 | 0.8111 |
| Schizophrenia relatives:HC | 2 | 0.77 | -0.1985 | 0.0044 | -0.892 | 0.8964 | 1.73 | 0.0842 |
| Schizophrenia:HC | 16 | 0.95 | -0.7885 | -0.7688 | -1.2014 | 0.4327 | 0.9 | 0.3677 |
| Schizophrenia:Schizophrenia relatives | 1 | 0.5 | -0.5899 | -0.3008 | -0.8753 | 0.5745 | 1.27 | 0.2035 |

C) Intentionality

| comparison | k | prop | nma | direct | indir. | Diff | z | p-value |
| --- | --- | --- | --- | --- | --- | --- | --- | --- |
| ASD:HC | 6 | 0.94 | -0.4354 | -0.3481 | -1.8378 | 1.4897 | 1.76 | 0.0786 |
| ASD:Schizophrenia | 1 | 0.25 | 0.5923 | 0.1745 | 0.7294 | -0.5549 | -1.07 | 0.286 |
| Bipolar:Depression | 1 | 0.34 | 0.3026 | 0.2264 | 0.3417 | -0.1153 | -0.17 | 0.8678 |
| Bipolar:HC | 3 | 0.76 | -0.3576 | -0.3448 | -0.3983 | 0.0535 | 0.09 | 0.9275 |
| Bipolar:Schizophrenia | 1 | 0.22 | 0.6701 | 0.6736 | 0.6691 | 0.0045 | 0.01 | 0.9945 |
| CHR:Early schizophrenia | 4 | 0.66 | 0.4127 | 0.4697 | 0.3037 | 0.166 | 0.38 | 0.7075 |
| CHR:HC | 7 | 0.92 | -0.4953 | -0.5519 | 0.1356 | -0.6874 | -1.04 | 0.3005 |
| Depression:HC | 3 | 0.72 | -0.6601 | -0.7535 | -0.4243 | -0.3292 | -0.6 | 0.5509 |
| Depression:Schizophrenia | 2 | 0.43 | 0.3676 | 0.5949 | 0.1943 | 0.4006 | 0.76 | 0.4487 |
| Early schizophrenia:HC | 9 | 0.95 | -0.9079 | -0.9283 | -0.5223 | -0.406 | -0.54 | 0.5926 |
| Schizophrenia relatives:HC | 3 | 0.92 | -0.862 | -0.8209 | -1.3193 | 0.4983 | 0.5 | 0.6147 |
| Schizophrenia:HC | 17 | 0.92 | -1.0277 | -1.0251 | -1.0567 | 0.0316 | 0.07 | 0.9423 |
| Schizophrenia:Schizophrenia relatives | 1 | 0.31 | -0.1657 | 0.1511 | -0.3065 | 0.4576 | 0.73 | 0.4642 |

Note. k indicates number of direct comparisons; prop, proportion of direct evidence; nma, network meta-analysis estimate; direct, effect size from direct comparisons; indir, effect size from indirect comparisons; Diff, direct minus indirect estimates.

# Supplementary Table 7. Confidence in the evidence evaluated using the Confidence in Network Meta-Analysis (CINeMA) framework.

| Comparison | k | Within-study bias | Reporting bias | Indirectness | Imprecision | Heterogeneity | Incoherence | Confidence rating | Reason(s) for downgrading |
| --- | --- | --- | --- | --- | --- | --- | --- | --- | --- |
| ASD:Early schizophrenia | 2 | Some concerns | Some concerns | No concerns | Some concerns | Some concerns | No concerns | Low | [Within-study bias, Reporting bias, Imprecision, Heterogeneity] |
| ASD:HC | 10 | Some concerns | Low risk | No concerns | No concerns | Some concerns | Some concerns | Low | [Within-study bias, Heterogeneity, Incoherence] |
| ASD:Schizophrenia | 1 | Some concerns | Low risk | No concerns | No concerns | Major concerns | No concerns | Low | [Within-study bias, Heterogeneity] |
| BPD:Depression | 1 | No concerns | Low risk | No concerns | Major concerns | No concerns | No concerns | Low | [Imprecision] |
| BPD:HC | 4 | No concerns | Low risk | No concerns | No concerns | Some concerns | No concerns | Moderate | [Heterogeneity] |
| Bipolar:Depression | 2 | No concerns | Low risk | No concerns | Some concerns | Some concerns | No concerns | Moderate | [Imprecision, Heterogeneity] |
| Bipolar:Early schizophrenia | 1 | No concerns | Some concerns | No concerns | No concerns | Some concerns | No concerns | Moderate | [Reporting bias, Heterogeneity] |
| Bipolar:HC | 10 | No concerns | Low risk | No concerns | Some concerns | Some concerns | No concerns | Moderate | [Imprecision, Heterogeneity] |
| Bipolar:Schizophrenia | 1 | Some concerns | Low risk | No concerns | No concerns | Some concerns | No concerns | Moderate | [Within-study bias, Heterogeneity] |
| CHR:Early schizophrenia | 5 | No concerns | Some concerns | No concerns | No concerns | Major concerns | No concerns | Low | [Reporting bias, Heterogeneity] |
| CHR:HC | 11 | No concerns | Low risk | No concerns | No concerns | Major concerns | No concerns | Low | [Heterogeneity] |
| Depression:Early schizophrenia | 2 | No concerns | Some concerns | No concerns | No concerns | Major concerns | No concerns | Low | [Reporting bias, Heterogeneity] |
| Depression:HC | 10 | Some concerns | Low risk | No concerns | No concerns | Major concerns | No concerns | Low | [Within-study bias, Heterogeneity] |
| Depression:Schizophrenia | 5 | Some concerns | Low risk | No concerns | No concerns | Some concerns | No concerns | Moderate | [Within-study bias, Heterogeneity] |
| Early schizophrenia:HC | 20 | No concerns | Some concerns | No concerns | No concerns | No concerns | No concerns | Moderate | [Reporting bias] |
| Early schizophrenia:Schizophrenia relatives | 1 | Some concerns | Some concerns | No concerns | Some concerns | Some concerns | No concerns | Low | [Within-study bias, Reporting bias, Imprecision, Heterogeneity] |
| HC:OCD | 3 | Some concerns | Low risk | No concerns | No concerns | Some concerns | No concerns | Moderate | [Within-study bias, Heterogeneity] |
| HC:Schizophrenia | 37 | Some concerns | Low risk | No concerns | No concerns | No concerns | No concerns | Moderate | [Within-study bias] |
| HC:Schizophrenia relatives | 5 | Some concerns | Some concerns | No concerns | No concerns | Some concerns | No concerns | Low | [Within-study bias, Reporting bias, Heterogeneity] |
| Schizophrenia:Schizophrenia relatives | 2 | Some concerns | Some concerns | No concerns | No concerns | Major concerns | No concerns | Low | [Within-study bias, Reporting bias, Heterogeneity] |
| ASD:BPD | 0 | Some concerns | Low risk | No concerns | Major concerns | No concerns | No concerns | Low | [Within-study bias, Imprecision] |
| ASD:Bipolar | 0 | Some concerns | Low risk | No concerns | Some concerns | Some concerns | No concerns | Low | [Within-study bias, Imprecision, Heterogeneity] |
| ASD:CHR | 0 | Some concerns | Low risk | No concerns | Some concerns | Some concerns | No concerns | Low | [Within-study bias, Imprecision, Heterogeneity] |
| ASD:Depression | 0 | Some concerns | Low risk | No concerns | Major concerns | No concerns | No concerns | Low | [Within-study bias, Imprecision] |
| ASD:OCD | 0 | Some concerns | Low risk | No concerns | Major concerns | No concerns | No concerns | Low | [Within-study bias, Imprecision] |
| ASD:Schizophrenia relatives | 0 | Some concerns | Some concerns | No concerns | Major concerns | No concerns | No concerns | Low | [Within-study bias, Reporting bias, Imprecision] |
| Bipolar:BPD | 0 | No concerns | Low risk | No concerns | Some concerns | Some concerns | No concerns | Moderate | [Imprecision, Heterogeneity] |
| BPD:CHR | 0 | No concerns | Low risk | No concerns | Major concerns | No concerns | No concerns | Low | [Imprecision] |
| BPD:Early schizophrenia | 0 | No concerns | Some concerns | No concerns | Some concerns | Some concerns | No concerns | Low | [Reporting bias, Imprecision, Heterogeneity] |
| BPD:OCD | 0 | No concerns | Low risk | No concerns | Major concerns | No concerns | No concerns | Low | [Imprecision] |
| BPD:Schizophrenia | 0 | No concerns | Low risk | No concerns | Some concerns | Some concerns | No concerns | Moderate | [Imprecision, Heterogeneity] |
| BPD:Schizophrenia relatives | 0 | No concerns | Some concerns | No concerns | Major concerns | No concerns | No concerns | Low | [Reporting bias, Imprecision] |
| Bipolar:CHR | 0 | No concerns | Low risk | No concerns | Some concerns | Some concerns | No concerns | Moderate | [Imprecision, Heterogeneity] |
| Bipolar:OCD | 0 | No concerns | Low risk | No concerns | Some concerns | Some concerns | No concerns | Moderate | [Imprecision, Heterogeneity] |
| Bipolar:Schizophrenia relatives | 0 | Some concerns | Some concerns | No concerns | Some concerns | Some concerns | No concerns | Low | [Within-study bias, Reporting bias, Imprecision, Heterogeneity] |
| CHR:Depression | 0 | No concerns | Low risk | No concerns | Major concerns | No concerns | No concerns | Low | [Imprecision] |
| CHR:OCD | 0 | Some concerns | Low risk | No concerns | Major concerns | No concerns | No concerns | Low | [Within-study bias, Imprecision] |
| CHR:Schizophrenia | 0 | Some concerns | Low risk | No concerns | No concerns | Some concerns | No concerns | Moderate | [Within-study bias, Heterogeneity] |
| CHR:Schizophrenia relatives | 0 | Some concerns | Some concerns | No concerns | Some concerns | Some concerns | No concerns | Low | [Within-study bias, Reporting bias, Imprecision, Heterogeneity] |
| Depression:OCD | 0 | Some concerns | Low risk | No concerns | Major concerns | No concerns | No concerns | Low | [Within-study bias, Imprecision] |
| Depression:Schizophrenia relatives | 0 | Some concerns | Some concerns | No concerns | Major concerns | No concerns | No concerns | Low | [Within-study bias, Reporting bias, Imprecision] |
| Early schizophrenia:OCD | 0 | Some concerns | Some concerns | No concerns | Major concerns | No concerns | No concerns | Low | [Within-study bias, Reporting bias, Imprecision] |
| Early schizophrenia:Schizophrenia | 0 | Some concerns | Some concerns | No concerns | Some concerns | Some concerns | No concerns | Low | [Within-study bias, Reporting bias, Imprecision, Heterogeneity] |
| OCD:Schizophrenia | 0 | Some concerns | Low risk | No concerns | Some concerns | Some concerns | No concerns | Low | [Within-study bias, Imprecision, Heterogeneity] |
| OCD:Schizophrenia relatives | 0 | Some concerns | Some concerns | No concerns | Major concerns | No concerns | No concerns | Low | [Within-study bias, Reporting bias, Imprecision] |

# Supplementary Table 8. Ceiling effects across mentalizing tasks and conditions

| Task | Condition | No. of sample reported raw scores | No. of sample being ceiling effect | Percentage of studies being ceiling effect | Mean scores in percentages |
| --- | --- | --- | --- | --- | --- |
| Brune PST | Overall | 41 | 28 | 68.3% | 84.7% |
|  | HC | 19 | 18 | 94.7% | 89.0% |
|  | Clinical groups | 22 | 10 | 45.5% | 81.0% |
|  | ASD | 2 | 2 | 100% | 82.4% |
|  | Bipolar disorder | 3 | 1 | 33.3% | 80.9% |
|  | BPD | 1 | 1 | 100% | 84.4% |
|  | CHR | 4 | 4 | 100% | 92.9% |
|  | Depression | 1 | 0 | 0% | 78.0% |
|  | Early schizophrenia | 3 | 2 | 66.6% | 88.0% |
|  | FHR-S | 2 | 0 | 0% | 76.8% |
|  | Schizophrenia | 6 | 0 | 0% | 70.5% |
| Brunet/ Sarfati CST | Overall | 34 | 20 | 58.8% | 82.1% |
|  | HC | 15 | 13 | 86.7% | 88.5% |
|  | Clinical groups | 19 | 7 | 36.8% | 77.1% |
|  | ASD | 3 | 0 | 0% | 68.7% |
|  | CHR | 3 | 2 | 66.7% | 84.2% |
|  | Depression | 1 | 1 | 100% | 81.1% |
|  | Early schizophrenia | 4 | 2 | 50% | 79.9% |
|  | FHR-S | 1 | 0 | 0% | 61.7% |
|  | Schizophrenia | 7 | 2 | 28.6% | 77.8% |
| Langdon PST | Overall | 35 | 23 | 65.7% | 80.2% |
|  | HC | 16 | 15 | 93.8% | 87.4% |
|  | Clinical groups | 19 | 8 | 42.1% | 74.1% |
|  | ASD | 1 | 1 | 100% | 81.9% |
|  | Bipolar disorder | 1 | 0 | 0% | 70.6% |
|  | CHR | 1 | 1 | 100% | 83.8% |
|  | Depression | 1 | 1 | 100% | 83.3% |
|  | Early schizophrenia | 4 | 2 | 50% | 77.8% |
|  | FHR-B | 1 | 1 | 100% | 81.5% |
|  | FHR-S | 1 | 1 | 100% | 84.0% |
|  | Schizophrenia | 9 | 1 | 11.1% | 68.0% |
| Yoni task | Overall | 20 | 13 | 65.0% | 80.2% |
|  | HC | 8 | 7 | 87.5% | 85.8% |
|  | Clinical groups | 12 | 6 | 50.0% | 76.5% |
|  | ASD | 1 | 1 | 100% | 83.3% |
|  | Bipolar | 3 | 2 | 66.7% | 82.5% |
|  | Depression | 1 | 0 | 0% | 70.2% |
|  | Early schizophrenia | 3 | 2 | 66.7% | 75.2% |
|  | FHR-S | 1 | 1 | 100% | 87.2% |
|  | OCD | 1 | 0 | 0% | 73.9% |
|  | Schizophrenia | 2 | 0 | 0% | 64.9% |
| Happé Cartoon task | Overall | 12 | 4 | 33.3% | 77.9% |
|  | HC | 6 | 3 | 50.0% | 85.2% |
|  | Clinical groups | 6 | 1 | 16.7% | 70.6% |
|  | Anorexia nervosa | 2 | 0 | 0% | 61.1% |
|  | ASD | 1 | 1 | 100% | 82.4% |
|  | CHR | 1 | 0 | 0% | 77.3% |
|  | Schizophrenia | 2 | 0 | 0% | 70.9% |

Note. Ceiling effects were defined as mean performance ≥80% of maximum possible score. Mean scores are presented as percentage of maximum possible score. ASD indicates autism spectrum disorder; BPD, borderline personality disorder; CHR, clinical high risk for psychosis; FHR-B, familial high risk for bipolar disorder; FHR-S, familial high risk for schizophrenia; OCD, obsessive compulsive disorder; CST, comic strip task; PST, picture sequencing task.

# Supplementary Figure 1. Forest plots of theory of mind with static illustrations across psychiatric conditions compared to healthy controls, and between conditions.

1. ASD vs HC


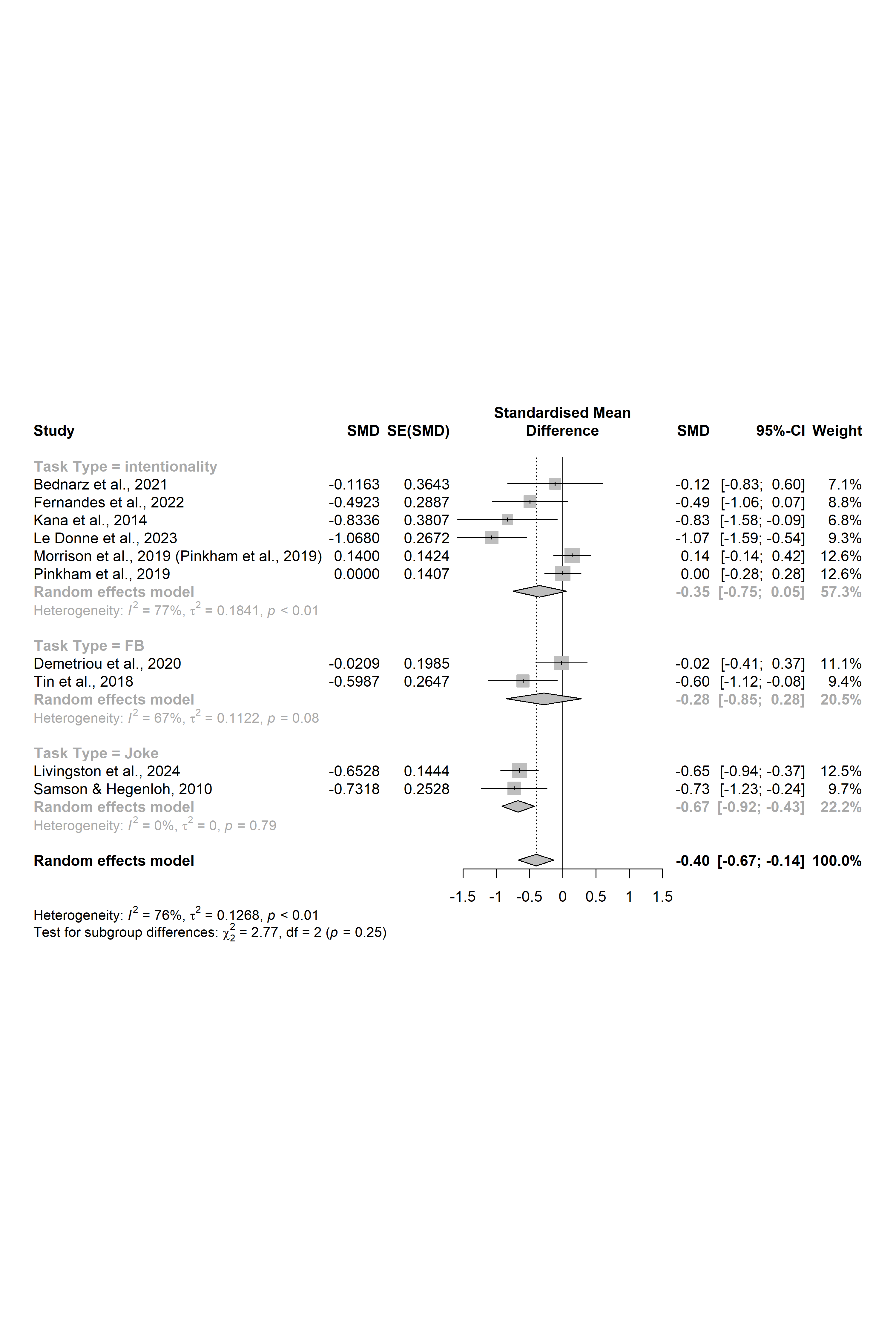


1. Bipolar disorder vs HC


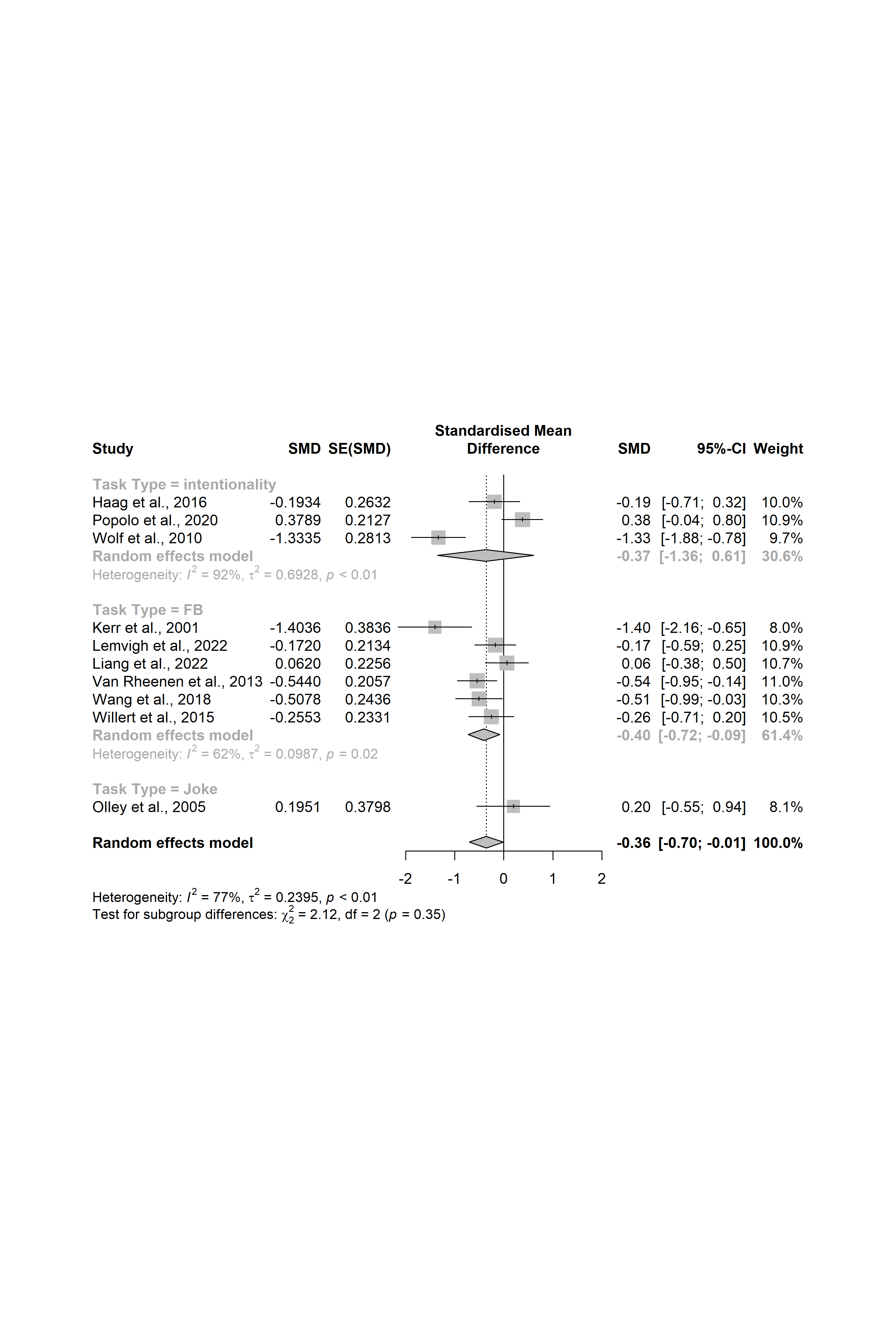


1. BPD vs HC


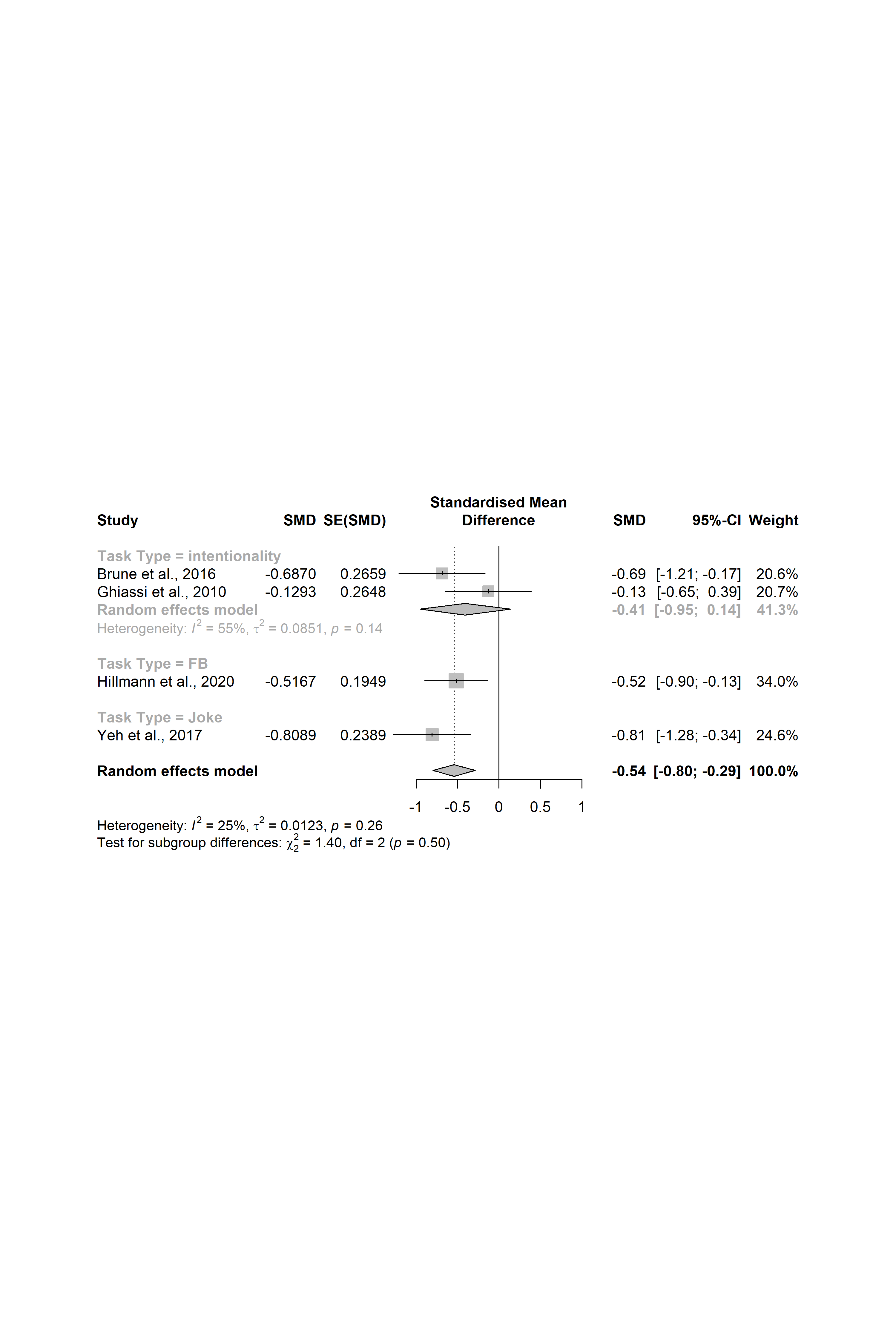


1. CHR vs HC


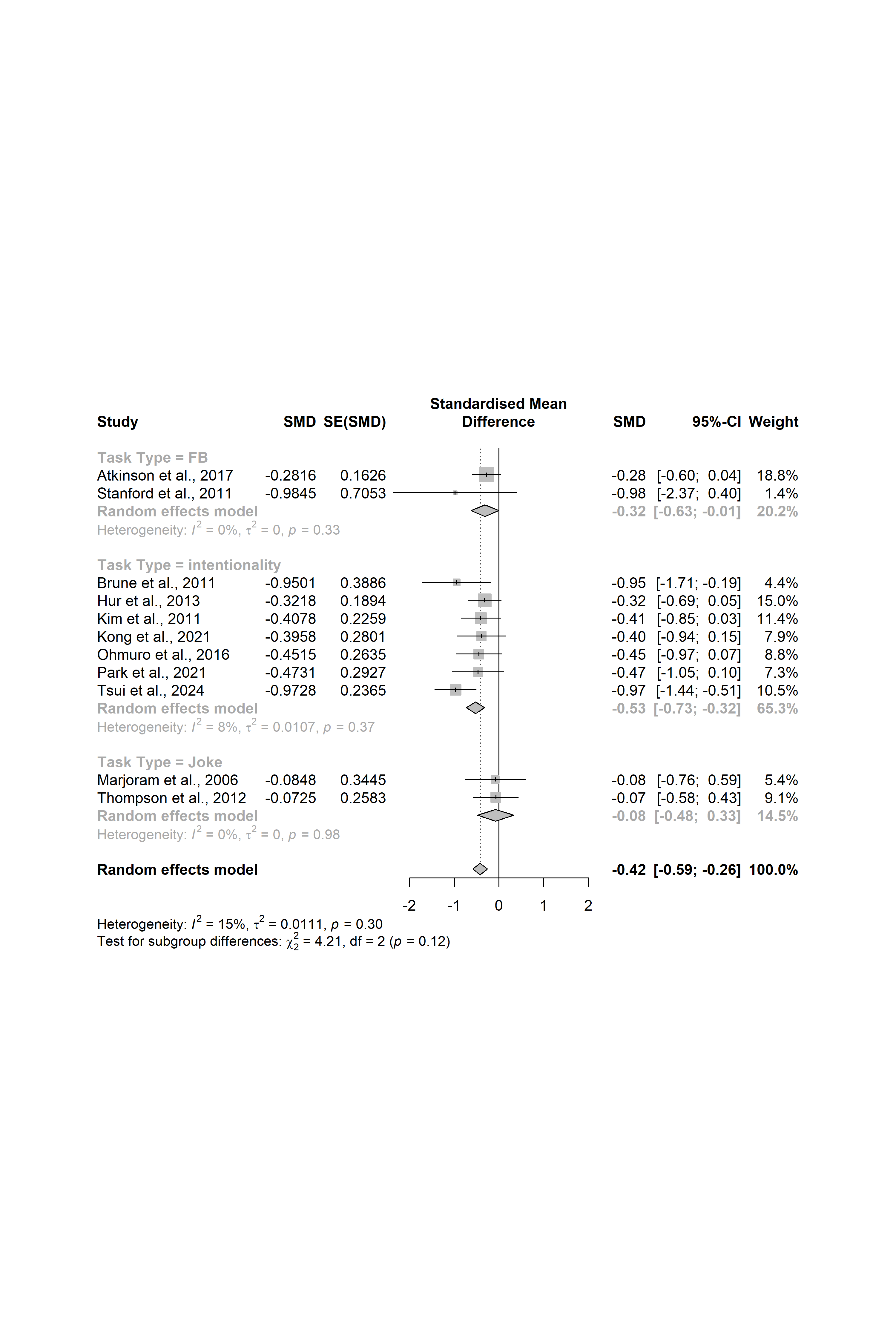


1. Depression vs HC


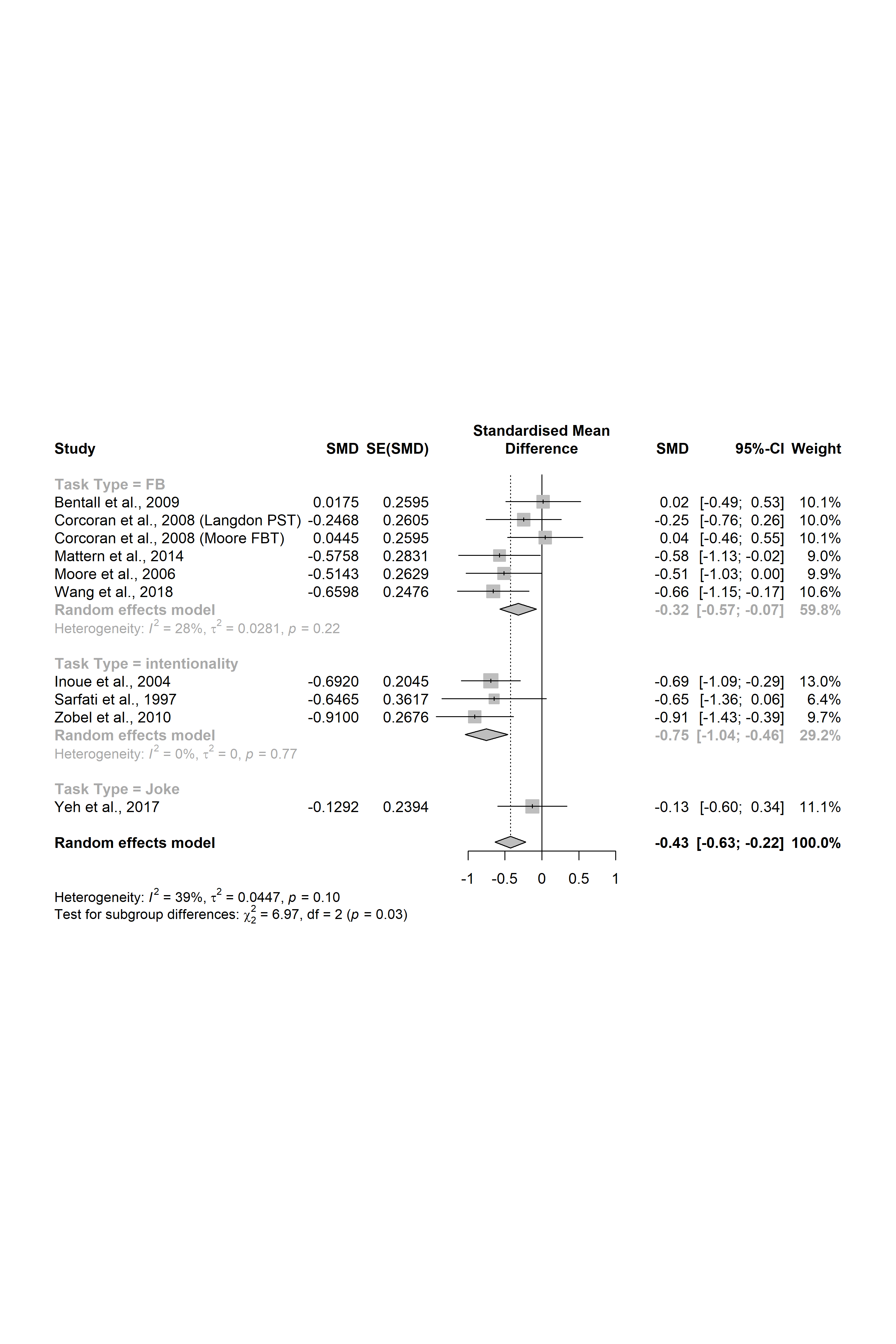


1. Early schizophrenia vs HC


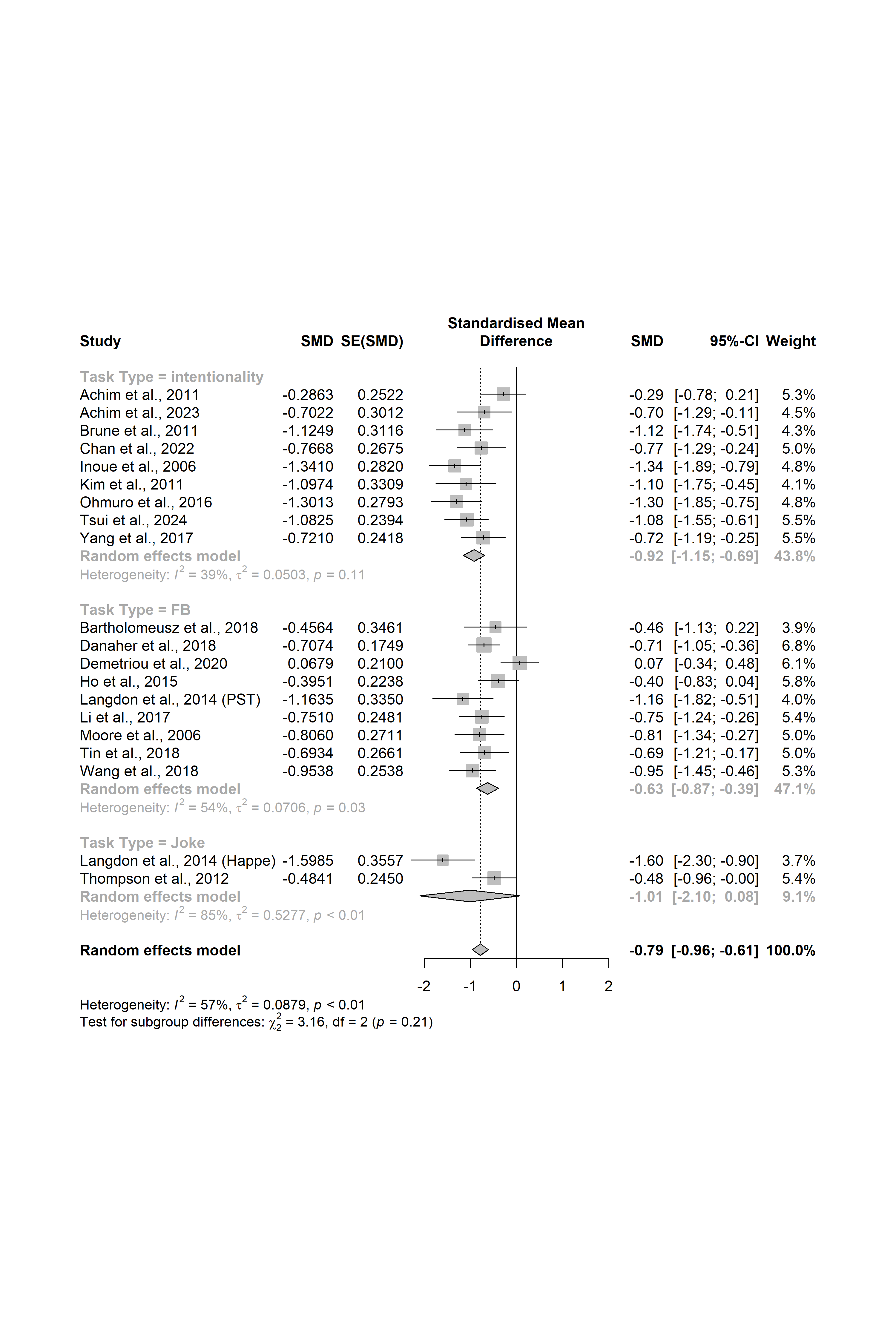


1. FHR-S vs HC


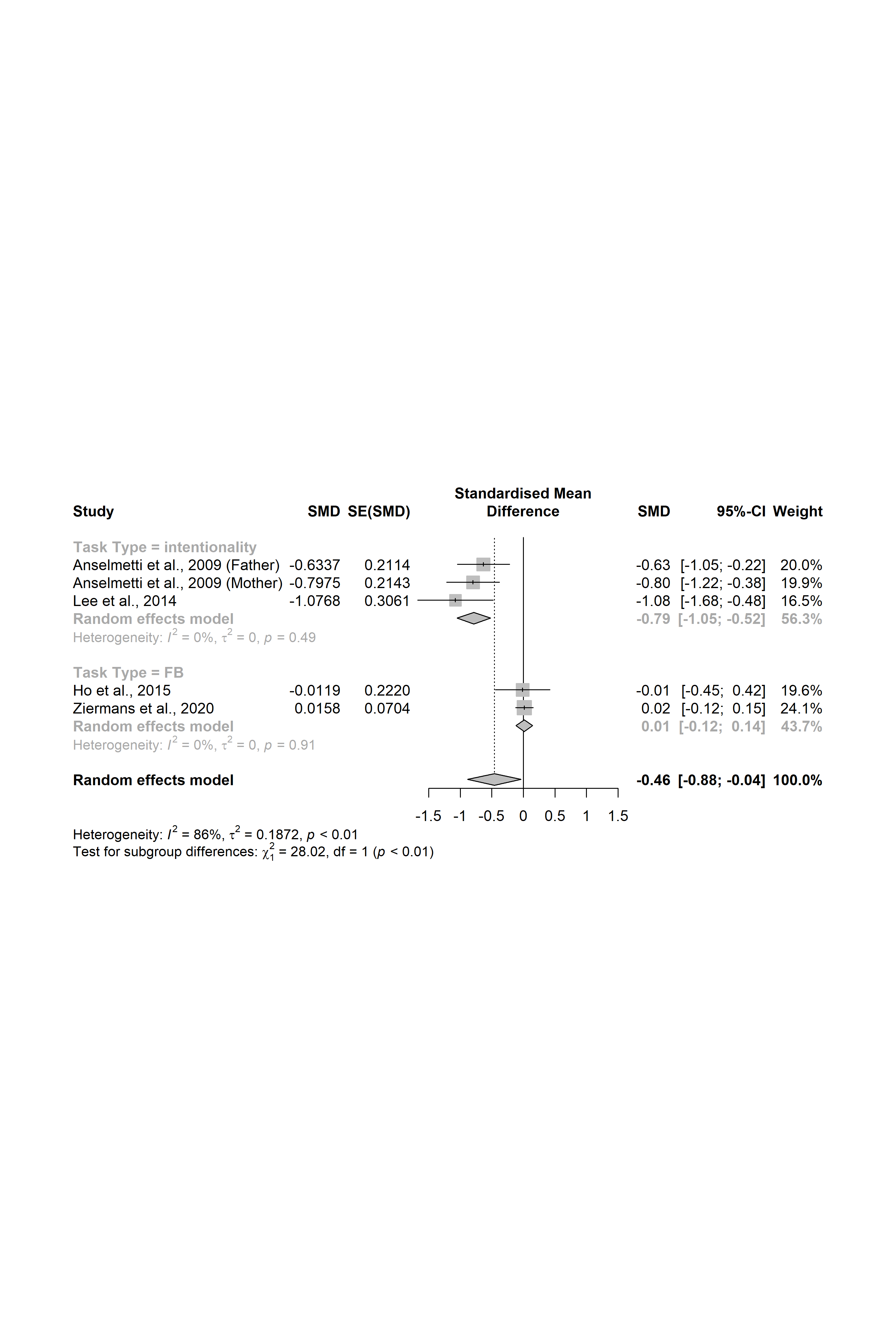


1. OCD vs HC


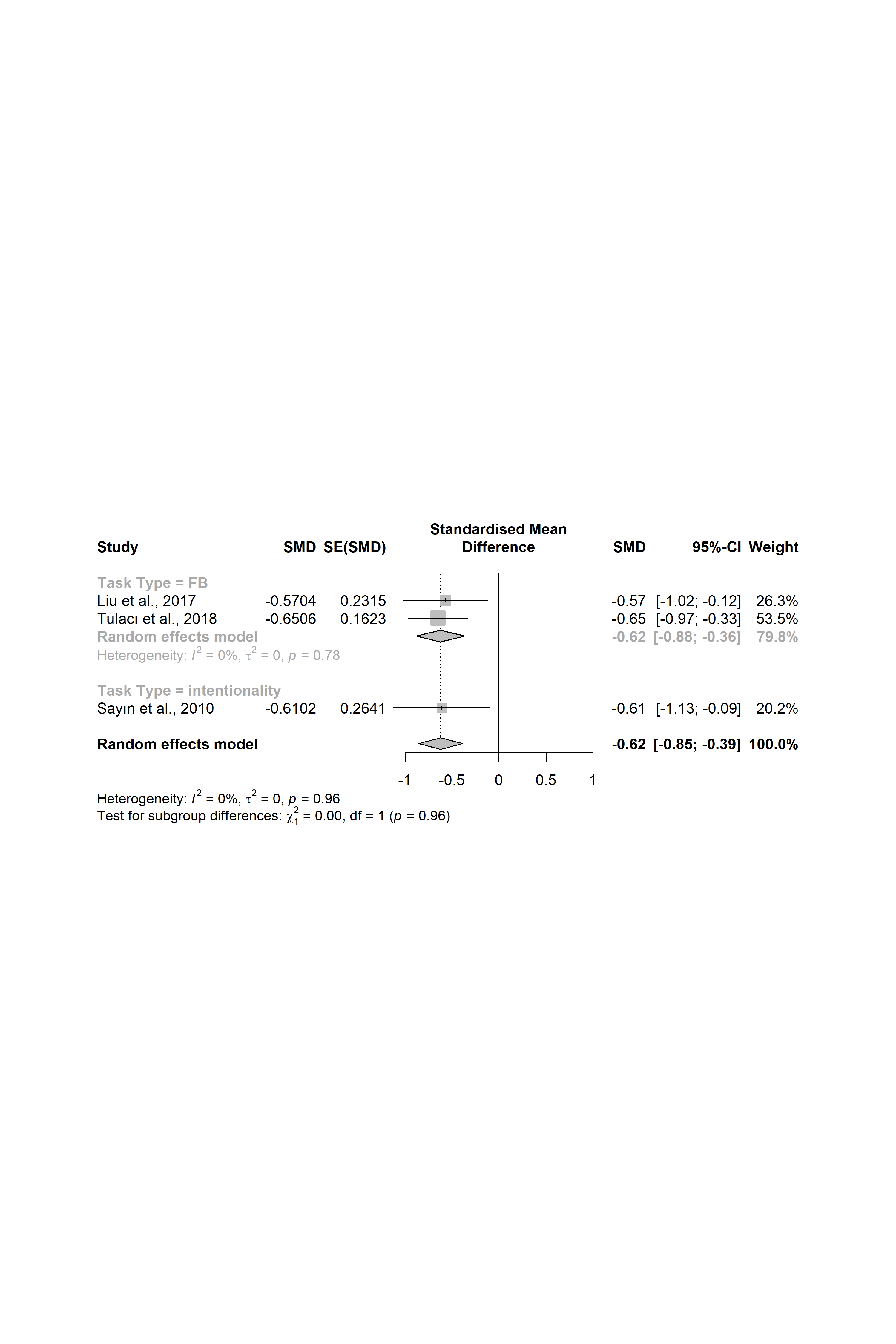


1. Schizophrenia vs HC


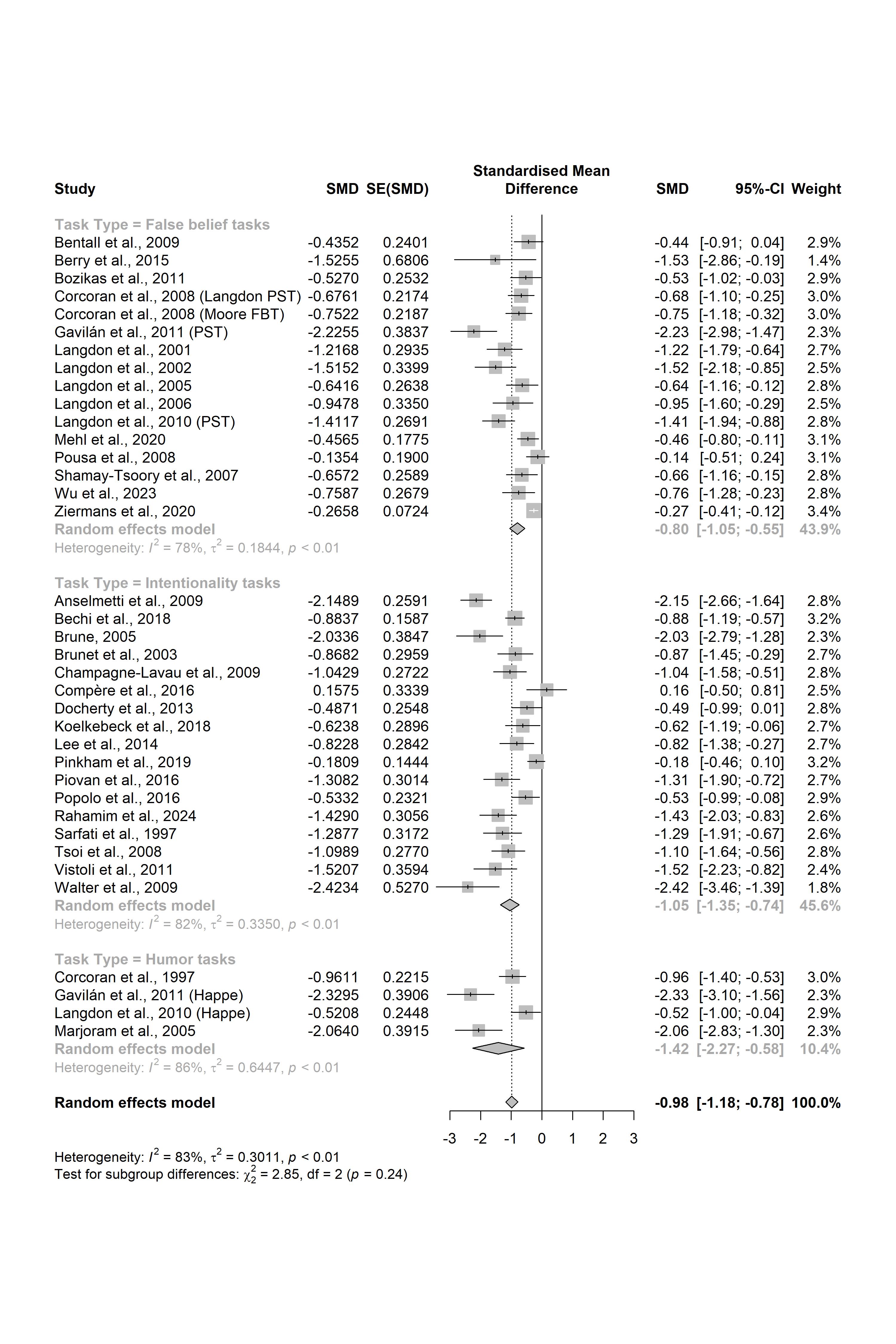


1. Anorexia Nervosa vs HC


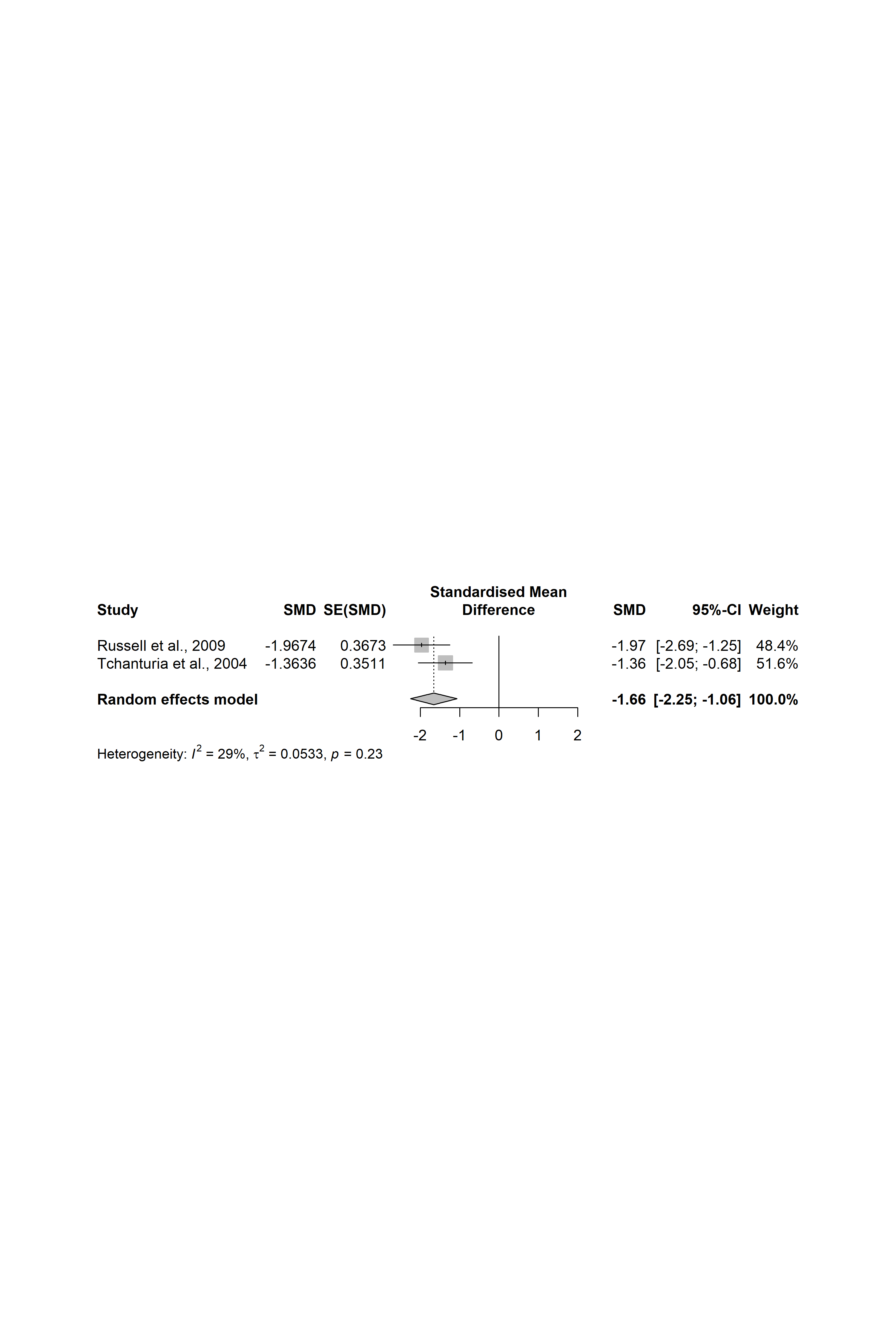


1. FHR-B vs HC


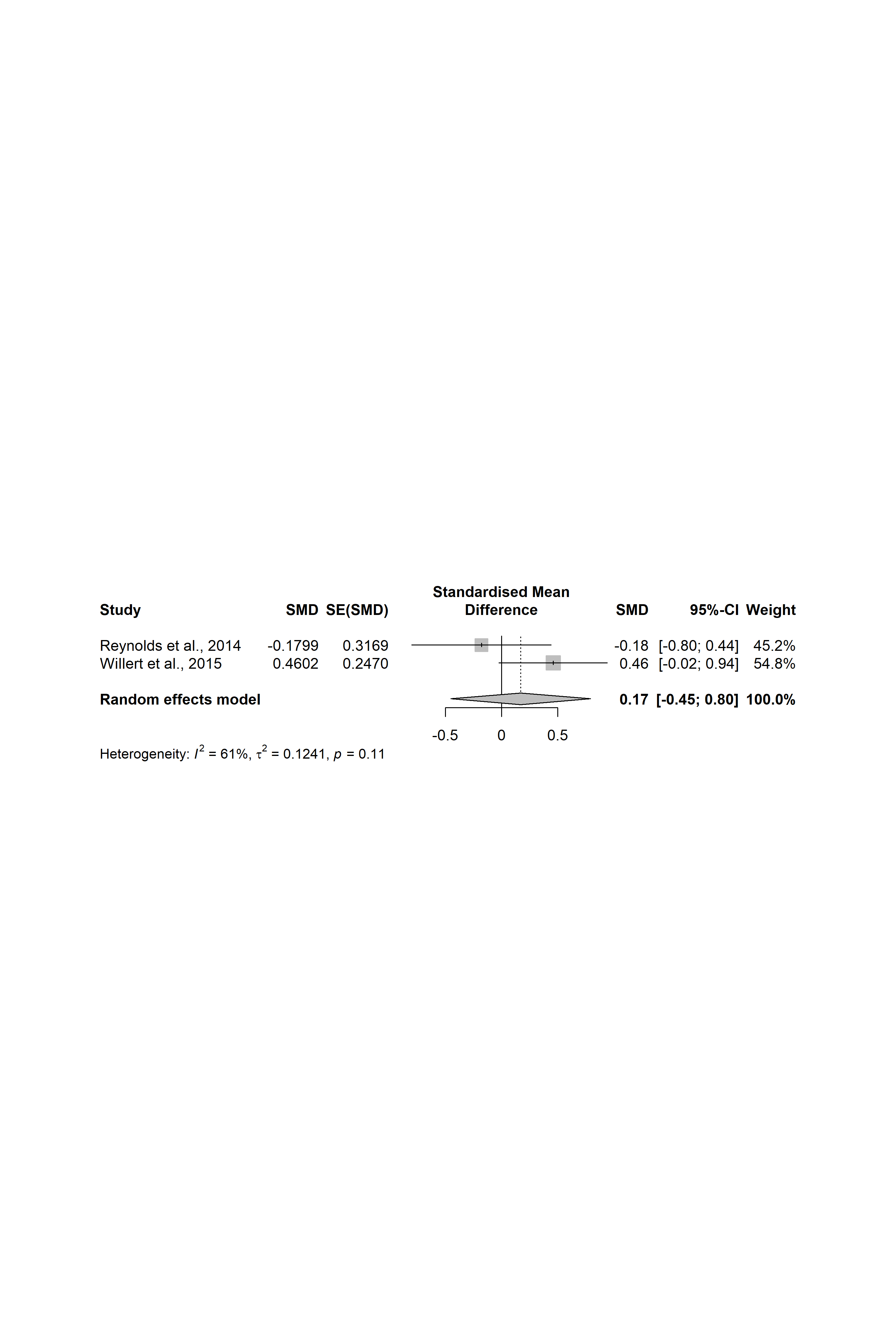


1. ASD vs Early schizophrenia


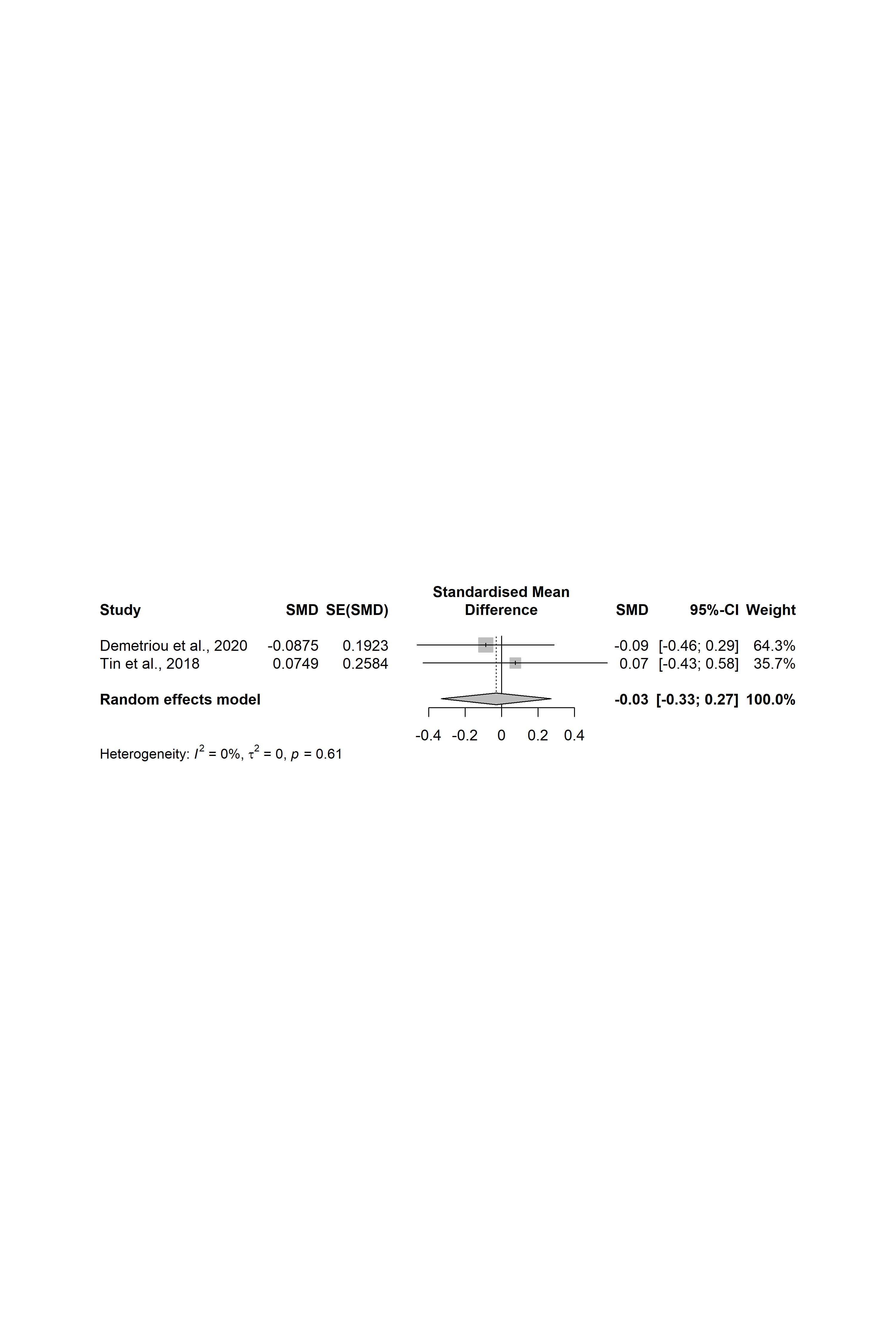


1. Bipolar disorder vs Depression


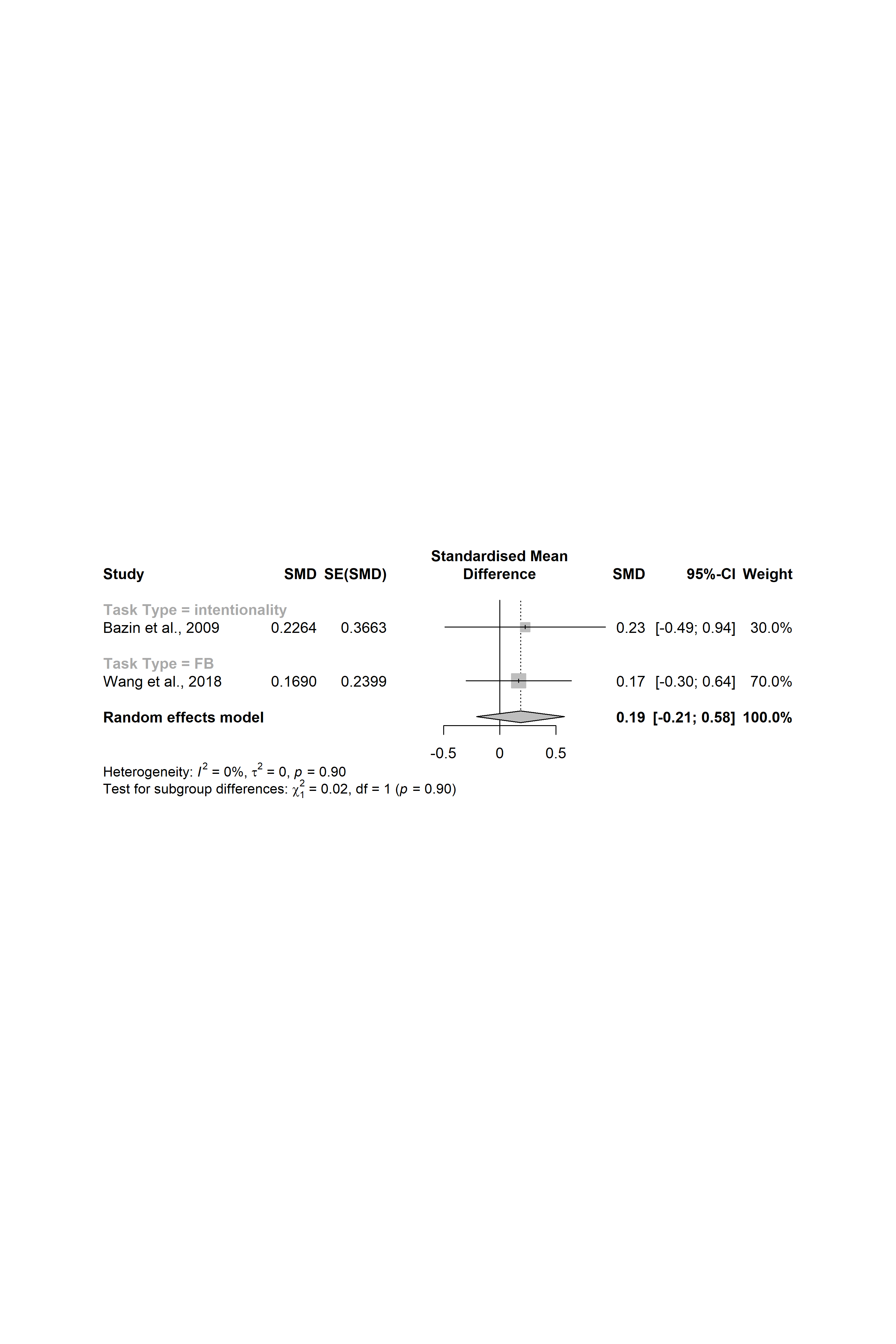


1. CHR vs Early schizophrenia


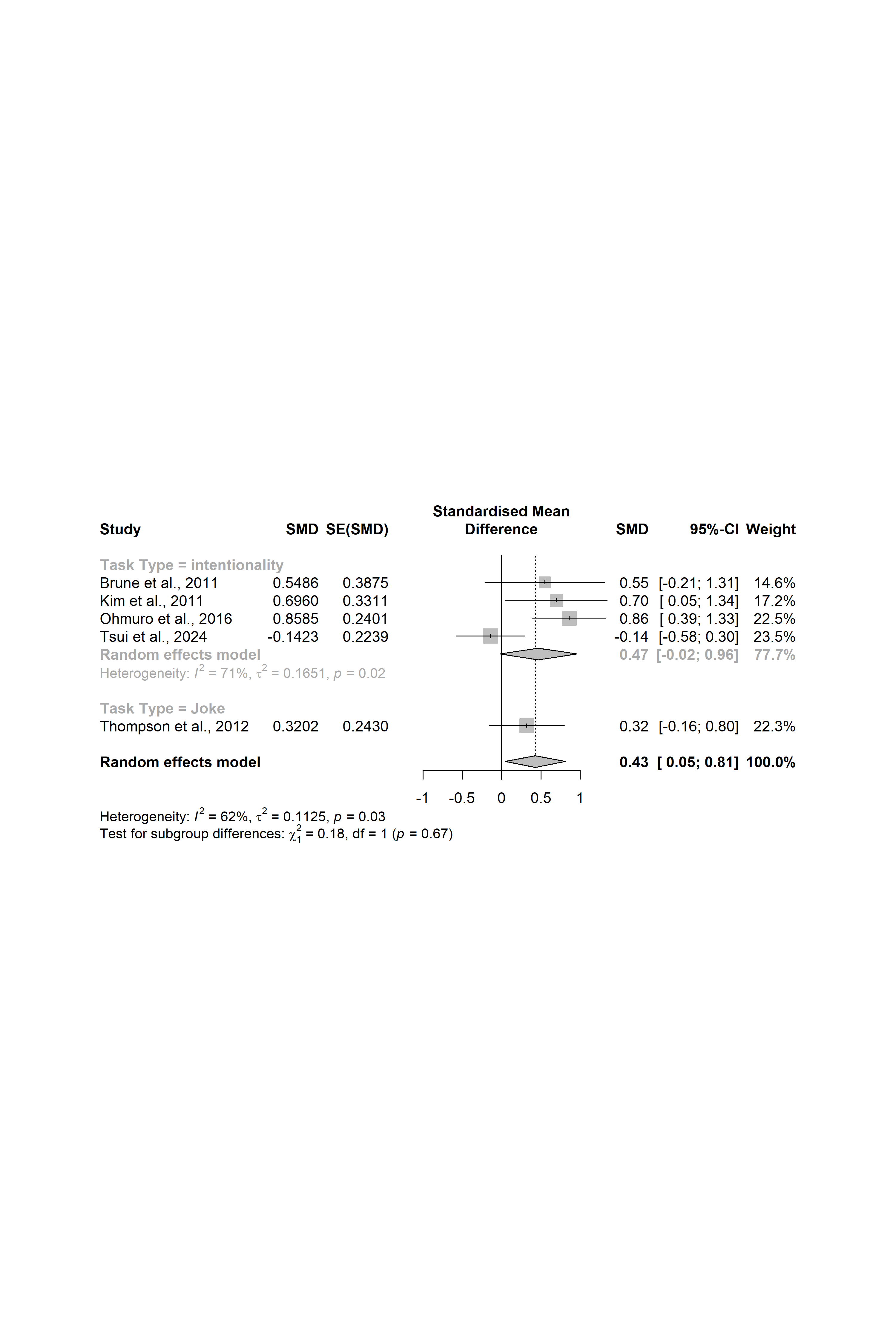


1. Depression vs Early schizophrenia


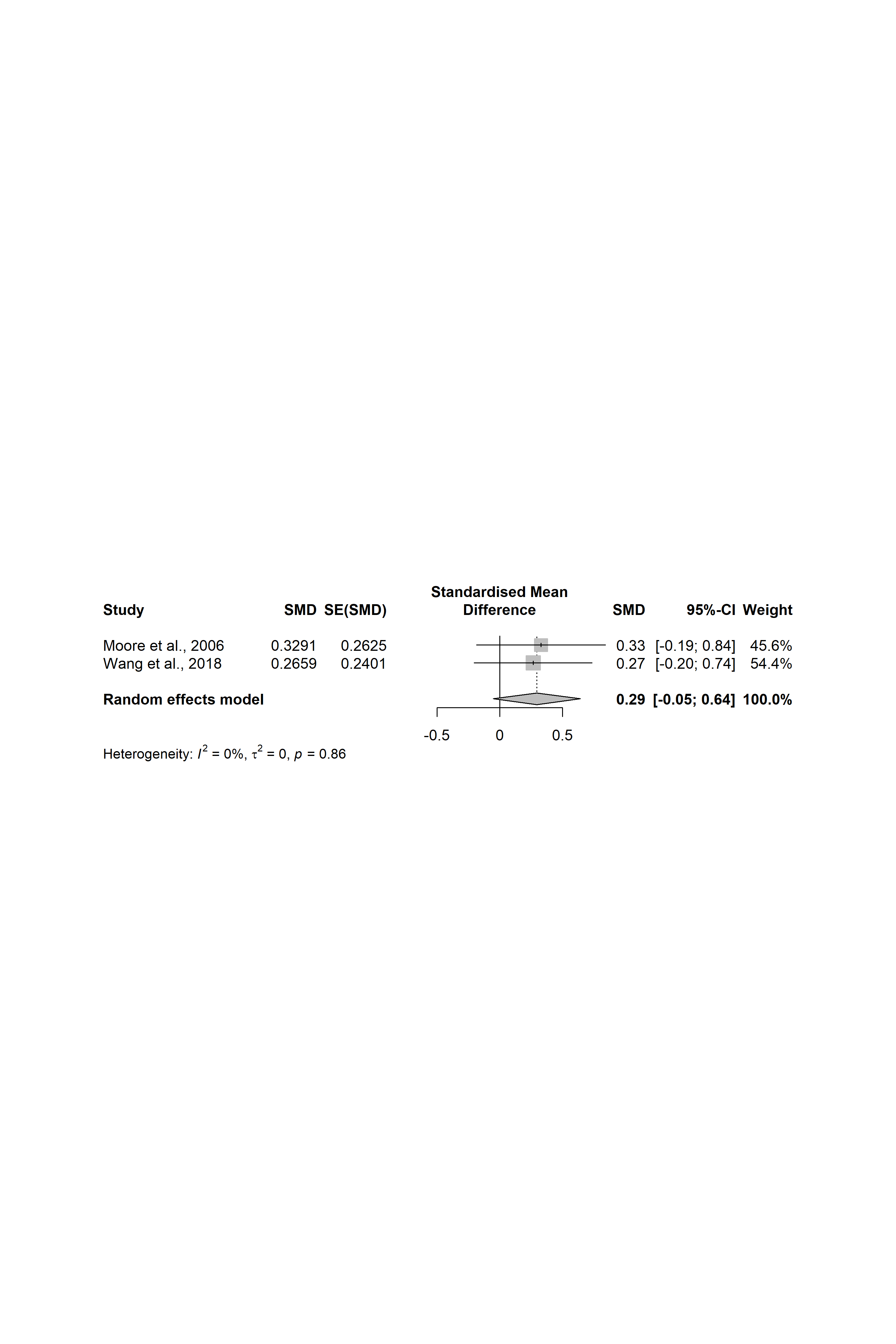


1. Depression vs Schizophrenia


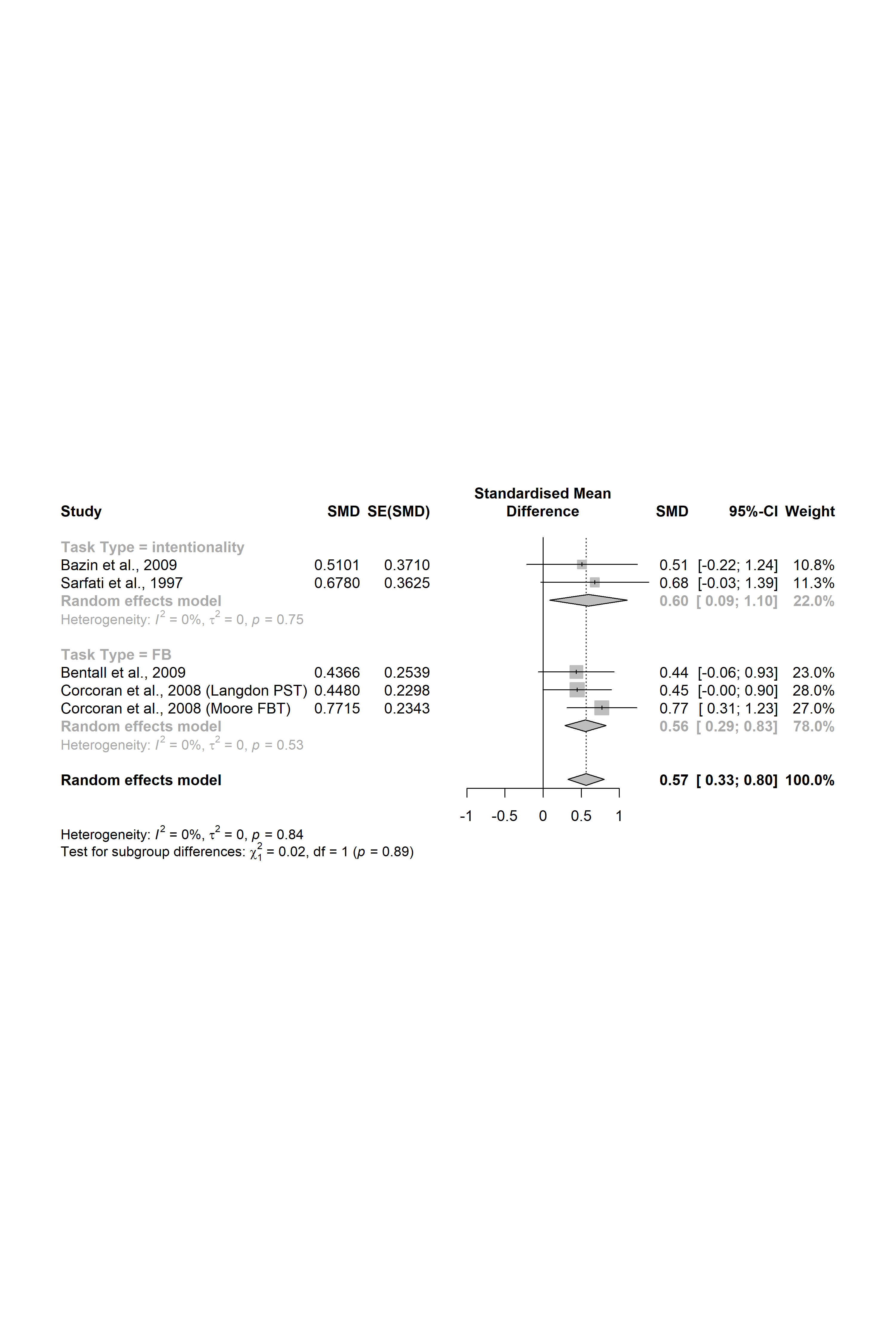


1. FHR-S vs Schizophrenia


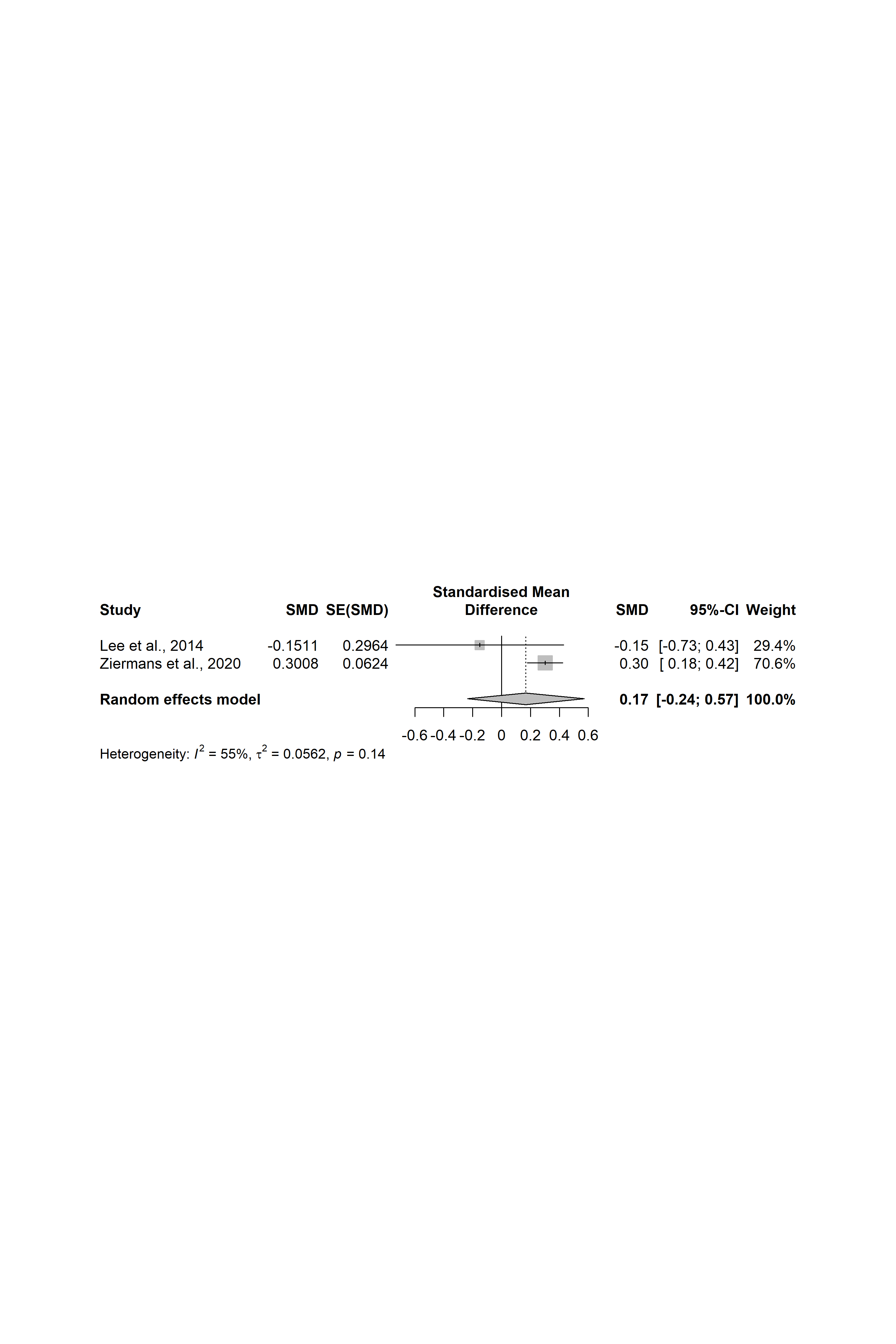


# Supplementary Figure 2. Funnel plots of theory of mind with static illustrations across psychiatric conditions compared to healthy controls, and between conditions.

1. ASD vs HC


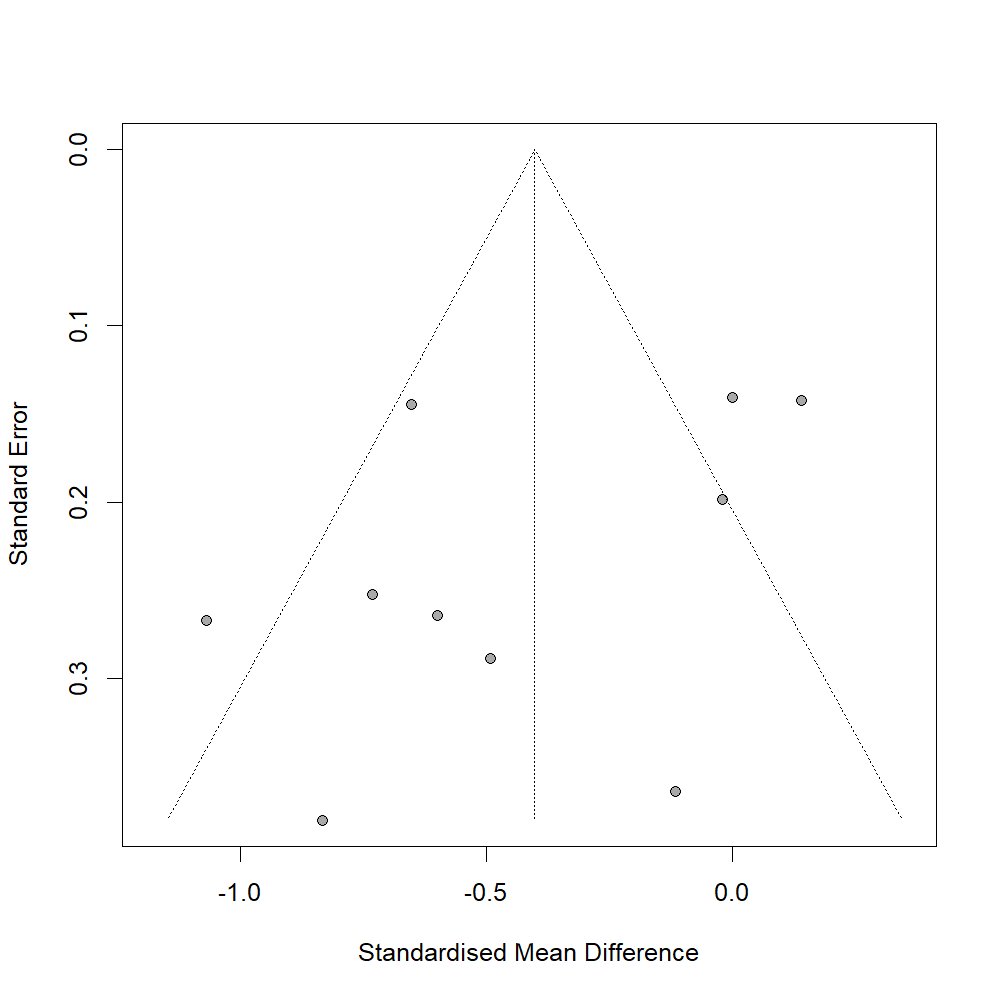


1. Bipolar disorder vs HC


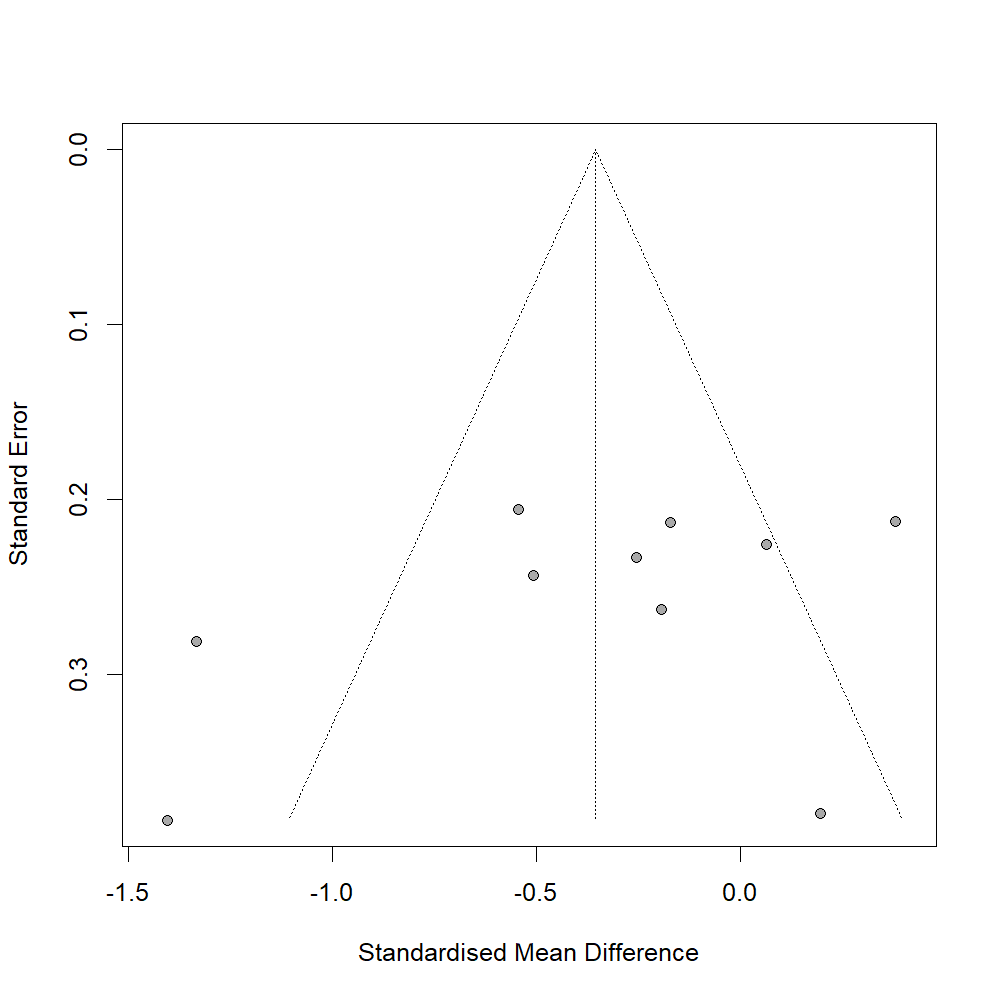


1. BPD vs HC


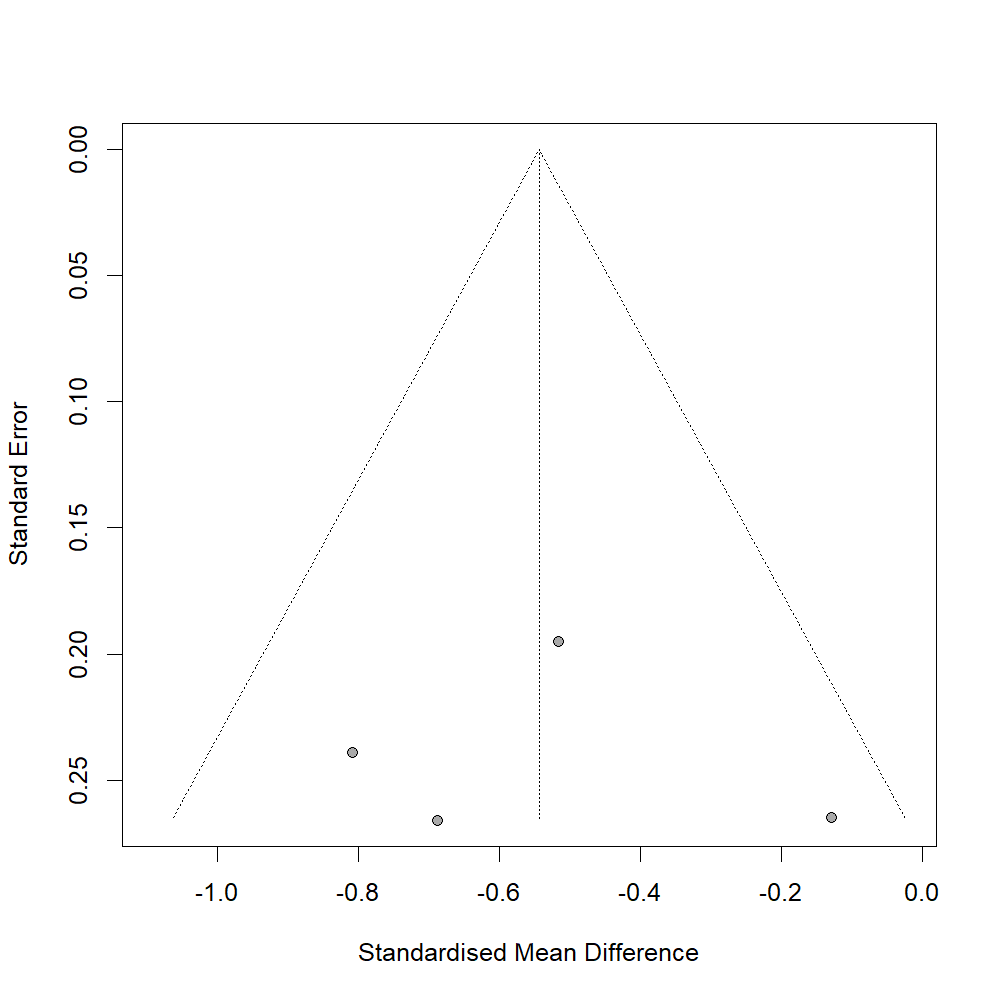


1. CHR vs HC


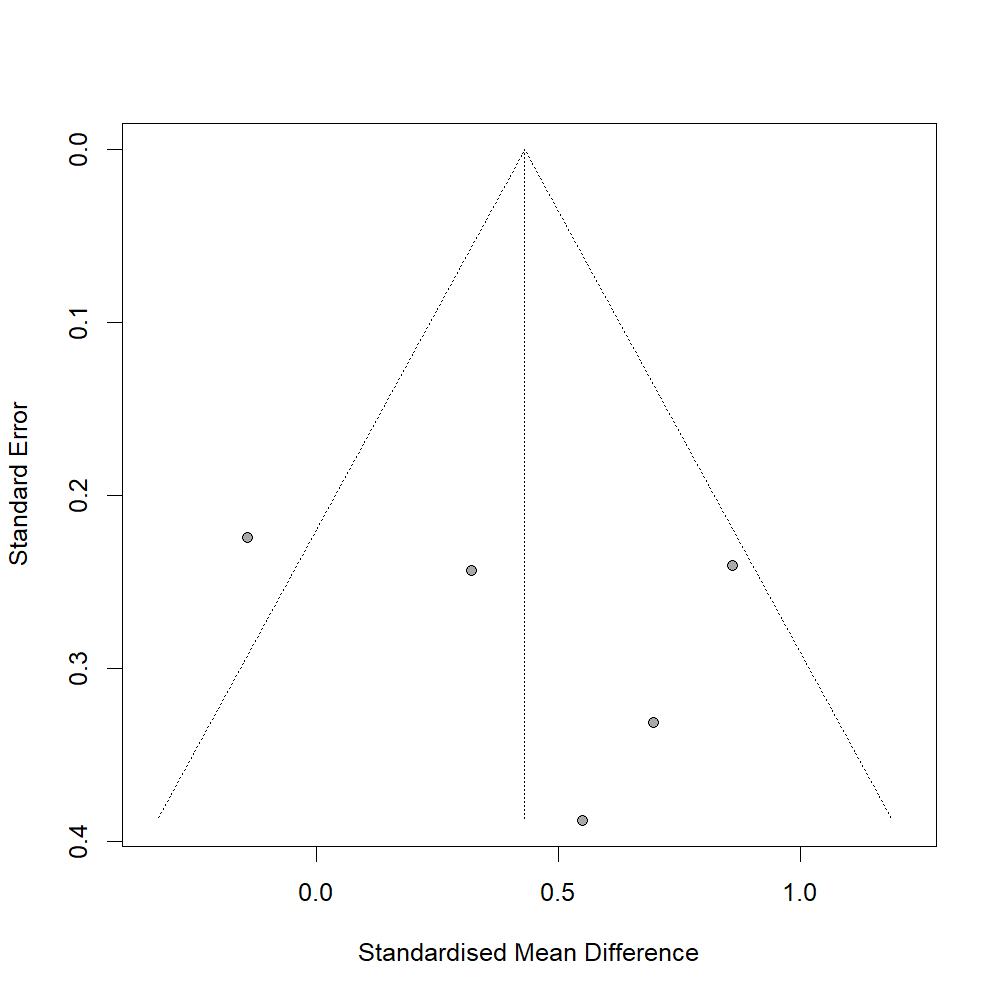


1. Depression vs HC


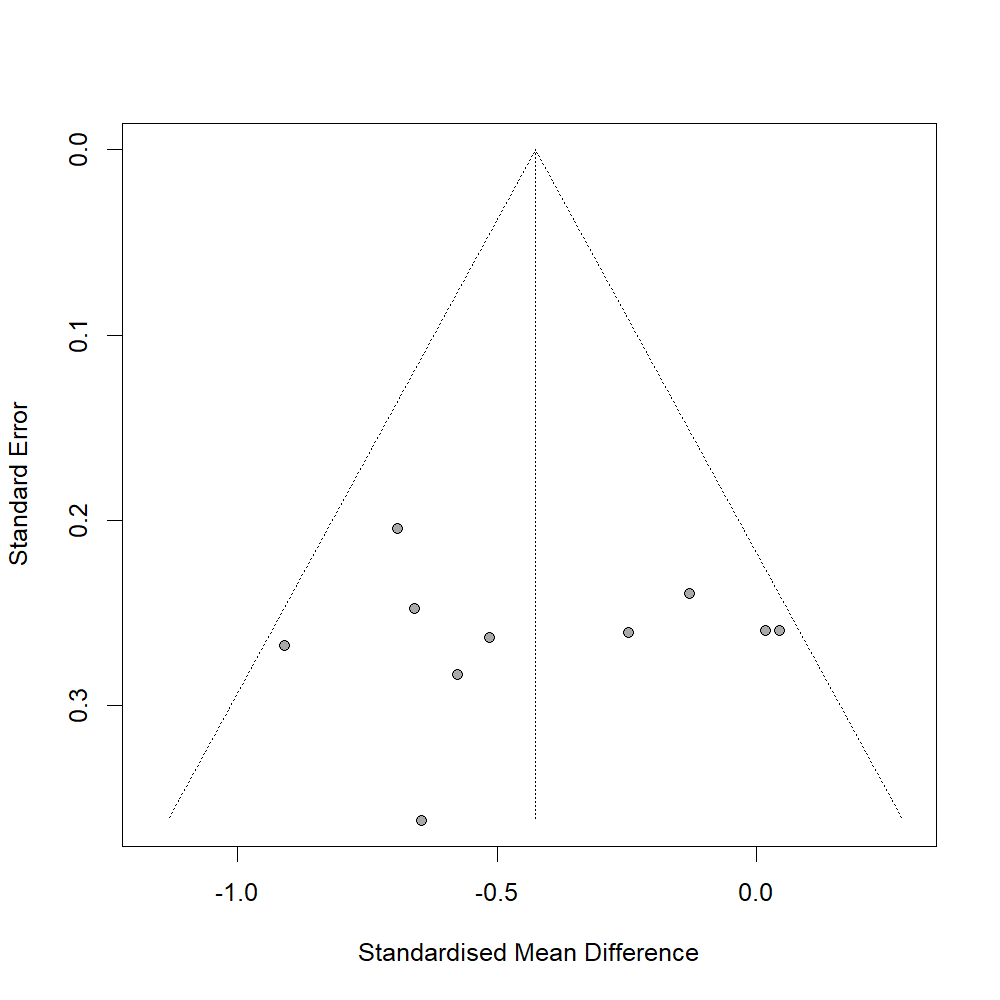


1. Early schizophrenia vs HC


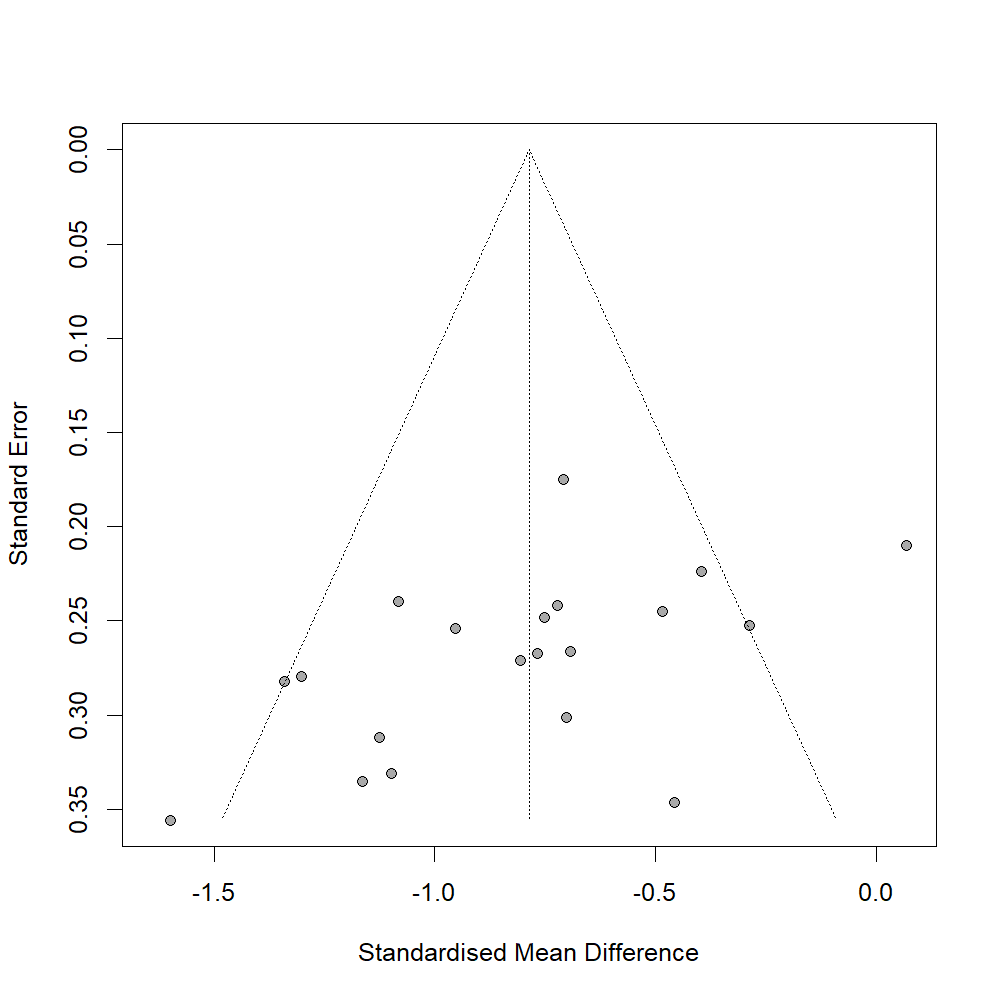


1. FHR-S vs HC


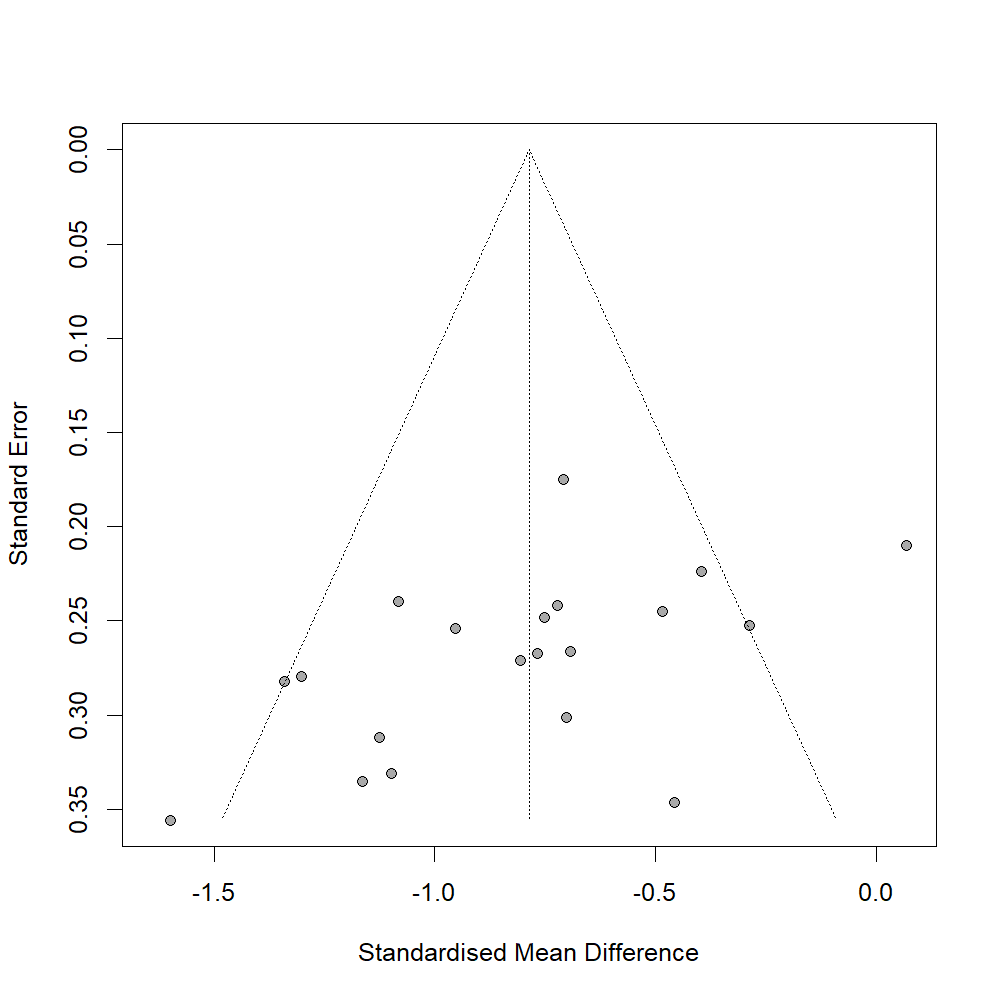


1. OCD vs HC


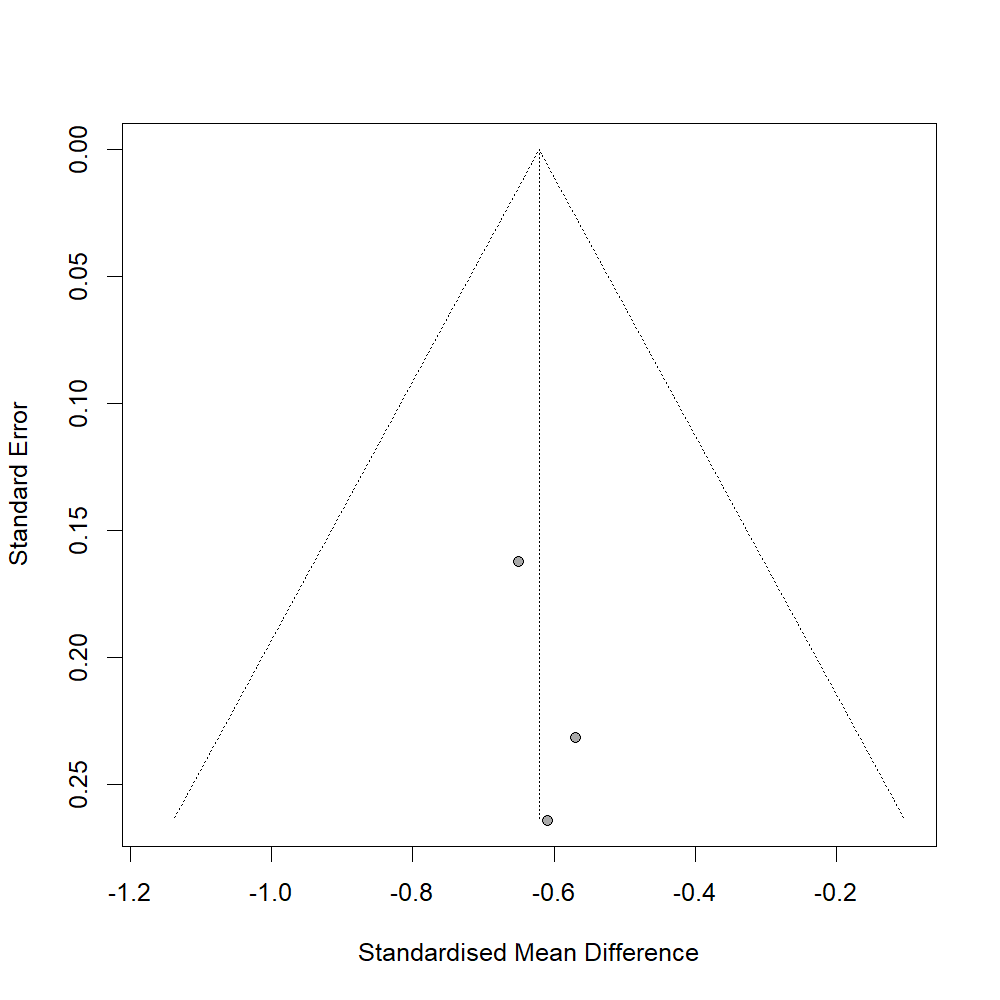


1. Schizophrenia vs HC


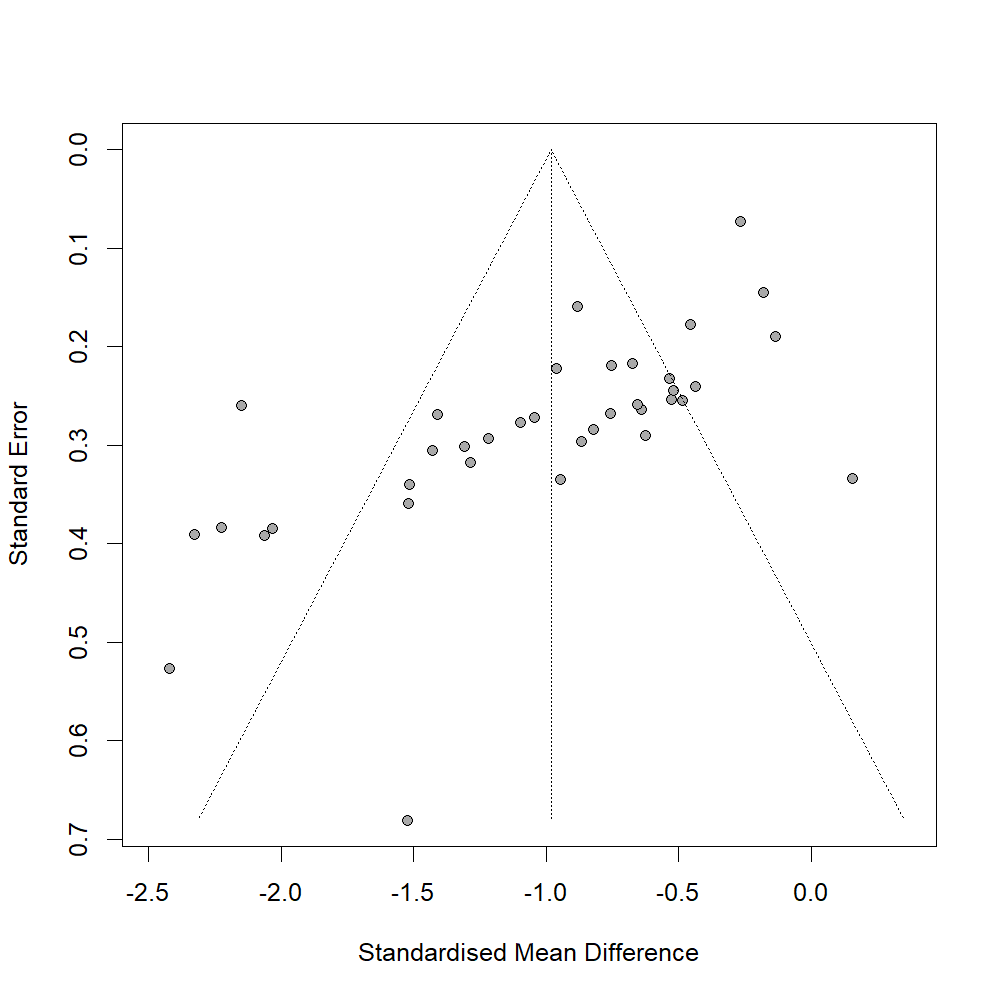


1. CHR vs Early schizophrenia


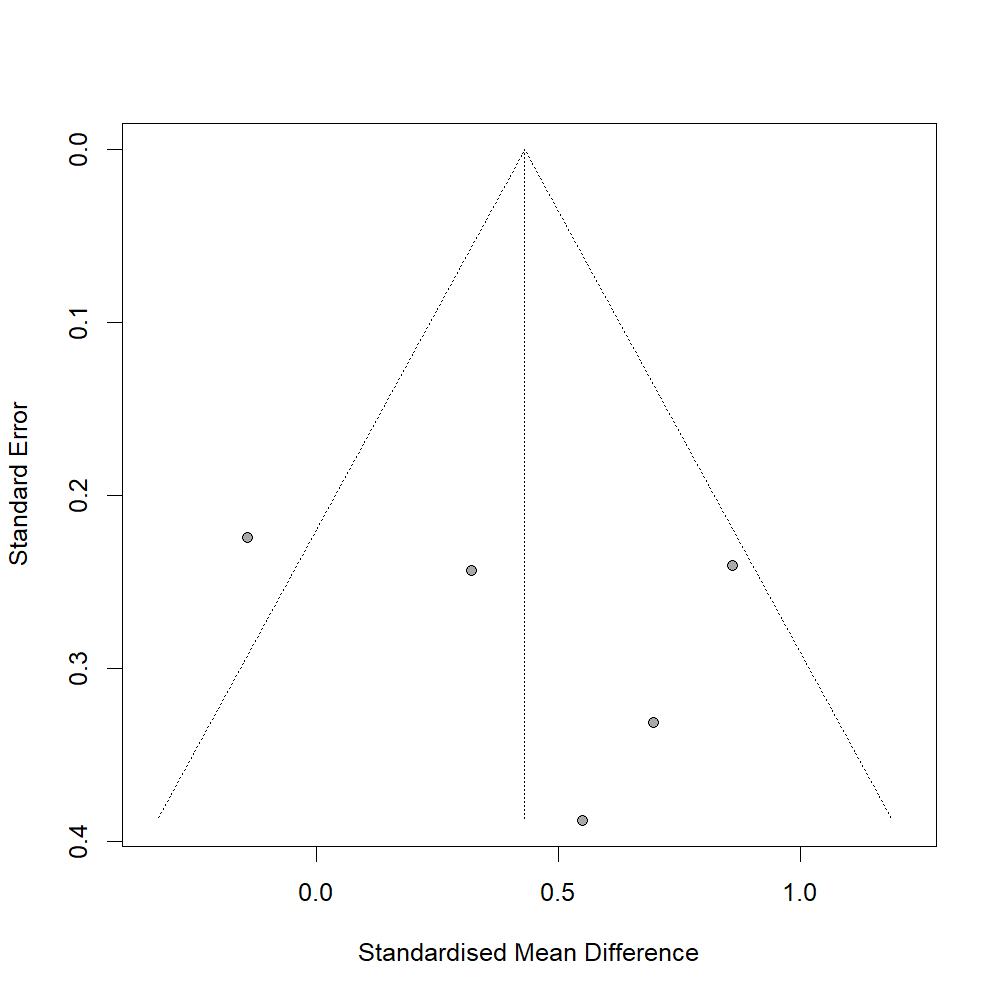


1. Depression vs Schizophrenia


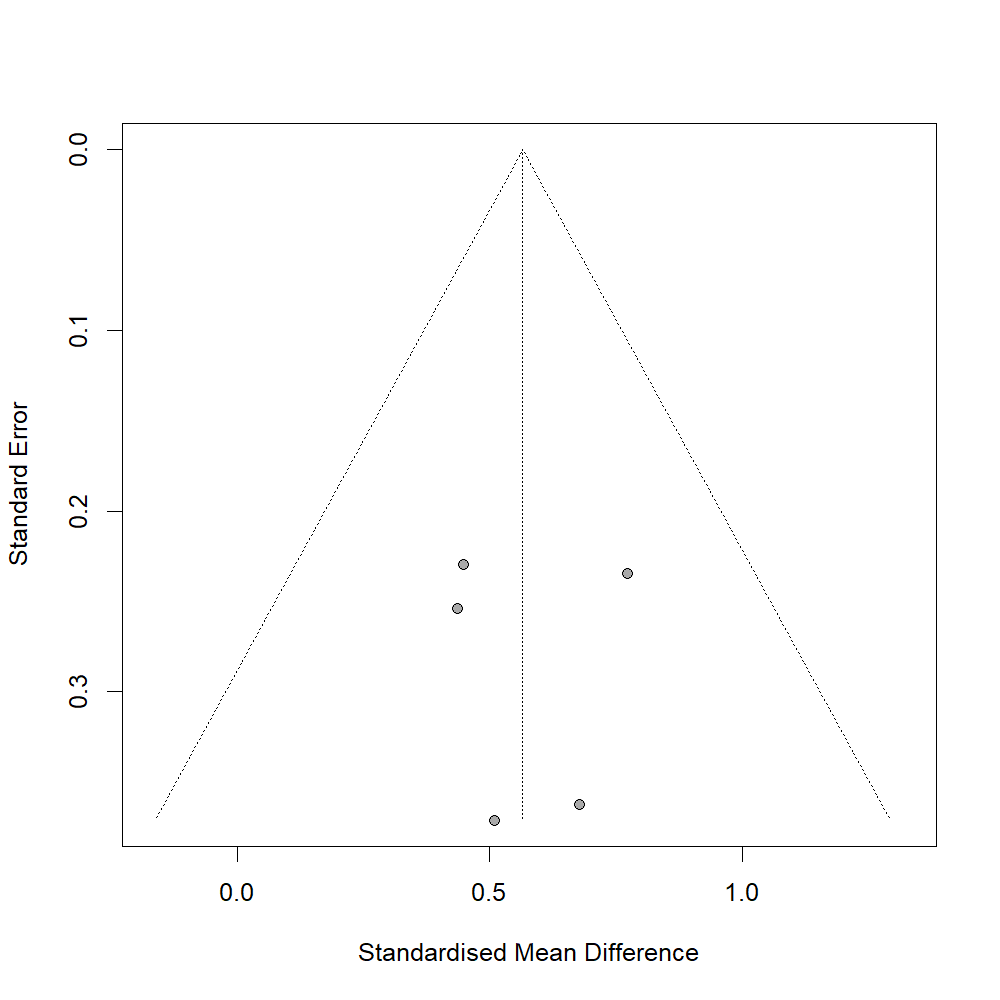


# Supplementary Figure 3. Comparison-adjusted funnel plots and publication bias assessment for network meta-analysis

A. Overall Network Analysis

Funnel plot for all included studies (k = 133)


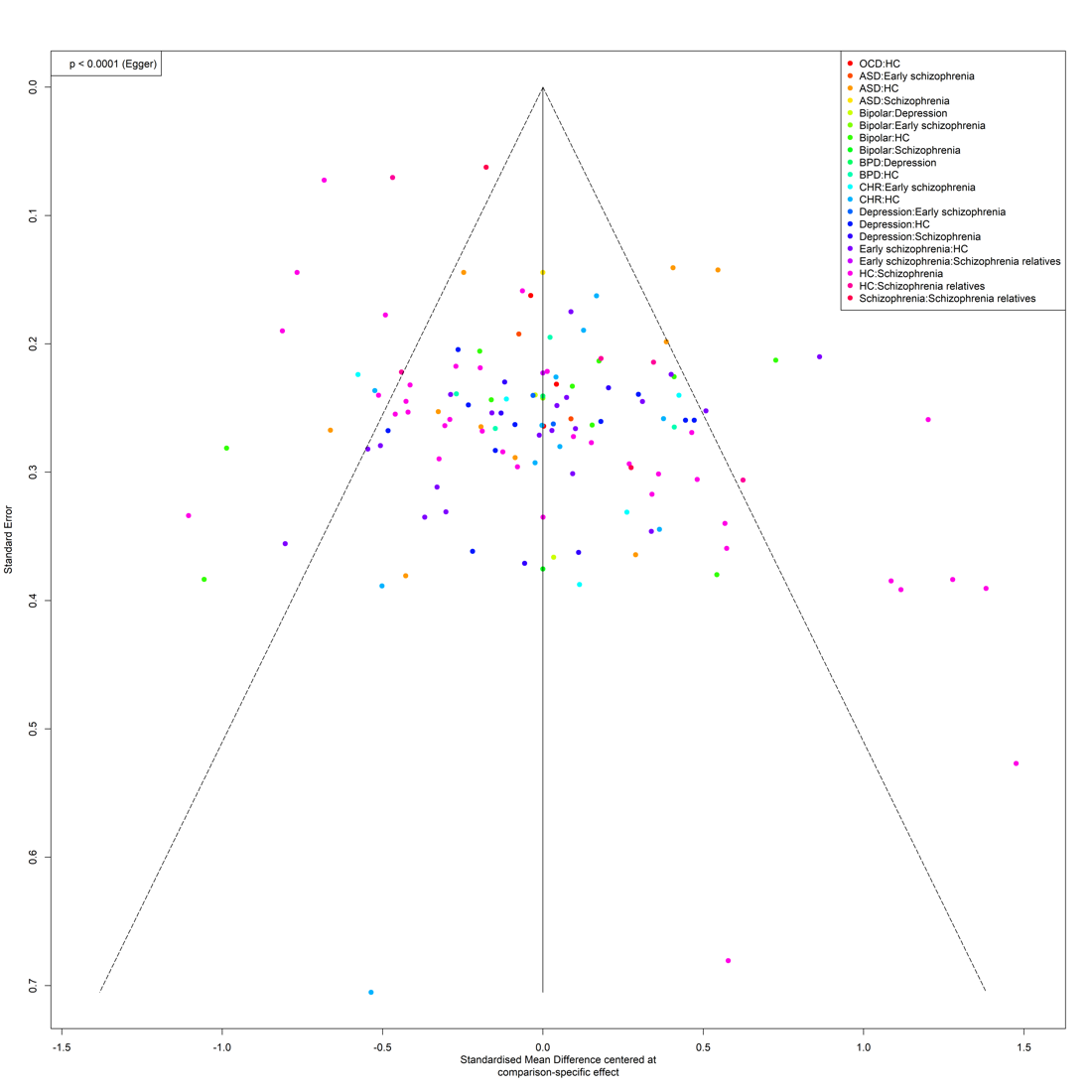


Linear regression test of funnel plot asymmetry

Test result: t = 4.81, df = 131, p-value < 0.0001

Bias estimate: 1.7453 (SE = 0.3626)

B. False Belief

Funnel plot for False Belief studies (k = 57)


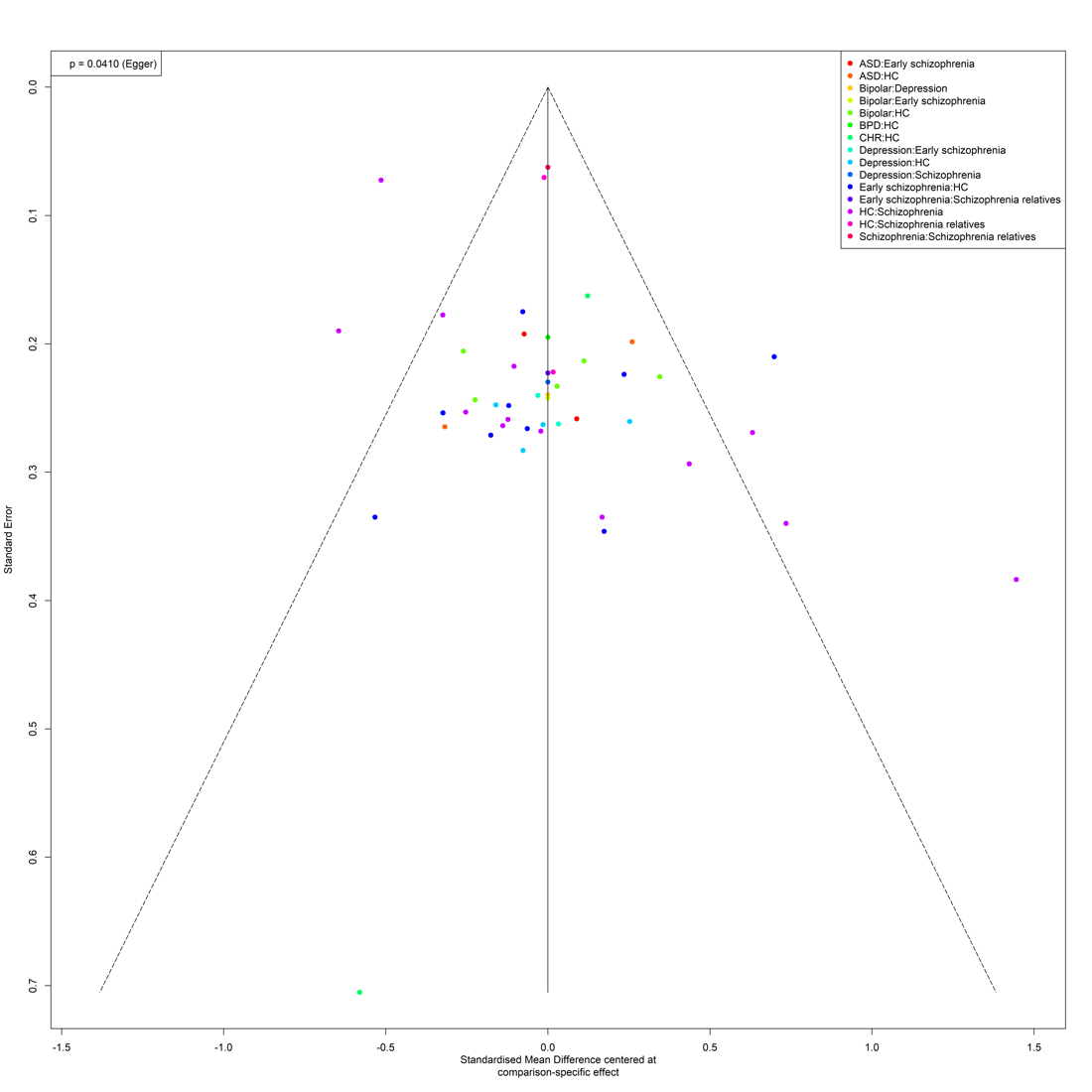


Linear regression test of funnel plot asymmetry

Test result: t = 2.08, df = 55, p-value = 0.0422

Bias estimate: 0.8564 (SE = 0.4117)

C. Intentionality

Funnel plot for Intentionality studies (k = 61)


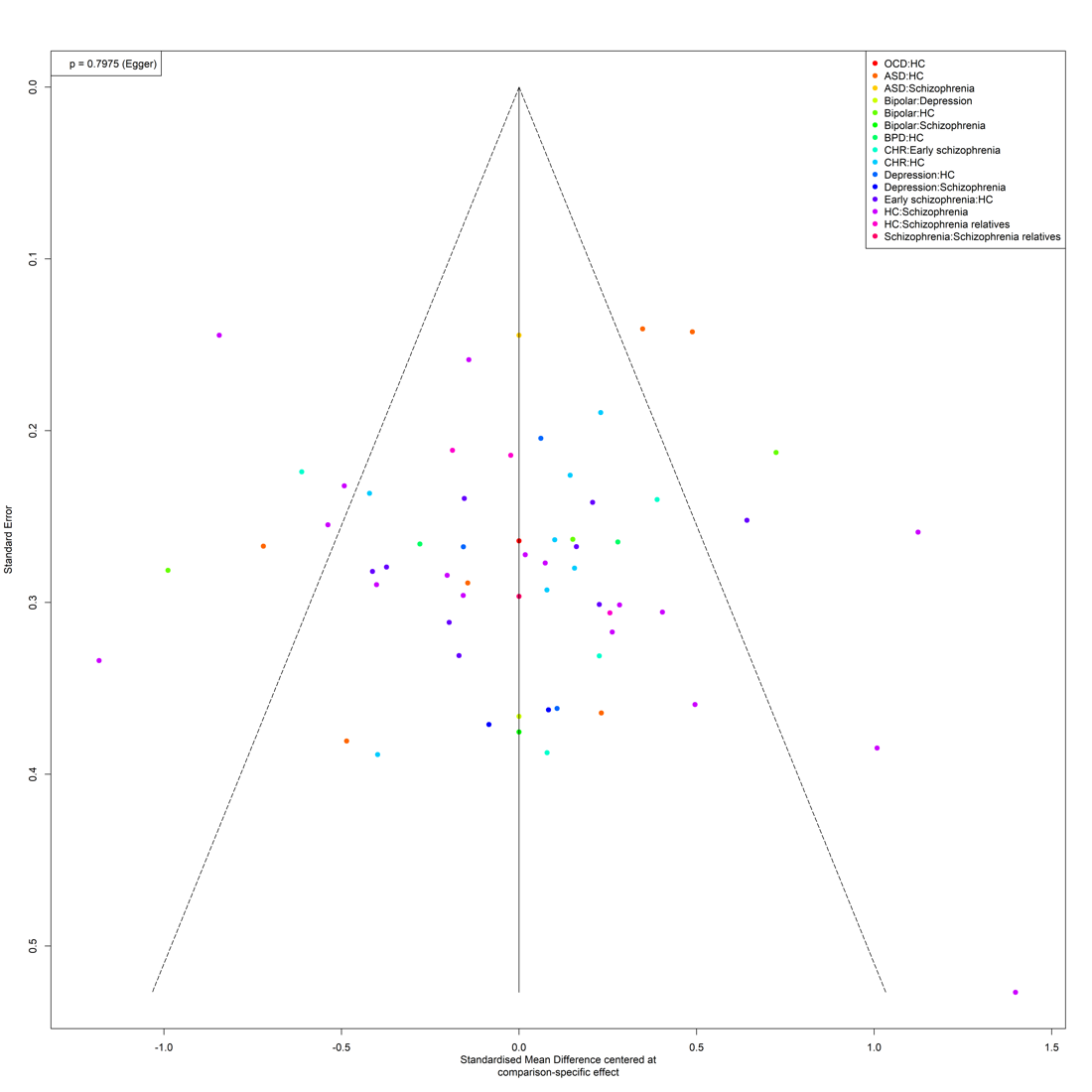


Linear regression test of funnel plot asymmetry

Test result: t = 0.26, df = 59, p-value = 0.7975

Bias estimate: 0.2060 (SE = 0.7993)

Note. Comparison-adjusted funnel plots display the difference between study-specific effect sizes and the comparison-specific summary effect (y-axis) against the study precision (x-axis). Asymmetry in the funnel plot may indicate publication bias or other small-study effects.
